# Small Non-Coding-RNA in Gynecological Malignancies

**DOI:** 10.3390/cancers13051085

**Published:** 2021-03-03

**Authors:** Shailendra Kumar Dhar Dwivedi, Geeta Rao, Anindya Dey, Priyabrata Mukherjee, Jonathan D. Wren, Resham Bhattacharya

**Affiliations:** 1 Department of Obstetrics and Gynecology, University of Oklahoma Health Sciences Center, Oklahoma City, OK 73104, USA; shailendra-dwivedi@ouhsc.edu (S.K.D.D.); Anindya-Dey@ouhsc.edu (A.D.); 2 Department of Pathology, University of Oklahoma Health Sciences Center, Oklahoma City, OK 73104, USA; Geeta-Rao@ouhsc.edu (G.R.); Priyabrata-Mukherjee@ouhsc.edu (P.M.); 3 Peggy and Charles Stephenson Cancer Center, University of Oklahoma Health Sciences Center, Oklahoma City, OK 73104, USA; 4 Biochemistry and Molecular Biology Department, University of Oklahoma Health Sciences Center, Oklahoma City, OK 73104, USA; Jonathan-Wren@omrf.org; 5 Oklahoma Center for Neuroscience, University of Oklahoma Health Sciences Center, Oklahoma City, OK 73104, USA; 6 Genes & Human Disease Research Program, Oklahoma Medical Research Foundation, Oklahoma City, OK 73104, USA; 7 Department of Cell Biology, University of Oklahoma Health Science Center, Oklahoma City, OK 73104, USA

**Keywords:** small non-coding-RNA, gynecological malignancies, microRNAs (miRs), P-Element induced wimpy testis interacting (PIWI) RNAs (piRNAs), tRNA-derived small RNAs, ovarian cancer, endometrial cancer, cervical cancer

## Abstract

**Simple Summary:**

Gynecologic malignancies are among the leading cause of female mortality worldwide, and their management is complicated by late diagnosis and acquired therapy resistance. Although altered DNA code, leading to aberrant protein expression, is indispensable for cancer initiation and progression, from the current literature it is clear that, not only proteins, but also noncoding RNA, which does not translate into proteins, can also be instrumental. Based on their size, noncoding RNA, are further classified into long and small noncoding RNA. Here, we have comprehensively reviewed the literature about the role of small noncoding RNAs in gynecological malignancies, and discussed how these small noncoding RNA can be vital for diagnosis and therapy.

**Abstract:**

Gynecologic malignancies, which include cancers of the cervix, ovary, uterus, vulva, vagina, and fallopian tube, are among the leading causes of female mortality worldwide, with the most prevalent being endometrial, ovarian, and cervical cancer. Gynecologic malignancies are complex, heterogeneous diseases, and despite extensive research efforts, the molecular mechanisms underlying their development and pathology remain largely unclear. Currently, mechanistic and therapeutic research in cancer is largely focused on protein targets that are encoded by about 1% of the human genome. Our current understanding of 99% of the genome, which includes noncoding RNA, is limited. The discovery of tens of thousands of noncoding RNAs (ncRNAs), possessing either structural or regulatory functions, has fundamentally altered our understanding of genetics, physiology, pathophysiology, and disease treatment as they relate to gynecologic malignancies. In recent years, it has become clear that ncRNAs are relatively stable, and can serve as biomarkers for cancer diagnosis and prognosis, as well as guide therapy choices. Here we discuss the role of small non-coding RNAs, i.e., microRNAs (miRs), P-Element induced wimpy testis interacting (PIWI) RNAs (piRNAs), and tRNA-derived small RNAs in gynecological malignancies, specifically focusing on ovarian, endometrial, and cervical cancer.

## 1. Introduction

### 1.1. Gynecologic Cancers

In the United States, in 2020, gynecologic cancers constituted ~6% of all cancer patients, accounting for ~5.3% of all cancer related deaths. Typically, the primary, and most effective, treatment for these cancers is optimal surgery; however, surgery is effective only if the disease is diagnosed at an early stage. Due to recent clinical advances, including the availability of effective screening tools, both endometrial and cervical cancer are increasingly likely to be diagnosed at an early stage. Disappointingly, ovarian cancer is designated the “silent killer”, because it is often diagnosed at an advanced stage where curative treatment is difficult; treatment of ovarian cancer is often further challenged by recurrence and acquired drug resistance. These malignancies have been reviewed in detail elsewhere [1,2,3,4], and a brief summary is presented in the following sections:

#### 1.1.1. Cervical Cancer

Cervical cancer is the fourth most commonly diagnosed malignancy worldwide [5], constituting approximately 50% of all malignancies of the female reproductive system. Cervical cancer is most often diagnosed in women between the ages of 35 and 44 years, although the average age at diagnosis is 50 years. Overall, ~85% of new cases, as well as deaths, occur in developing countries. In the United States over the last twenty years the case rate has fallen from 8.9 to 6.6 per 100,000 population, mainly due to increased testing. The most common patho-histological form of cervical cancer is planocellular (squamous cell carcinoma), which represents over 90% of all cancers at this site [6]. Approximately 6% are adenocarcinomas and less than 2% are adenosquamous (mixed) carcinomas [7]; the remaining are rare carcinomas and cervical sarcomas [8]. Based on the degree of differentiation, cervical cancers are divided into, unknown (g_x_), good (g_1_), medium (g_2_), poor (g_3_), and undifferentiated (g_4_) grades. Hematogenic metastasis is relatively rare and occurs quite late, such that cervical cancer remains a pelvic disease for a prolonged period [9]; the most common sites for metastasis are the lungs and the liver. Bone metastases are rare, and when found they are usually in the vertebrae of the spinal column or, even more rarely, the long bones of the lower extremities. Most cervical cancer cases are caused by the sexually transmitted human papillomavirus (HPV), which is present in more than 90% of tumors [10]. Harald zur Hausen identified HPV in cervical cancer patients and, received the Nobel Prize in Physiology or Medicine in 2008 for his study of the etiology of cervical cancer and the role of HPV in its genesis [11]. The HPV-E6 protein complexes with cellular proteins, ubiquitin-protein ligase E3A (E6AP), and p53, facilitating p53 degradation via the ubiquitin dependent proteolytic system [12], and leading to invasive cervical cancer.

#### 1.1.2. Endometrial Cancer

More than 90% of uterine cancers occur in the endometrium, and endometrial cancer is the most commonly diagnosed gynecologic malignancy in developed countries [13]. In the USA, a projected 65,620 patients will be diagnosed with, and approximately 12,590 women will die from, endometrial cancer in 2020. The number of women diagnosed with endometrial cancer is increasing, largely due to increased obesity rates; obesity is an important risk factor for endometrial cancer. From 2007 to 2016, the number of white women diagnosed with endometrial cancer increased annually by 1%, while in the same timeframe diagnoses in black women increased by 2%. Nevertheless, 67% of women with endometrial cancer are diagnosed at an early, and more readily treatable, stage. Most endometrial cancers are sporadic; ~5% are considered hereditary and caused by mutations in the DNA mismatch repair genes [14]. From 2008 to 2017, deaths from uterine cancer increased by approximately 2%.

Traditionally, endometrial cancers are classified by histo-morphologic features, and stratified into the more common, lower risk, estrogen-driven type I cancers, and the less common, more aggressive, non-estrogen-driven type II cancers [15]. Type I endometrial cancer arises from pre-neoplastic lesion hyperplasia that has undergone unchecked estrogenic stimulation [16], and Type II carcinomas develop from atrophic endometrium and are frequently serous or clear-cell adenocarcinomas. The most comprehensive molecular study of endometrial cancer to date was provided by The Cancer Genome Atlas (TCGA) project, which included a combination of whole genome sequencing, exome sequencing, microsatellite instability (MSI), and copy number analysis [17]. This molecular information was used to classify 232 endometrioid and serous endometrial cancers into four groups, *POLE* ultra-mutated, MSI hyper mutated, copy-number (CN) low, and CN high, that correlate with progression-free survival (reviewed in detail by McAlpine [18]).

#### 1.1.3. Ovarian Cancer

Ovarian cancer remains a major cause of both morbidity and mortality in women, with little improvement in survival rates over the past four decades. Ovarian tumors are divided into three clinico-pathological subtypes with distinct histopathological features: epithelial, sex cord-stromal, and germ cell tumors. Of these, epithelial ovarian cancer (EOC) is the most common, representing 80–85% of ovarian cancers [19]. Based on histological and morphological differences, EOC is classified into five major categories: high-grade serous, low-grade serous, mucinous, endometrioid, and clear-cell carcinoma. High-grade serous ovarian cancer (HGSOC) is the most frequently seen and lethal histotype, causing nearly 75% of all EOC-related mortalities [19]. Historically, HGSOC was thought to originate from the ovarian surface epithelium; however, recent studies have indicated that the majority of advanced HGSOC may arise from the fallopian tube fimbriae [20]. Within the most common subtype, HGSOC, TP53 is mutated in over 90% of patients. Advances in next-generation sequencing have revealed that mutations within the DNA repair pathways, including BRCA1 and BRCA2, are common in about 50% of HGSOC patients; this has led to the development of a breakthrough therapy targeting poly ADP ribose polymerase (PARP) via inhibitors. These vulnerabilities of HGSOC are being explored, with recent reports indicating an interplay between *BRCA1*-mutation status and progesterone signaling. Therefore, treatment with anti-progestins could be an effective nonsurgical, prophylactic option for ovarian and breast cancer prevention in these high-risk women [21].

Current diagnostic methods for the detection and monitoring of ovarian cancer include pelvic examination, transvaginal ultrasound, and measurement of the serum biomarker carbohydrate antigen 125 (CA125) [22], human epididymis protein 4 (HE4) [23] Wnt/beta-catenin [24], and p53 [25]; these methods have certainly improved outcomes, but have limitations and lack adequate sensitivity. Ovarian cancer is asymptomatic in its early stages making early diagnosis difficult. About 75% of patients are diagnosed at stage III or IV with extensive metastasis in the peritoneal cavity, and have a 5-year survival rate of less than 40% [26]. In contrast, patients who are diagnosed at stages I or II, have 5-year survival rates of 70–90%. The high mortality rate of ovarian cancer can be partly attributed to its detection at late stages.

While current research has provided tools for improved diagnosis and management of gynecologic malignancies, the continuing socio-economic and racial disparities in gynecologic cancers highlight the need for further studies to develop sensitive diagnostics for early detection and efficacious targeted therapy with reduced toxicity. However, the potential roles of small ncRNAs in regulating the proteome via interaction with mRNA, DNA, and proteins have not been fully explored.

## 2. Small ncRNAs

The term non-coding RNA refers to a large group of endogenous RNA molecules that have no protein coding capacity. The Encyclopedia of DNA Elements (ENCODE) consortium reports that around 70% of the genome is transcribed for RNA molecules [27] that have no protein coding capacity. Rather than being mere transcriptional noise, these molecules are powerful regulators of gene expression and function as structural, catalytic, and regulatory RNAs [28,29,30,31]. Non-coding RNAs are further divided into small non-coding RNAs (sncRNAs) with sizes <200 nt (e.g., miR, piRNA, and tiRNA), and long non-coding RNAs (lncRNA) with sizes ≥200 nt (e.g., lincRNA, NAT). The defining features of sncRNAs are their short length, their association with members of the argonaute (Ago) family of proteins, and that they usually downregulate or silence target gene expression. Beyond these defining features, different sncRNA classes guide diverse and complex schemes of gene regulation [32].

The aberrant expression of sncRNAs is associated with various cellular dysfunctions and disease states. Increasing evidence suggests that multiple sncRNA groups play important roles in cancer initiation, progression, and associated pathophysiology. Clinically, sncRNA aberrations show high diagnostic and prognostic value. With improved understanding of the nature and roles of non-coding RNAs, it is believed that we can develop cancer therapeutics that act via the modulation of such RNA molecules. Advances in in-vivo nucleic acid delivery methods and in-silico approaches have prompted the development of agents that may disrupt the oncogenic functions of non-coding RNAs. In this review, we will briefly discuss the roles of piRNA, tRNA, and miR in endometrial, cervical, and ovarian cancers.

### 2.1. piRNA

**Figure 1:**
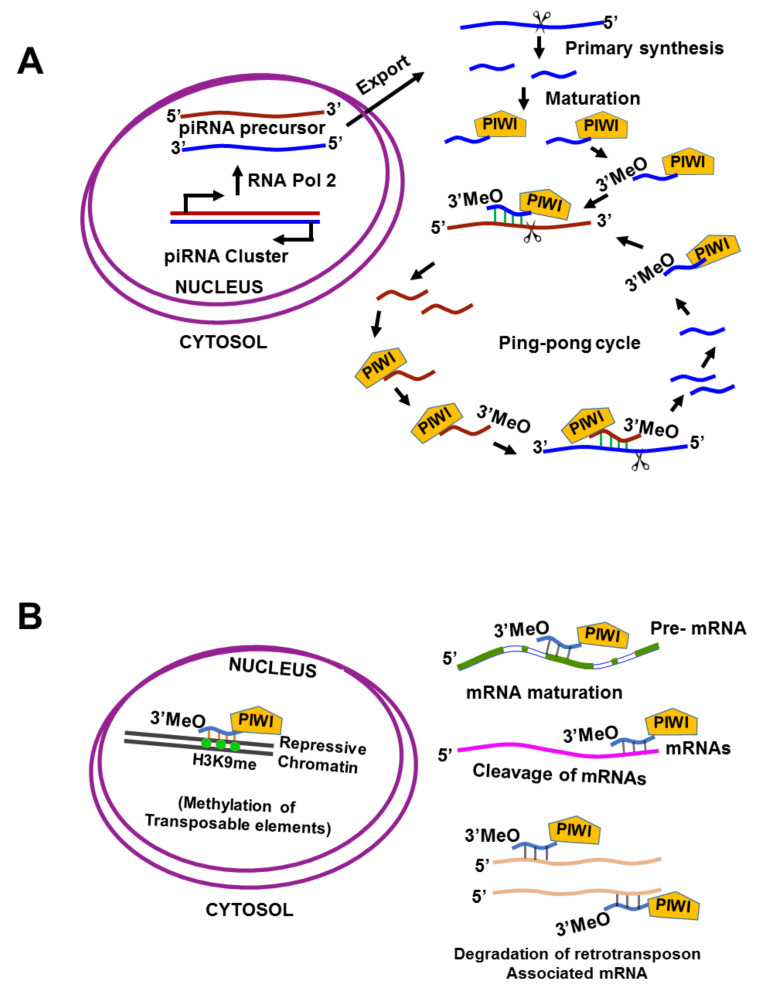
Biogenesis and function of piRNAs. (**A**) Biogenesis of piRNAs: Biogenesis of piRNA occurs through primary and secondary pathways (ping-pong cycle). In the primary pathway, piRNA precursors are transcribed from piRNA clusters by RNA polymerase 2 (RNA Pol2). Antisense primary piRNAs are cleaved, trimmed into short fragments, and their 3′ ends are 2′-*O*-methylated and then loaded onto PIWI family proteins. In the secondary amplification pathways, also known as the ping-pong cycle, PIWI proteins associate with antisense piRNA and cleave piRNA precursors in the sense strand, or PIWI proteins associate with sense piRNA and cleave antisense piRNA precursors in the sense strand. The incorporated RNA is therefore processed into a mature secondary piRNA by trimming and modification, likely by the same mechanisms that generate a primary piRNA. (**B**) The biological functions of piRNAs: In the nucleus, PIWI–piRNA complexes can repress the transposon expression by methylation of the transposon region or chromatin modification around the transposon region. In the cytoplasm, piRNAs can facilitate mRNA maturation, cause cleavage of mRNAs through a miRNA-like mechanism, and degrade retrotransposon-associated mRNAs.

P-Element induced wimpy testis interacting (PIWI) RNAs (piRNAs) are sncRNAs of approximately 24–31 nucleotides; they have a 5′-terminal uridine or tenth position adenosine bias, lack clear secondary structure motifs, and interact with PIWI, which are nuclear RNA-binding proteins. piRNAs were initially described in germline cells, but emerging evidence reveals they are expressed in a tissue-specific manner among multiple human somatic tissue types, as well as in cancer [33]. piRNAs were first identified in fly testis as a novel class of “long siRNAs” that silence Stellate, a multi-copy gene on the *Drosophila melanogaster*, X chromosome [34]. Depending on the source, piRNAs are divided into three groups: lncRNA-derived piRNAs, mRNA-derived piRNAs, and transposon-derived piRNAs. Transposon-derived piRNAs are transcribed from two genomic strands, thus producing both piRNAs and antisense piRNAs; mRNA-derived piRNAs are usually derived from 3′ untranslated regions (UTRs), and lncRNA-derived piRNAs are produced from the entire transcript [35]. These precursors are usually generated by specific genomic locations containing repeating elements, and usually occur independently of the dicer. In addition, piRNAs require post-transcriptional modification to become mature piRNAs; piRNAs bear 2′-*O*-methyl-modified 3′ termini and guide PIWI-clade argonautes (PIWI proteins) rather than the AGO-clade proteins, which function in the miRNA and siRNA pathways [36,37,38,39,40,41,42,43] (Figure 1A).

PIWI proteins are germline-specific Ago family members [44] that are essential for germline development and gametogenesis in animals [39,45,46,47,48]. The PIWI protein family shares a conserved structure and function across multiple organisms [49], including fruit fly (PIWI, aubergine, and AGO3 proteins) [43], mouse (MILI, MIWI, and MIWI2) [39,50,51,52], human (HILI, HIWI1, HIWI2, and HIWIL3) [42,43,53,54], zebrafish (ZILI and ZIWI) [55], and nematode (PRG-1 and PRG-2) [56]. The classical function of PIWI/piRNAs is to maintain genomic integrity by repressing the mobilization of transposable elements, and to regulate the expression of downstream target genes via transcriptional or post-transcriptional mechanisms, including epigenetic silencing of transposons through DNA methylation [33,44] and H3K9 tri-methylation through recruitment of heterochromatin protein 1 (HP1) and histone methyltransferases (HMTs) [57]. piRNAs regulate mRNA levels by complementary sequence binding to the 3′UTR, and a protein’s stability by binding to it (Figure 1B). For instance, piRNA-54265 binds with the PIWIL2 protein and promotes the formation of the PIWIL2/STAT3/phosphorylated-SRC (p-SRC) complex, which activates STAT3 signaling and promotes the proliferation, metastasis, and chemo-resistance of colorectal cancer cells.

Recently the reactivation of PIWI protein expression, primarily PIWI-like proteins (PIWIL1, PIWIL2, PIWIL3, and PIWIL4), has been identified in various malignancies [58,59,60,61].

Increasing evidence suggests that PIWI proteins are linked to the hallmarks of cancer, such as cell proliferation, anti-apoptosis, genomic instability, invasion, and metastasis. Due to their restricted expression PIWI are classified as cancer/testis antigen (CTA) and are considered as excellent targets for diagnostic and prognostic biomarkers, and immunotherapy [62]. The expression of piRNA or PIWI protein in non-germline cancers is in line with the well-established phenomenon of cancer/germline genes, which describes the aberrant expression of germline-specific genes in non-germline cancers [61,63,64,65,66]. This provides new possibilities for anticancer therapies through the targeting of PIWI proteins, and which may have fewer side effects due to their restricted expression.

#### 2.1.1. piRNA in Gynecological Cancers

Increasing functional evidence supports the involvement of piRNAs in the regulation of epigenetic changes in tumorigenesis [67,68,69], along with posttranscriptional mRNA and protein stability regulation. It has been suggested that the PIWI–piRNA complex contributes to cancer development and progression by promoting a stem-like state of cancer cells, or cancer stem cells (CSCs). It has been reported that CSCs represent the cells that have undergone epithelial–mesenchymal transition (EMT) and acquired metastatic capacities. Such epigenetic alterations allow cancer cells to adapt to changes in their microenvironment. Epigenetic global changes in cancer include DNA hypo-methylation, histone hypo-acetylation, and gene-specific DNA hyper-methylation, leading to oncogene activation (R-ras, cyclin D2) [70], and tumor suppressor silencing (RB1, p16) [71]. In cancer tissues, aberrantly expressed piRNAs implicate global hypo-methylation and local hyper-methylation as potential cancer-specific features [69,72].

Abnormal expression of piRNAs is emerging as a crucial regulator in cancer cell proliferation, apoptosis, invasion, and migration. In gynecologic malignancies the study of piRNA expression and their pathophysiological significance remains exploratory. Singh et al. [73] used RNA sequencing to identify piRNAs in normal ovary (pi RNA #219), endometrioid (pi RNA #256), and serous ovarian cancer (pi RNA #234). Although sample numbers used in the study were small, the authors reported 159 and 143 differentially expressed piRNAs in endometrioid and serous ovarian cancer, respectively. The differentially expressed piRNAs in endometrioid ovarian cancer were comprised of 74 upregulated and 77 down-regulated piRNAs, while those in serous ovarian cancer included 56 upregulated and 81 downregulated. Specific findings showed that piR-52207 was upregulated in endometrioid ovarian cancer, and piR-52207 and piR-33733 were increased in serous ovarian cancer [73]. Upregulated piR-52207 targets NUDT4, MTR, EIF2S3, and MPHOSPH8, which promote endometrioid ovarian cancer cell proliferation, migration, and tumorigenesis. In serous ovarian cancer, piR-33733 targets LIAS3′-UTRs, whereas piR-52207 binds ACTR10 and PLEKHA5 3′-UTRs and 5′-UTRs, leading to increased anti-apoptotic and decreased pro-apoptotic proteins. Thus, piR-52207 and piR-33733 promote ovarian cancer oncogenes via involvement in multiple cell-signaling pathways at the post-transcriptional level, supporting them as possible therapeutic targets for ovarian cancer [73].

In endometrial cancer, studies utilizing small-RNA sequencing and microarrays, have shown a significant difference in the expression pattern of piRNAs between normal, hyperplastic, and the neoplastic endometrium [74]. Utilizing the stringent thermodynamic parameters for RNA–RNA binding, the authors showed each piRNA to be complementary to a number of mRNAs, ranging from 28 to 308. In total, 1526 mRNA targets were predicted; differential expression analysis on paired sample groups revealed that 170 of the predicted 1526 were differentially expressed (|FC| ≥ 1.5 and *p*-value 0.001) in hyperplastic and/or tumor tissues [74]. In cervical cancer cell lines piR-651 has been shown to be upregulated [75] however, apart from this one example the details of piRNA status in cervical cancer remains largely unexplored.

#### 2.1.2. Future Perspectives

Cancer and germ cells share several essential characteristics, including high proliferation rates and self-renewal abilities; in addition, cancer cells may re-activate cancer testis antigen (CTA) genes, whose expression is usually restricted to the germline, and silenced in adult somatic tissues. The expression of germline genes in cancer reflects the aberrant activation of a silenced developmental program that leads to escape from cell death, immune evasion, and invasiveness, thus contributing to the molecular mechanisms of carcinogenesis [61]. Among cancer testis antigens, PIWI-like (PIWIL) genes, belonging to the Ago family, are frequently deregulated in several malignancies, including cervical [60,76,77], endometrial [78], and ovarian cancer [58,59,76], and these proteins, along with piRNA, are involved in various aspects of malignancy, and associated with advanced tumor stage and poor prognosis. Along with piRNA, the PIWI proteins are prominently expressed in cancer cells, making them both useful biomarkers for cancer diagnosis and possible druggable targets [79].

### 2.2. tRNA-Derived Small RNAs

**Figure 2:**
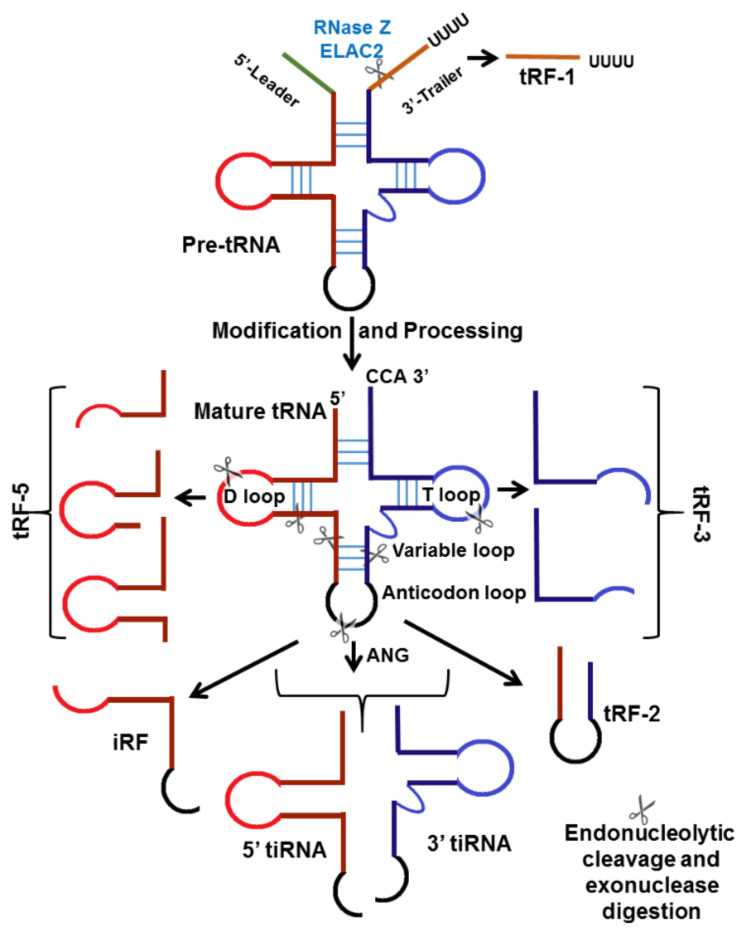
Biogenesis and function of tRNA-derived small RNAs: Different types of tRNA-derived RNA fragments produced from either pre-tRNAs or mature tRNAs. The tRF-1 series is produced by RNase Z (or ELAC2) cleavage of the pre-tRNA during tRNA processing. Mature tRNA can be cleaved in the anticodon loop by angiogenin (ANG) to produce the 5′-tiRNA and 3′-tiRNA series under stress conditions. tRF-2 is a tRNA fragment containing an anti-codon loop generated by an unknown cleavage method. Cleavage in the T-loop results in the production of the 3′-tRF series. The 5′-tRF series is derived from the 5′-end of mature tRNAs by endonucleolytic cleavage and exonuclease digestion in the D-loop.

Transfer RNA (tRNA), one of the most abundant cellular ncRNAs, is important for protein translation. Recent research has shown that tRNAs are not always the terminally differentiated molecules; fragments derived from tRNAs are a source of small regulatory RNAs, known as tRNA-derived small RNAs (tsRNAs) [80]. Based on the cleavage site, tsRNAs can be divided into two main types: (1) transfer RNA-derived RNA fragments (tRFs) approximately 14 to 30 nucleotides in length, and derived from mature or precursor tRNAs; and (2) tRNA halves or tiRNAs, 29 to 50 nucleotides in length, induced by stress, and produced by specific cleavage at the anticodon loop of mature tRNA. tRFs are further subdivided into tRF-1s, tRF-3s, tRF-5s, and internal tRFs (i-tRFs or tRF-2s), while tiRNAs are divided into 5′tiRNA and 3′tiRNA (Figure 2) [81].

The tRF-1, also known as 3′U-tRF, originates from the 3′ untranslated regions (UTR) of pre-tRNA through RNase Z digestion, with the characteristic of a poly-U sequence [82]. The tRF-5s are generated from cleavage in the D-loop or the arm region between the D-loop and anticodon loop of mature tRNA, and include the intact sequence of the 5′ end of mature tRNA [83]. The tRF-3s originate from cleavage in the T-loop, and end with trinucleotides “CCA” [84]. The i-tRFs derive from an internal region of mature tRNA, and include the anticodon loop and part of the D- and T-loops [85] (Figure 2). Most human i-tRFs are 20 or 36 nucleotides long [86]. The tRF-5s occur predominantly in the nucleus; large numbers of tRF-5s are present in HeLa cell nucleoli [83]. tRF-3s and tRF-1 are more abundant in the whole cell fraction than the nuclear fraction, suggesting that both species occur exclusively in the cytoplasm [87]. Each class of tRFs is generated by specific ribonucleases and regulated by specific pathways.

One of the earliest discovered classes of tsRNAs were the stress (and starvation) induced tRNA fragments called tiRNA (tiR), or tRNA halves [88]. In mammals, tiRNAs are generated through cleavage by ribonuclease (RNAse (A or T)) angiogenin (ANG) within the anticodon loops of mature tRNAs [89]. This ANG cleavage produces two types of tiRNA: a tiRNA-5 and a tiRNA-3, which are the 5′ and 3′ half of mature tRNA, respectively [89] (Figure 2). The production of tiRNAs is induced by stress, such as starvation, oxidative stress, heat shock, UV irradiation, or viral infection [88,90]. The upregulation of ANG under certain conditions positively correlates with increased tiRNA levels [91,92,93]. RNH1, an ANG inhibitor interacting with ANG in the cytoplasm, is a negative regulator of tiRNA generation [90]. Several studies have shown that methylation of mature tRNA by DNA methyltransferase DNMT2 or cytosine-5 methyltransferase NSun2 enhances tRNA resistance to ANG cleavage [94,95].

**Figure 3:**
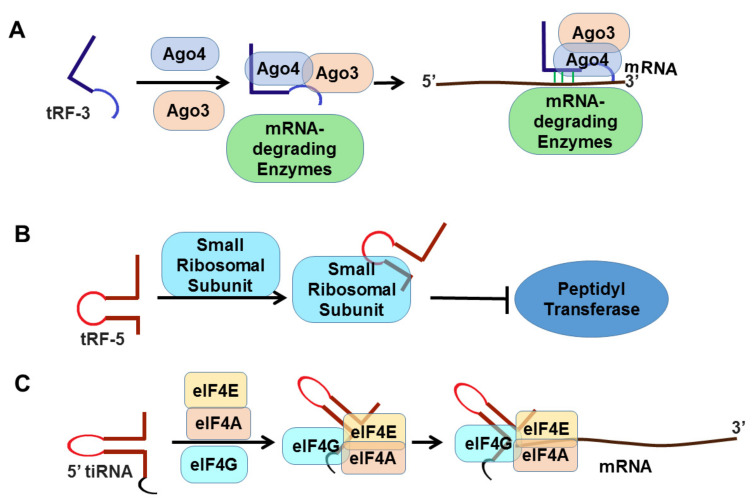
tRF- and tiRNA-mediated gene regulation: (**A**) Regulation of mRNA stability: The combination of tRF-3 with argonaute 3 (Ago3) and Ago4 binding to mRNA allows mRNA-degrading enzymes to degrade the target mRNA [101]; tRFs function like miRNAs to inhibit cancer-associated gene expression. Binding with the 3′ untranslated region (3′UTR) of target mRNA, Argonaute (Ago) protein and other proteins form an RNA-induced silencing complex (RISC). (**B**,**C**) Inhibition of translation: By binding to small ribosomal subunits, tRF inhibits peptidyl transferase activity that results in reduced protein abundance of the target gene [99,102]. 5′tiRNA inhibits translation by forming a RNA G-quadruplex (RG4s) that replaces the translation initiation complex eIF4G/eIF4E on the mRNA cap [103,104].

Both tRFs and tiRNAs play important roles in tumorigenesis [85,86,87,96,97], and are promising diagnostic biomarkers and therapeutic targets for cancer. tRFs can modulate protein translation and interact with ribosomes and aminoacyl tRNA synthetases [98,99]. In addition, tRFs can associate with Ago and PIWI proteins in a cell-type specific manner, potentially affecting gene expression (Figure 3). Moreover, the interaction between RNA-binding proteins and tRFs has been linked with cancer development and metastasis [85,100].

In cancer models, tsRNAs promote cell proliferation and cell cycle progression by regulating the expression of oncogenes and proto-oncogenes. Functional studies show that tRFs and tiRNAs may bind to RNA binding proteins such as Y-box binding protein 1 (YBX1) and prevent transcription, inactivate initiation factor eIF4G/A, promote translation of ribosomal proteins, activate aurora kinase A (the regulator of mitosis) by binding to cytochrome C, or promote the assembly of stress granules that helps cells survive under adverse conditions [105,106,107,108]. A recent study indicated that two specific tRFs derived from tRNA^Lys-CTT^ and tRNA^Phe-GAA^ are good indicators of progression free survival (PFS), and thus are candidate prognostic markers in prostate cancer [106].

#### 2.2.1. tRNA Derived Small RNAs in Gynecologic Cancer

A significant impact of the deregulated tsRNAs has been demonstrated in various malignancies, including gynecological cancers; these biological functions of tRFs are Ago-dependent. In ovarian cancer tRF5^Glu^ regulates breast cancer anti-estrogen resistance 3 (BCAR3) mRNA levels by direct binding to the 3′ untranslated region (UTR) [109]. In colon, breast, and ovarian cancer patients, as well as corresponding cell lines, the expression level of ts-101 and ts-46 (tRF-1s) correlates with chromatin structure, cell survival, cell proliferation, clonal growth, and apoptosis. The expression of tRFs also correlates with oncogene activation and ovarian cancer progression [110]. Reanalysis of existing RNA-sequencing data, from 180 serum samples, including 15 healthy controls, 46 benign tumors, 22 borderline tumors, and 97 ovarian cancer patients, revealed that tsRNAs cover a high proportion of total small RNA, and are non-random degradation products in serum (ranging from 2.5–29.4%), and which are enriched in several specific types of related tRNA (e.g., Gly-tRNA), and can predict abnormal cell proliferation with high accuracy [111]. Another group using serum samples from ovarian cancer patients and healthy donors, along with ovarian cancer cell lines, have shown differential expression of tRF; they showed that tRF-03357 promoted SK-OV-3 cell proliferation, migration, and invasion, as well as downregulating HMBOX1 [112]. In cervical cancer, preliminary studies using biopsy samples demonstrated that the expression of 5S rRNA, tRNA^Arg^, and tRNA^Sec^ was significantly elevated in the HPV16-containing samples when compared to the HPV-negative biopsies [113]. In Hela cells, 5′ tRFs derived from tRNA^Gln^ are produced in abundance [83]. Currently, tRNA/tiRNA status in endometrial cancer remains unexplored.

#### 2.2.2. Future Perspectives

tsRNAs are unique sequences derived from tRNA precursors, and generated in the nucleus. tsRNAs are frequently dysregulated in various cancers, including the gynecologic malignancies. Since tsRNAs can accompany both Ago proteins (like miR) and PIWI proteins (like piRNAs), they can regulate gene expression both pre-transcriptionally (like piRNA) and post-transcriptionally (like miR). Like piRNAs, tsRNAs are produced as single-stranded molecules, and can interact with DNA and the histone methylation machinery, suggesting a role in the pre-transcriptional regulation of gene expression. Like miRNAs, ts-53 (previously known as miR-3676) interacts with the 3′UTR of TCL1, supporting a role for tsRNAs in the posttranscriptional regulation of gene expression [81,114].

Rapidly proliferating tumor cells often overcome a deficient blood supply, resulting in a microenvironment with limited oxygen and nutrients. Tumor cells adapt to this stress with varying strategies, thus ensuring survival and proliferation [115]. Generation of tsRNA from tRNAs under stress is an important pathway, and the biological function of tsRNA mainly supports cell survival under stress. Additionally, tsRNAs can be detected in the urine and serum from cancer patients [111,116,117,118], suggesting their potential as molecular diagnostic markers. For example, high-throughput RNA sequencing in breast cancer patients showed that tsRNA blood levels closely relate to the pathological characteristics [119]; similarly, in breast cancer and prostate cancer, hormone-dependent tsRNA are expressed in abundance and enhance the proliferation of cancer cells [91]. Thus, a tsRNA database of different tumors may represent a new diagnostic tool for cancer management [114,120]. In summary, tsRNAs are implicated in tumor onset, progression, and drug response, and thus represent potential therapeutic targets and/or diagnostic markers.

### 2.3. Micro-Ribonucleic Acid (miR)

Micro-ribonucleic acids (miRs/micro-RNAs) are short (18–25 nucleotides), evolutionarily conserved, and endogenously expressed regulatory RNA molecules, which belong to the family of ncRNAs. The miRs were first detected in the early 1990s in *Caenorhabditis elegans* [121], and later studies confirmed their presence in almost all species [122,123]. Although most miRs modestly alter expression of their target genes, the intricate network of miR target genes and downstream effectors plays a profound role in the regulation of biological pathways [124,125,126].

Among the non-coding RNAs, miR are the most widely studied, and apart from classical 3′ mRNA targeting and cytosolic expression, miR are also known for their nuclear expression [127]; the biogenesis and mRNA regulatory functions of miR have been extensively reviewed by Jacob O’Brien and others [30,31,128,129]. In brief, miRs are synthesized in the nucleus by DNA polymerase II [130] as a long double-stranded precursor called pri-miR. This pri-miR is cleaved at specific sites by the RNAse drosha inside the nucleus, producing a precursor miR (pre-miR) [131]. The pre-miR is exported to the cytoplasm by the exportin 5 protein, where it is processed by dicer into mature miR. Mature miRs are then activated through binding to the RNA-induced silencing complex (RISC); via the RISC, miRs can regulate their target mRNAs, leading to translational repression or degradation. The sequence at the 5′ end of the mature miR is called the “seed region”, or “seed sequence”. The seed sequence of the miR binds the complementary sequence within the 3′ untranslated region (3′ UTR) of target mRNAs [131,132]. Some miRs also interact with the 5′ UTR [133], coding sequence [134], and promoter regions of their targets [135]. Perfect or near-perfect complementarity between the miR and its mRNA target results in mRNA degradation, while imperfect complementarity leads to translational inhibition [136]. Several parameters, including the subcellular location of the miR, the quantity of both miR and its target mRNA, and the affinity, modulate the miR–mRNA interaction [128].

miRs are the critical modulators that regulate gene expression at the post-transcriptional level; they govern the stability and translation of protein coding mRNAs and are thus involved in almost every biological process, including regulation of cell division, differentiation, growth, and apoptosis. Dysregulation of miRs is instrumental in various pathophysiologies including neurodegenerative, inflammatory, metabolic, and cardiovascular diseases and, significantly for this review, cancer [137,138]. Altered expression of hundreds of miRs is closely associated with tumor development, invasion, metastasis, and drug resistance in cancer [139,140]. The miR expression profile differs significantly between normal and cancerous tissues, localized and aggressive cancer, and across type and stages of cancer.

Differential miR expression in normal and tumor cells has been reported in numerous studies, and suggests a robust association of altered miR expression with cancer pathogenesis and progression [141,142]. More than half of all annotated human miR genes are located in cancer-associated genomic regions that are amplified, deleted, or translocated in cancer [143]. Mechanistically, miRs regulate tumor suppressor genes and oncogenes; based on the regulated gene, miRs can be either oncogenic or tumor suppressive. The altered expression of miRs that control tumor suppressor genes and oncogenes results in cancer. Dysregulated miR expression has several causes, including genetic alteration, epigenetic changes, and SNPs in miR coding genes, as well as defects in factors regulating miR biogenesis.

#### 2.3.1. miR in Gynecologic Cancer

miRs are estimated to control over 50% of the activities of all protein coding genes [144], and are involved in regulation of almost all cellular processes [145]. Multiple studies have shown that dysregulation of miRs leads to a variety of human diseases, including cancer [146]. The relationship between miR expression and gynecologic cancer is well documented [147,148,149,150], but the significance of the cumulative effect of miR expression may not have been fully realized as each miR targets multiple genes associated with various cellular processes. Herein we summarize the key research findings on the significance of miR in gynecological cancer progression, prognosis, and diagnosis. We also present a compressive analysis of the miR expression in ovarian, endometrial, and cervical cancer, grouping them based on their expression. These miR are further analyzed using a systems biology approach for target prediction, and these target proteins are then analyzed for associated pathways to find the significance of altered miR in respective gynecological cancers. Based on the number of predicted target genes and associated pathways, miR can be best prioritized for possible disease interventions. A text-mining program called IRIDESCENT [151] was used to document details of the different miRs associated with ovarian, endometrial, and cervical cancer. IRIDESCENT identified the co-occurrences of miRNAs and gynecological cancer within MEDLINE titles and abstracts by analyzing public databases (e.g., Entrez, OMIM, and Disease Ontology) for a thesaurus of names and synonyms of miRNAs and diseases. The strength of association between gynecologic cancer and miR was calculated by the frequency of co-mention, (+0.5 for every abstract, +0.8 for every sentence). Associations and directionality, i.e., up- or downregulation, was confirmed manually for the respective types of cancer. Deregulated miRs were further grouped based on their expression in respective cancers, and potential significance was analyzed using miRNET [152].

##### Role of miRs in Ovarian Cancer Pathogenesis

Ovarian cancer is characterized by wide-scale deregulation of miRs, and aberrant expression of miRs in ovarian cancer is known to correlate with histotype, lymphovascular, and organ invasion [153]. We have reported that miR-15a and miR-16 are under-expressed in ovarian cancer tumor samples compared to normal tissue, and that ectopic expression of these miRs significantly inhibits ovarian cancer progression in preclinical tumor models [154,155]. Additionally, we have demonstrated that miR-195 is under-expressed in ovarian cancer and regulates ovarian cancer progression by regulating the expression of Mitochondrial Calcium Uptake 1 (MICU1) [156]. miR have been shown to play a key role in all the stages of tumorigenesis, and are also significant for diagnosis, prognosis, and therapeutics. Differential miR expression has been shown to be associated with ovarian cancer progression, omental metastasis, and drug resistance. Compared to normal ovarian tissue, miR-200a, miR-141, miR-200c, and miR-200b were significantly upregulated, whereas miR-199a, miR-140, miR-145, and miR-125b1 were among the most downregulated miRNAs in ovarian tumor tissues [153]. In another study miR-146a and miR-150 were reported to be significantly associated with omental metastasis and cisplatin resistance, and were shown to induce spheroid formation [157]. miR-200 and miR-429 are associated with recurrence and survival rates of ovarian cancer; their increased expression inhibits cancer metastasis [158].

The diagnosis of ovarian cancer has always been a significant problem; approximately 70–80% of patients present with advanced cancer at diagnosis. However, the results of miRNA research suggest that miR may be helpful in the early detection of ovarian cancer. Differences between the miR profiles of ovarian surface epithelium (OSE) and ovarian cancer, and the potential role of miRs in ovarian cancer diagnosis have been assessed in several studies [153,159,160,161,162,163,164,165,166,167]. In various reports miR-205, miR-429, miR-141, miR-200c, miR-93, miR-16, miR-20a, miR-21, miR-27a, miR-200a, miR-200b, and miR-200c [158,165,168] have been shown to be upregulated, while miR-320c, miR-383, let-7b, miR-99a, miR-125b, miR-145, miR-100, miR-31, miR-137, miR-132, and miR-26a were downregulated [163,165,167] in ovarian serous carcinoma samples. Other studies in ovarian cancer highlighted that miR levels can also discriminate between malignant and benign tumor, i.e., the expression pattern of let-7i-5p, miR-152, miR-122-5p, and miR-25-3p were significantly downregulated in malignant tumors compared to benign samples [160].

Presently, miRs are being evaluated for their potential utility as therapy candidates. In our previous reports, we have shown that the nanoliposomal delivery of miR-15a and miR-16 in combination, in a pre-clinical chemo-resistant orthotopic mouse model of ovarian cancer, demonstrated a striking reduction in tumor burden compared to cisplatin alone [154], while the importance of miR in cancer therapy has been reviewed previously [169,170].

**Figure 4:**
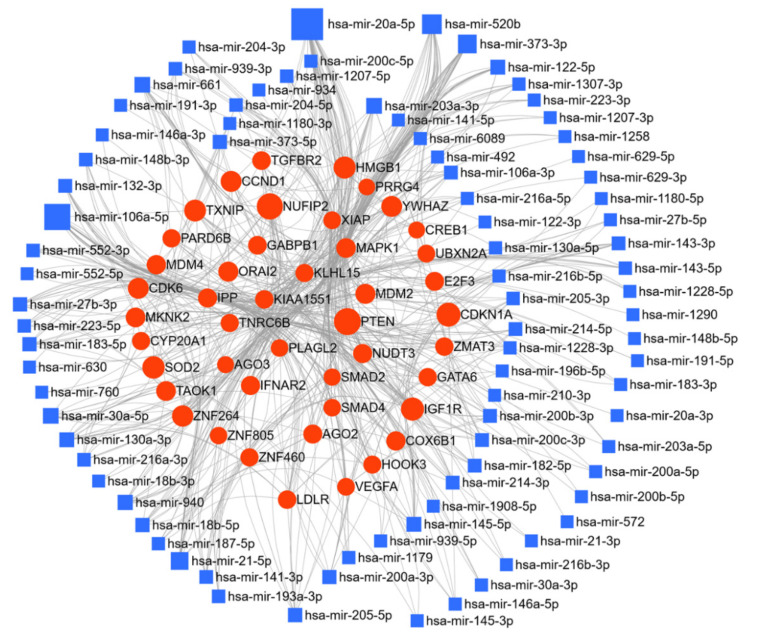
Network of upregulated miR in ovarian cancer: miR deregulated in ovarian cancer were searched for in the PubMed database using IRIDESCENT. The network of upregulated miR generated using miRNET (degree filter cutoff 10) is shown. The blue squares represent miR, and the red dots represent target genes of these miRs.

**Figure 5:**
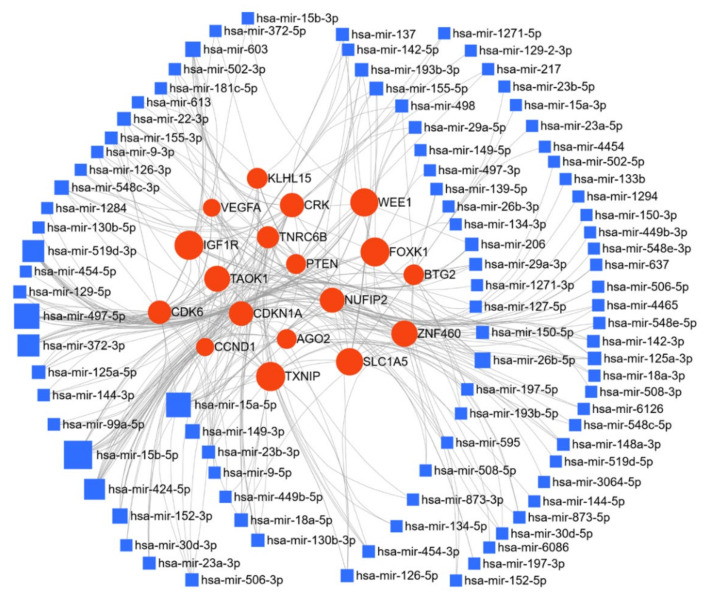
Network of downregulated miR in ovarian cancer: miR deregulated in ovarian cancer were searched for in the PubMed database using IRIDESCENT. The network of downregulated miR generated using miRNET (degree filter cutoff 15) is shown. The blue squares represent miR, and the red dots represent target genes of these miRs.

Here, we have listed micro-RNAs deregulated in ovarian cancer using the text-mining program IRIDESCENT. The deregulated miRs were manually grouped by their expression levels compared to the control group (Table 1). Fifty-three miRs were reported to be upregulated, and sixty-eight miRs were downregulated in ovarian cancer. The miRNet web tool (https://www.mirnet.ca/ accessed between December 2020 and January 2021) was used for microRNA-gene target prediction (using miRTarBase v8.0 [171], and the parameters of degree and betweenness were used to derive the network, setting the cutoff for the degree filter at five. The network was further used to elucidate the biological processes and pathways of these upregulated, and downregulated targets, and networks are presented in Figure 4 and Figure 5. In the ovarian cancer upregulated miR group, a total of 7605 gene targets were found; of special note miR-20a-5p regulates 14.1% and miR 106a-5p regulates 9.4% of target genes. Kyoto Encyclopedia of Genes and Genomes (KEGG) pathway analysis enrichment was performed for these miR targets genes (degree cutoff 5); which revealed 24 significantly enriched pathways (cutoff *p* > 0.001), these altered pathways are known for their significant role in cancer progression, (Table 2). Gene ontology enrichment for biological process (GO-BP) and molecular function (GO-MF) were also analyzed; using *p* > 0.001 as the cutoff, GO-BP and GO-MF respectively identified 67 and 24 pathways as enriched, and the top 10 pathways are shown in Table 2.

Similar analyses for the downregulated miRs in ovarian cancer (Figure 2) identified 9287 gene targets. miR-26b-5p, miR-519d, miR-15a, and miR-15b regulated 20.8%, 11.3%, 8.6%, and 8.9% of the target genes, respectively. KEGG, GO-BP, and GO-MF enrichment analysis of the targets respectively identified 41, 95, and 38 enriched pathways. The top 10 pathways for each analysis are shown in Table 2.

While mir-21 and mir-155 are the most studied miRs in ovarian cancer, our analysis suggests that the genes targeted by miR-20a-5p and miR-26b-5p may play a major role in the progression of ovarian cancer (Figure 2). The most prominent targets of these miRs are phosphatase and tensin homolog (PTEN), cyclin dependent kinase inhibitor 1A (CDKN1A), MDM2 proto-oncogene (MDM2), superoxide dismutase 2 (SOD2), high mobility group box 1 (HMGB1), insulin like growth factor 1 receptor (IGF1R), WEE1 G2 checkpoint kinase (WEE1), forkhead box K1 (FOXK1), and thioredoxin interacting protein (TXNIP), which play a crucial role in ovarian cancer progression. From the pathway enrichment analysis, these deregulated miRs were shown to be involved in essential cellular processes and key signaling pathways, i.e., cell proliferation, regulation of cell cycle and apoptosis, adherens junction, focal adhesion, regulation of actin cytoskeleton, regulation of transcription, p53 signaling pathway, TGF-beta signaling pathway, Erbb signaling pathway, neurotrophin signaling pathway, Wnt signaling pathway, Jak-STAT signaling pathway, and insulin signaling pathways (Table 2). Our systemic analysis of these deregulated miRs, their target genes, and the target pathways, which are instrumental in cancer progression, reinforced the importance of these miRs in ovarian cancer, and may help prioritize miRs as targets for therapeutic evaluation.

##### Role of miRs in Endometrial Cancer Pathogenesis

The association of miR expression with the prognosis of endometrial cancer including lymph node metastasis, lymphovascular space invasion, overall survival, and recurrence-free survival is well documented [299,300,301,302,303,304]. Srivastava et al. [305] reviewed miRs differentially expressed between endometrial cancer and normal endometrial tissue, including upregulation of miR-9, miR-92a, miR-141, miR-182, miR-183, miR-186, miR-200a, miR-205a, miR-222, miR-223, miR-410, miR-429, miR-449, and miR-1228, and the downregulation of miR-99b, miR-143, miR-145, miR-193b, and miR-204. Numerous miRs regulate endometrial cancer cell proliferation by silencing their target genes [306,307], and miR expression profiles associate with stage, grade, relapse, and nodal metastases in endometrial cancer [301,305,308]; their importance has been reviewed [148].

**Figure 6:**
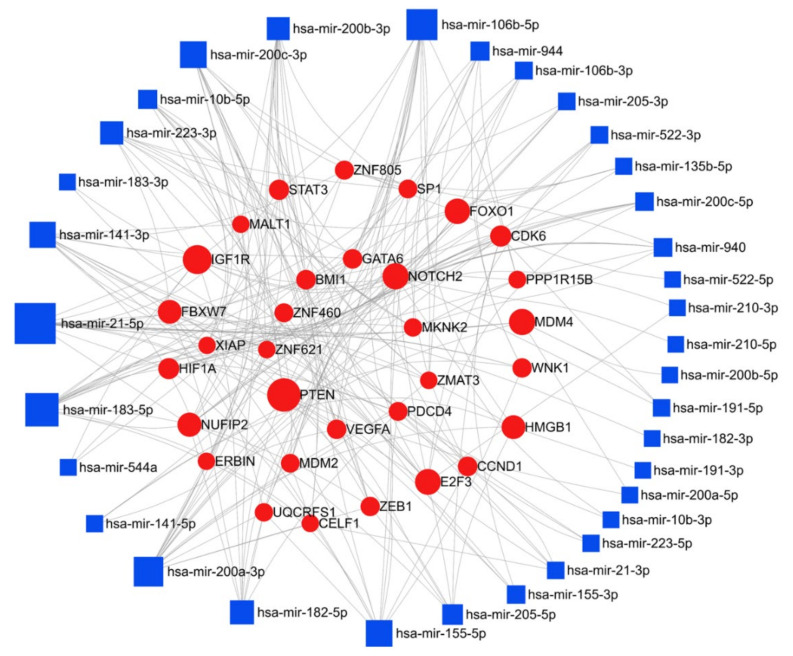
Network of upregulated miR in endometrial cancer: miR deregulated in endometrial cancer were searched in the PubMed database using IRIDESCENT. The network of upregulated miR generated using miRNET (degree filter 5) is shown. The blue squares represent miR, and the red dots represent target genes of these miRs.

**Figure 7:**
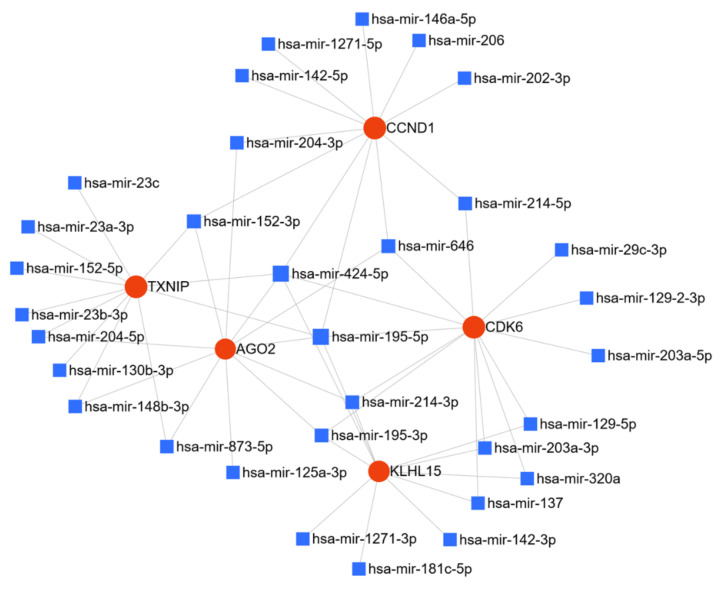
Network of downregulated miR in endometrial cancer: miR deregulated in endometrial cancer were searched in the PubMed database using IRIDESCENT. The network of downregulated miR generated using miRNET (degree filter cutoff 10) is shown. The blue squares represent miR, and the red dots represent target genes of these miRs.

Here, we have compiled a list of micro-RNAs that are deregulated in endometrial cancer using the text-mining program IRIDESCENT (Table 3). We report nineteen upregulated and twenty-seven downregulated miRs in endometrial cancer. The miRNet web tool (https://www.mirnet.ca/) was used for microRNA-gene target prediction, and the parameters of degree and betweenness were used to draw the network, and were further used to elucidate the predicted biological processes and pathways of these upregulated, and downregulated targets; the networks are presented in Figure 6 and Figure 7. In endometrial cancer, upregulated miR were predicted to target a total of 4224 genes. Among these target genes, 14.5% and ~26% are targeted by miR-21-5p and miR-106a-5p, respectively. Pathway enrichment analysis was performed on the target genes using KEGG, GO-BP, and GO-MF, as described above, and these methods respectively identified 32, 96, and 34 enriched pathways. A similar analysis was performed using the downregulated miRs; 5749 gene targets were identified, a network was generated with degree filter 5, and miR-195-5p and miR-424-3p respectively regulated 11.1% and 8.2% of the target genes. KEGG, GO-BP, and GO-MF enrichment analysis respectively identified 57, 100, and 97 pathways. The top 10 KEGG pathways, biological processes, and the molecular functions altered by the miRs are shown in Table 4. A literature search showed miR-205 to be the most studied miR in endometrial cancer, however, our target analysis showed miR-21-5p and miR-106-5p to be the most interconnected miRs that can regulate multiple targets responsible for endometrial cancer progression. The most prominent targets of these miRs are PTEN, IGF1R, Notch Receptor 2 (NOTCH2), cyclin D1 (CCND1), TXNIP, AGO2, and cyclin dependent kinase 6 (CDK6). The pathway enrichment analysis showed that these deregulated miRs are involved in critical cellular processes and key signaling pathways, i.e., cell cycle, proliferation, programmed cell death, cellular response to stress, p53 signaling pathway, mTOR signaling pathway, insulin signaling pathway, notch signaling pathway, NOD-like receptor signaling pathway, inositol phosphate metabolism, and adipocytokine signaling pathway (Table 4), all of which support the importance of these miRs in the progression of endometrial cancer.

##### Role of miRs in Cervical Cancer Pathogenesis

miR deregulation plays a key role in the malignant transformation of cervical cancer, and, along with their target genes, miRs have been exploited for both prognostic and therapeutic strategies in cervical cancer. HPV causes about 91% of cervical cancers [354], and the high-risk HPV strains HPV-16 and HPV-18 in keratinocyte cell lines have been shown to regulate expression of different miRs [237]. The study reports that both HPV strains induced the expression of miR-16, miR-25, miR-92a, and miR-378, while they inhibited the expression of miR-22, miR-27a, miR-29a, and miR-100. Moreover, data from cervical-tissue specimens correlated with in vitro results; miR-25, miR-92a, and miR-378 were increased with cervical cancer progression, which supports the significance of HPV mediated miR expression in cervical cancer pathogenesis [237].

Mounting evidence supports the significance of miR expression in cervical cancer, e.g., miR-21, which is reportedly overexpressed in various malignancies [355], promotes cell proliferation in HeLa cervical carcinoma cells by targeting programmed cell death 4 (PDCD4) expression. In addition, miR-20a [356], as well as miR-106a [357], suppressed the migration and invasion of cervical cancer cells by targeting TIMP2.

The potential of miR for early detection and as a prognostic biomarker in cervical cancer has also been explored. Expression profiles of circulating miRs in cervical cancer patient samples compared to healthy volunteers demonstrated that the expression of miR-20a and miR-203 was upregulated in the cervical cancer patients’ sera, and circulating levels of miR-20a could be useful for the detection of lymph-node metastasis [358]. Various reports have shown the correlation of miR expression with cervical cancer stage; miR-494 is upregulated, while the expression of miR-195 and miR-144 was downregulated in patients with stage IB cervical carcinoma [359,360,361]. In Stages I and II, the levels of miR-375, miR-145, and miR-124 were downregulated, while miR-99a/b, miR-92a, miR-150, and miR-21 levels were upregulated [359,362,363,364,365,366,367,368]. In cervical cancer patients, levels of miR-218 were similarly decreased in plasma and tumor samples, and levels correlated with tumor stage [369]. Differentially expressed miRs in the serum of cervical cancer patients, compared with controls, measured using Solexa sequencing, identified 12 miRs of interest. Five were upregulated, i.e., miR-21, miR-29a, miR-200a, miR-25, and miR-485-5p, and likely are credible alternatives to current tumor markers, such as squamous cell carcinoma (SCC) antigen and carbohydrate antigen 125 (CA125) [370]. Elevated serum levels of miR-205 correlate with both cervical cancer tumor stage and decreased survival rates. Some of the aforementioned miRs may be viable diagnostic and prognostic biomarkers for cervical cancer progression. Circulating levels of miR-646, miR-141, and miR-542-3p are significantly different when assayed before and after surgical procedures [371], and may be useful in monitoring the health status of patients after treatment.

The role of miRs in cervical cancer progression [372,373,374,375,376], therapeutics [150,377], and drug resistance is well documented [378]. The association of miRs with HPV infection is also well established, and includes cellular miR-9 [379], cellular miR-21 [41,51,148], exosomal vesicles (EV)-derived miR-21 [380,381], cellular miR-34a [376,381], EV-derived miR-34a [381], cellular miR-146a [382], and EV-derived miR-146a [380]. 

**Figure 8:**
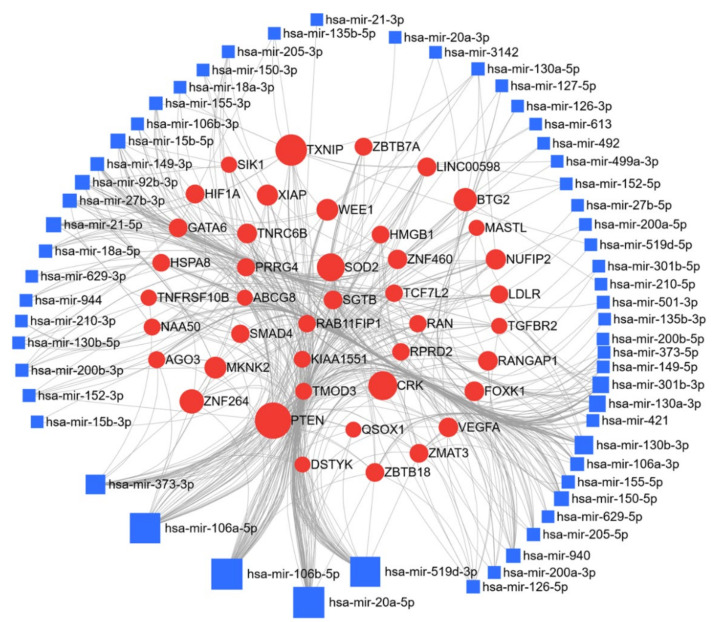
Network of upregulated miR in cervical cancer: miRs deregulated in cervical cancer were searched in the PubMed database using IRIDESCENT. The network of upregulated miRs generated using miRNET (degree filter cutoff 10) is shown. The blue squares represent miR, and the red dots represent target genes of these miRs.

**Figure 9:**
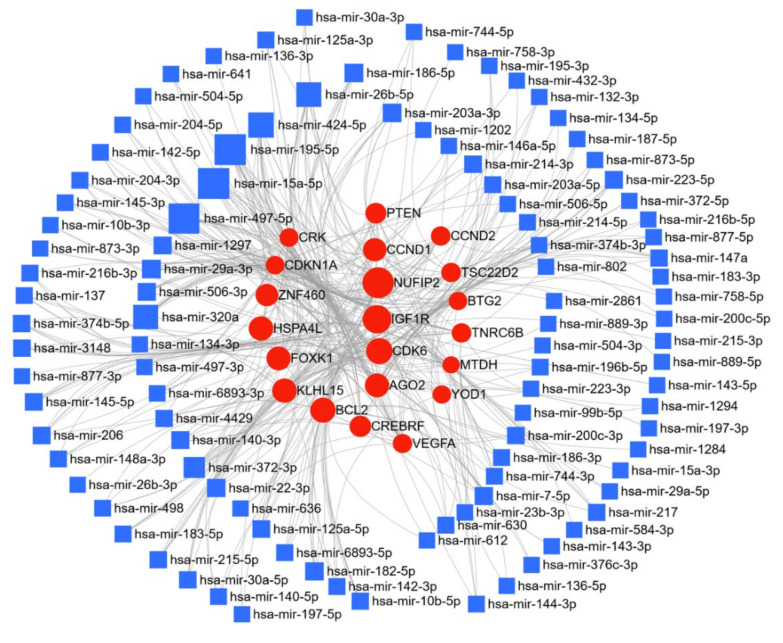
Network of downregulated miR in cervical cancer: miR deregulated in cervical cancer were searched in the PubMed database using IRIDESCENT. The network of downregulated miR generated using miRNET (degree filter 10) is shown. The blue squares represent miR, and the red dots represent target genes of these miRs.

Here, we have compiled a list of micro-RNAs deregulated in cervical cancer (Table 5); thirty-six miRs were reported to be upregulated and seventy downregulated in cervical cancer. The miRNet web tool, as discussed above, was used for target gene prediction, and parameters of degree and betweenness were used to draw the network, and were further used to elucidate the predicted biological processes and pathways of the upregulated, and downregulated targets. The miR and the target protein networks are shown in Figure 8 and Figure 9. In cervical cancer, target prediction using upregulated miR resulted in 6957 gene targets; mir-106b-and miR-106a respectively regulate 15.7% and 10.3% of the target genes. Networks for these targets were generated and pathway enrichment analysis performed using KEGG, GO-BP, and GO-MF parameters, as before; they identified 19, 94, and 10 enriched pathways, respectively (Table 6). A similar analysis with the downregulated miR, generated 9715 gene targets, of which miR15a-5p targeted 7.4%, and miR195-5p regulated 6.6%. KEGG, GO-BP, and GO-MF enrichment analyses were applied and respectively identified 51, 96, and 55 pathways (Table 6). From our collective analysis of miR and their target genes in cervical cancer based on the number of target genes and associated networks, the most important upregulated miRs were miR-106, miR-20a, and miR-519d, while the most important downregulated were miR-15a and miR-195. The most prominent targets of these miRs are PTEN, CRK proto-oncogene, adaptor protein (CRK), SOD2, TXNIP IGF1R, BCL2 apoptosis regulator (BCL2), and FOXK1, which play a crucial role in cervical cancer progression. Based on the network analysis these deregulated miRs are involved in important cellular pathways and key signaling processes, i.e., cell cycle, adherens junction, pyrimidine metabolism, focal adhesion, cell proliferation, nuclear transport; and the p53 signaling pathway, Wnt signaling pathway, PPAR signaling pathway, G1 phase of mitotic cell cycle, ErbB signaling pathway, insulin signaling pathway, and MAPK signaling pathway (Table 6). All these cellular processes and signaling pathways are instrumental in the progression of cervical cancer, and further substantiate the significance of these miRs in cervical cancer.

#### 2.3.2. Future Perspectives

Ongoing research reveals the diagnostic potential and therapeutic promise of miRs for multiple cancers, including those of the female reproductive system. However, their diagnostic and/or therapeutic translation in terms of helping patients in need has not been fully apprehended, and before this becomes a reality, our understanding of the diverse roles of miRs must become complete. There is a certain level of redundancy in gene regulation by miRs, as a single miR can target multiple genes, and a single gene can be the target for multiple miRs. Thus, any miR selected as a therapeutic target must be comprehensively investigated, all target genes must be identified, and the outcome(s) of their combined inhibition should be carefully evaluated. Conflicting reports on the functionality of a single miR within a specific gynecologic cancer, as well as across different gynecologic cancers, suggest that we do not fully understand the complex nature of miR-mediated regulation, which may be tissue and cell-context specific. As an example, miR-26a was first shown to promote ovarian cancer proliferation through its suppression of ER-α [481], while subsequently, it was shown to inhibit proliferation of ovarian cancer cells through its regulation of CDC6 [482]. In a cervical cancer model, miR-26a inhibited cell proliferation, migration, and invasion in vitro, and also inhibited tumor growth in vivo in a xenograft model [483]. Similarly, miR-31 [484,485,486], miR-214 [181,487], miR-494 [361,488,489], and miR-222 [490,491] have different reported roles in different gynecologic cancers. This conflict arises from the targeting of multiple genes by the same miR, and the effects must be evaluated at a systems level using systems biology approaches in order to translate miRs into successful cancer therapies. Enrichment analysis based on the validated/predicted targets, as we have shown herein, will undoubtedly help in selecting the best miRs for a specific diagnostic, therapeutic, or predictive purpose. Concurrent with efforts to overcome the inherent barriers to miR use, strategies to manipulate miR levels in cancer patients should be developed further. This will permit the rapid translation of constructive findings to the clinic for the benefit of millions of gynecologic cancer patients worldwide.

## 3. Conclusions

We remain in the early stages of decoding the essential role of the small noncoding RNAs which were previously believed to be transcriptional debris. It is now well established that these small non-coding RNAs play a key role in gene regulation, and have a profound role in the development of multiple cancers, including gynecologic malignancies. Our understanding of the roles of piRNA- and tRNA-derived small RNAs is still very limited; interestingly, much valuable information is available for miR, and a few studies are at the preliminary stages of development for cancer therapy, with some clinical trials in progress for ovarian cancer (ClinicalTrials.gov Identifier: NCT03738319, NCT03776630, NCT01970696, NCT02253251, NCT01391351, NCT03877796), endometrial cancer (ClinicalTrials.gov Identifier: NCT02983279, NCT01119573, NCT03824613), and cervical cancer (ClinicalTrials.gov Identifier: NCT04087785). Based on this accumulating evidence, it is possible that in the near future miRs in plasma may be used for the diagnosis and prognosis of various cancers. One limitation of these miR expression data may be the lack of evaluation among diverse ethnic groups; however, as more information about the diverse types of small non-coding RNA and their target networks become available, it will assist us in refining their possible diagnostic and therapeutic applications. The primary causes of greater cancer related mortality, such as in ovarian cancer, are late diagnosis and acquired resistance; advancing the use of small noncoding RNA in cancer diagnosis and therapy will enhance early detection and therapeutic intervention, and will certainly be a significant step forward in the management of gynecologic malignancies.

## Figures and Tables

**Table 1:** Deregulated miR in Ovarian Cancer.

Related Entity	Lit Str.	Lit MIM	Regulation	No. of Papers	Ref
miR-21	84.4	−1.9	Up	66	[172,173,174,175,176]
miR-200C	69.5	−0.1	Up	57	[177,178]
miR-145	75.2	−1	Up	48	[153,179,180]
miR-200A	52	0.2	Up	43	[181]
miR-200B	28.4	−0.1	Up	35	[174,179,182]
miR-141	30.1	−0.3	Up	33	[178,179,183]
miR-214	42.4	−0.6	Up	31	[181]
miR-205	52.3	−0.5	Up	30	[178,179,180,184]
miR-182	35.1	−0.6	Up	24	[184,185]
miR-146A	20.8	−2	Up	21	[186]
miR-210	13.5	−1.6	Up	15	[187]
miR-130A	23.6	−0.4	Up	14	[188]
miR-143	3.9	−2.8	Up	12	[163]
miR-30A	4.9	−1.6	Up	11	[189]
miR-106A	20.3	−0.9	Up	10	[190]
miR-204	4.9	−1.8	Up	10	[191]
miR-132	18.9	−1.5	Up	9	[163,192,193]
miR-20A	7.6	−2	Up	8	[194]
miR-183	5.2	−1.8	Up	7	[195]
miR-630	10.2	0.1	Up	6	[196]
miR-223	9.6	−1.9	Up	6	[197]
miR-216A	8.7	−1	Up	6	[198]
miR-373	4.4	−0.9	Up	6	[199]
miR-203A	39	−1.2	Up	5	[179,200]
miR-193A	7.6	−0.5	Up	5	[201]
miR-191	4.1	−0.7	Up	5	[202]
miR-1307	18	1.3	Up	4	[203]
miR-27B	9.2	−1.6	Up	4	[204]
miR-187	6	−0.3	Up	4	[205]
miR-492	8.7	0.1	Up	3	[206]
miR-760	8.2	−0.8	Up	3	[207]
miR-661	7.9	0.3	Up	3	[208]
miR-1181	7.1	1.2	Up	3	[209]
miR-1258	7.1	0.2	Up	3	[210]
miR-940	6.6	−1	Up	3	[211]
miR-1290	3.9	−0.7	Up	3	[212]
miR-148B	3.7	−2.2	Up	3	[213]
miR-939	3.7	−1	Up	3	[214]
miR-572	8.2	0.2	Up	2	[215]
miR-1180	7.4	0.6	Up	2	[216]
miR-18B	7.4	−0.6	Up	2	[217]
miR-1908	5.8	0	Up	2	[218]
miR-1228	4.5	0.1	Up	2	[219]
miR-122	4.2	−3.7	Up	2	[220]
miR-196B	4.2	−1.4	Up	2	[221]
miR-552	7.7	−0.7	Up	1	[222]
miR-1207	5.3	1.1	Up	1	[223]
miR-629	5.3	−0.6	Up	1	[224]
miR-520B	4.5	−1.3	Up	1	[225]
miR-934	4.2	1	Up	1	[226]
miR-1179	3.7	−0.5	Up	1	[227]
miR-216B	3.7	−1.9	Up	1	[228]
miR-6089	3.7	0.7	Up	1	[229]
miR-155	8.8	−3.1	Down	21	[230]
miR-137	38.5	−1	Down	16	[231,232,233]
miR-22	27.1	−1.1	Down	15	[234]
miR-506	15.9	0.1	Down	15	[235]
miR-23A	23.7	−1.2	Down	12	[236]
miR-206	21.1	−1.9	Down	12	[237]
miR-152	20	−0.7	Down	11	[238]
miR-30D	10.7	−0.2	Down	11	[239]
miR-148A	39.1	−0.8	Down	11	[240]
miR-130B	18.7	−0.7	Down	10	[241]
miR-126	11.3	−2.6	Down	10	[242]
miR-193B	10.8	−0.9	Down	9	[243]
miR-18A	20.9	−1.4	Down	8	[244]
miR-99A	10.8	−1.2	Down	8	[245]
miR-497	24.6	−1.1	Down	7	[246]
miR-23B	15.5	−1	Down	7	[247]
miR-29A	9	−2	Down	7	[248]
miR-125A	12.7	−1.8	Down	6	[249]
miR-134	11.3	−1.1	Down	6	[250]
miR-133B	23.3	−1.5	Down	5	[251]
miR-149	16.2	−2	Down	5	[252]
miR-26B	11.9	−1.8	Down	5	[253]
miR-150	7.3	−2	Down	5	[254,255]
miR-217	3.9	−1.7	Down	5	[256]
miR-144	17.7	−1.5	Down	4	[257]
miR-1271	16.7	−0.6	Down	4	[258]
miR-613	13.5	−1	Down	4	[259]
miR-498	12.4	−0.2	Down	4	[260]
miR-595	5.3	−0.3	Down	4	[261]
miR-424	4.7	−1.5	Down	4	[262]
miR-15A	3.9	−2.3	Down	4	[155]
miR-449B	3.9	0.5	Down	4	[263]
miR-139	12.2	−1.3	Down	3	[264]
miR-454	11.9	−0.4	Down	3	[152]
miR-15B	9	−2.1	Down	3	[265]
miR-181C	8.2	−1.6	Down	3	[266]
miR-136	7.1	−0.7	Down	3	[267]
miR-372	5.5	−0.7	Down	3	[268]
miR-1182	7.4	1.1	Down	2	[269]
miR-3064	7.4	1.6	Down	2	[270]
miR-519D	7.4	−0.6	Down	2	[271]
miR-LET7C	6.9	−2.6	Down	2	[272]
miR-508	6.6	1.1	Down	2	[273]
miR-598	5.8	0.1	Down	2	[274]
miR-6126	5.8	1.9	Down	2	[275]
miR-1294	5.3	−0.9	Down	2	[276]
miR-197	5.3	−1.7	Down	2	[277]
miR-1284	5	0.3	Down	2	[278]
miR-127	3.9	−0.8	Down	2	[279]
miR-637	3.7	−1	Down	2	[280]
miR-LET7B	10.6	−0.7	Down	1	[281]
miR-8073	9	1.6	Down	1	[282]
miR-LET7E	8.6	−0.1	Down	1	[283]
miR-LET7D	7.6	−0.6	Down	1	[284]
miR-9-1	6.3	0.5	Down	1	[285]
miR-603	6.1	−0.3	Down	1	[286]
miR-873	6.1	−1.3	Down	1	[287]
miR-LET7I	6	−0.5	Down	1	[189]
miR-548C	5.3	1.5	Down	1	[288]
miR-502	5	0.4	Down	1	[289,290]
miR-129-2	4.7	0.1	Down	1	[291]
miR-4454	4.5	0.5	Down	1	[292]
miR-548E	4.5	1.6	Down	1	[293]
miR-551A	4.5	0.5	Down	1	[294]
miR-4465	3.7	0.9	Down	1	[295]
miR-6086	3.7	1.2	Down	1	[296]
miR-634	3.7	−0.6	Down	1	[297]
miR-142	3.4	−1.5	Down	1	[298]

Deregulated miR in ovarian cancer. Lit Str. and Lit MIM represent: literature strength, and literature mutual information, respectively. The miR were regrouped based on their expression. The number of publications with the miR was retrieved from PubMed, and representative manuscripts are cited.

**Table 2:** Altered Pathways by Deregulated miR in Ovarian Cancer.

A. KEGG Pathways Regulated by Rpregulated miR				
S.No.	Name	Hits	*p*-Value	Adj. *p*-Value
1	Pathways in cancer	36	1.76 × 10^−12^	1.76 × 10^−10^
2	Chronic myeloid leukemia	18	3.93 × 10^−12^	1.97 × 10^−10^
3	Prostate cancer	18	9.44 × 10^−11^	3.15 × 10^−09^
4	p53 signaling pathway	16	1.44 × 10^−10^	3.60 × 10^−09^
5	Bladder cancer	11	4.30 × 10^−10^	8.60 × 10^−09^
6	Glioma	14	7.53 × 10^−09^	1.26 × 10^−07^
7	Pancreatic cancer	14	1.71 × 10^−08^	2.44 × 10^−07^
8	Cell cycle	18	3.73 × 10^−08^	4.66 × 10^−07^
9	Adherens junction	13	1.73 × 10^−07^	1.92 × 10^−06^
10	Melanoma	12	9.43 × 10^−07^	9.43 × 10^−06^
**B. Gene Ontology Enrichment for Biological Process (GO-BP) Regulated by Upregulated miR**				
**S.No.**	**Name**	**Hits**	***p*-Value**	**Adj. *p*-Value**
1	G1 phase of mitotic cell cycle	11	7.03 × 10^−08^	5.6 × 10^−06^
2	G1 phase	11	1.12 × 10^−07^	5.6 × 10^−06^
3	Gland development	27	2.20 × 10^−07^	5.98 × 10^−06^
4	Negative regulation of transcription from RNA polymerase II promoter	39	2.39 × 10^−07^	5.98 × 10^−06^
5	Regulation of cell proliferation	72	1.72 × 10^−06^	3.44 × 10^−05^
6	Negative regulation of transcription, DNA-dependent	54	4.07 × 10^−06^	4.67 × 10^−05^
7	Response to ionizing radiation	54	4.07 × 10^−06^	4.67 × 10^−05^
8	Negative regulation of cellular biosynthetic process	14	4.16 × 10^−06^	4.67 × 10^−05^
9	Negative regulation of RNA metabolic process	63	4.2 × 10^−06^	4.67 × 10^−05^
10	Negative regulation of nucleobase-containing compound metabolic process	55	5.68 × 10^−06^	5.45 × 10^−05^
**C. Gene Ontology Enrichment for Molecular Function (GO-MF) Regulated by Upregulated miR**				
**S. No.**	**Name**	**Hits**	***p*-Value**	**Adj. *p*-Value**
1	Negative regulation of transcription, DNA-dependent	54	1.02 × 10^−06^	0.000102
2	Double-stranded DNA binding	16	3.64 × 10^−06^	0.000182
3	Structure-specific DNA binding	20	1.31 × 10^−05^	0.00033
4	Sequence-specific DNA binding	41	1.32 × 10^−05^	0.00033
5	RNA polymerase II distal enhancer sequence-specific DNA binding transcription factor activity	12	5.13 × 10^−05^	0.00096
6	Transcription from RNA polymerase II promoter	81	6.07 × 10^−05^	0.00096
7	Enzyme binding	56	6.74 × 10^−05^	0.00096
8	DNA binding	107	8.38 × 10^−05^	0.00096
9	Phosphatase binding	12	8.64 × 10^−05^	0.00096
10	Chromatin binding	22	0.000184	0.001608
**D. KEGG Pathways Regulated by Downregulated miR**				
**S. No.**	**Name**	**Hits**	***p*-Value**	**Adj. *p*-Value**
1	Pathways in cancer	64	1.45 × 10^−18^	1.45 × 10^−16^
2	Prostate cancer	31	2.65 × 10^−16^	1.33 × 10^−14^
3	Chronic myeloid leukemia	26	7.80 × 10^−14^	2.60 × 10^−12^
4	Small cell lung cancer	26	9.30 × 10^−13^	2.33 × 10^−11^
5	Glioma	22	2.27 × 10^−11^	4.54 × 10^−10^
6	P53 signaling pathway	22	6.30 × 10^−11^	1.05 × 10^−09^
7	Pancreatic cancer	22	8.72 × 10^−11^	1.25 × 10^−09^
8	Cell cycle	29	3.71 × 10^−10^	4.64 × 10^−09^
9	HTLV-I infection	37	1.41 × 10^−09^	1.57 × 10^−08^
10	Melanoma	20	3.22 × 10^−09^	3.22 × 10^−08^
**E. Geneontology Enrichment for Biological Process (GO-BP) Regulated by Downregulated miR**				
**S. No.**	**Name**	**Hits**	***p*-Value**	**Adj. *p*-Value**
1	Negative regulation of transcription from RNA polymerase II promoter	74	2.97 × 10^−11^	2.97 × 10^−09^
2	Interphase of mitotic cell cycle	59	2.11 × 10^−09^	1.06 × 10^−07^
3	Interphase	59	4.22 × 10^−09^	1.41 × 10^−07^
4	Negative regulation of RNA metabolic process	105	1.76 × 10^−08^	3.22 × 10^−07^
5	Negative regulation of transcription, DNA-dependent	102	1.93 × 10^−08^	3.22 × 10^−07^
6	Negative regulation of cellular metabolic process	102	1.93 × 10^−08^	3.22 × 10^−07^
7	Regulation of cell cycle	151	4.11 × 10^−08^	5.23 × 10^−07^
8	Regulation of transcription from RNA polymerase II promoter	93	4.18 × 10^−08^	5.23 × 10^−07^
9	Negative regulation of cellular biosynthetic process	146	7.03 × 10^−08^	7.81 × 10^−07^
10	Negative regulation of metabolic process	117	1.14 × 10^−07^	1.14 × 10^−06^
**F. Gene Ontology Enrichment for Molecular Function (GO-MF) Regulated by Downregulated miR**				
**S. No.**	**Name**	**Hits**	***p*-Value**	**Adj. *p*-Value**
1	Negative regulation of transcription, DNA-dependent	102	1.72 × 10^−09^	1.72 × 10^−07^
2	Transcription from RNA polymerase II promoter	167	6.75 × 10^−09^	2.40 × 10^−07^
3	Transcription factor binding	62	9.59 × 10^−09^	2.40 × 10^−07^
4	Enzyme binding	115	1.11 × 10^−08^	2.40 × 10^−07^
5	Kinase binding	54	1.20 × 10^−08^	2.40 × 10^−07^
6	Protein kinase binding	48	1.14 × 10^−07^	1.9 × 10^−06^
7	Positive regulation of transcription, DNA-dependent	111	1.5 × 10^−06^	2.14 × 10^−05^
8	Nucleotide binding	191	2.08 × 10^−06^	0.000026
9	SMAD binding	15	4.8 × 10^−06^	5.33 × 10^−05^
10	Phosphatase binding	20	7.74 × 10^−06^	7.74 × 10^−05^

Pathway enrichment analysis for deregulated miR in ovarian cancer: Gene targets for miR were predicted using miRNet web tool (https://www.mirnet.ca/) and miRTarBase v8.0. (**A**,**D**) KEGG pathway analysis enrichment, gene ontology enrichment for (**B**,**E**) biological process (GO-BP) and (**C**,**F**) molecular function (GO-MF) were analyzed for upregulated (**A**–**C**) and downregulated (**D**–**F**) miR target genes; the 10 most significant pathways based on the network generated using degree cutoff 5 are shown.

**Table 3:** Deregulated miR in Endometrial Cancer.

Related Entity	Lit Str.	Lit MIM	Regulation	No. of Papers	PMID
miR-205	37	0.1	Up	27	[300]
miR-200C	28.7	−0.1	Up	20	[309,310]
miR-200A	16.3	0.2	Up	18	[198]
miR-200B	16.3	0.1	Up	18	[311]
miR-21	27.6	−2.4	Up	14	[312]
miR-155	19.2	−2.3	Up	11	[313]
miR-141	9	−0.4	Up	9	[314]
miR-182	14.1	−0.5	Up	9	[315]
miR-135B	7.6	−0.3	Up	5	[316]
miR-183	11.3	−0.8	Up	5	[317]
miR-106B	4.7	−1	Up	4	[318]
miR-10B	4.7	−1.3	Up	4	[319]
miR-210	5.8	−2.6	Up	4	[320]
miR-191	5	−0.9	Up	3	[321]
miR-223	4.4	−1.8	Up	3	[322]
miR-522	4.5	0.5	Up	1	[323]
miR-544A	6.1	0.2	Up	1	[323]
miR-940	4.5	−0.9	Up	1	[324]
miR-944	9.3	−0.3	Up	1	[325]
miR-152	17.3	0.2	Down	10	[326]
miR-145	15.5	−1.8	Down	9	[327]
miR-204	9.1	−0.6	Down	8	[328]
miR-143	7.6	−1.7	Down	6	[329]
miR-203A	13.4	−1.3	Down	6	[330]
miR-424	27.2	0.2	Down	6	[331]
miR-214	4.2	−2.3	Down	5	[332]
miR-23B	4.7	−1	Down	5	[333]
miR-130B	7.3	−0.3	Down	5	[334]
miR-126	7.9	−2.3	Down	4	[335]
miR-195	7.9	−1.7	Down	4	[336]
miR-137	5	−2.1	Down	3	[337]
miR-142	6.3	−0.3	Down	3	[338,339]
miR-181C	4.2	−0.8	Down	3	[340]
miR-23A	7.4	−1.9	Down	3	[341]
miR-29C	5.8	−1.6	Down	3	[342]
miR-320A	7.4	−1.2	Down	3	[343]
miR-146A	4.2	−3.3	Down	3	[344]
miR-1271	13.5	0.2	Down	2	[345]
miR-129-2	8.6	1.5	Down	2	[346]
miR-148B	8.2	−0.7	Down	2	[347]
miR-202	6.9	−1	Down	2	[348]
miR-206	5.8	−2.4	Down	2	[349]
miR-646	9.8	0.6	Down	2	[350]
miR-125A	4.5	−2.1	Down	1	[351]
miR-23C	4.2	1.4	Down	1	[352]
miR-873	10.1	−0.6	Down	1	[353]

Deregulated miR in endometrial cancer. Lit Str. and Lit MIM represent: literature strength, and literature mutual information, respectively. The miR were regrouped based on their expression. The number of publications with the miR was retrieved from PubMed, and representative manuscripts are cited.

**Table 4:** Altered Pathways by Deregulated miR in Endometrial Cancer.

A. KEGG Pathways Regulated by Upregulated miR				
S. No.	Name	Hits	*p*-Value	Adj. *p*-Value
1	Pathways in cancer	9	3.27 × 10^−08^	1.3407 × 10^−06^
2	Prostate cancer	5	0.00000347	0.000071135
3	Glioma	4	0.0000306	0.00037515
4	p53 signaling pathway	4	0.0000366	0.00037515
5	Melanoma	3	0.000952	0.006792333
6	Pancreatic cancer	3	0.000994	0.006792333
7	Small cell lung cancer	3	0.00153	0.008961429
8	Focal adhesion	4	0.00231	0.01183875
9	Bladder cancer	2	0.00322	0.01466889
10	mTOR signaling pathway	2	0.00765	0.031365
**B. Gene Ontology Enrichment for Biological Process (GO-BP) Regulated by Upregulated miR**				
**S. No.**	**Name**	**Hits**	***p*-Value**	**Adj. *p*-Value**
1	negative regulation of apoptotic process	12	1.01 × 10^−10^	4.10 × 10^−09^
2	negative regulation of programmed cell death	12	1.01 × 10^−10^	4.10 × 10^−09^
3	regulation of gene expression	12	1.23 × 10^−10^	4.10 × 10^−09^
4	apoptotic process	21	3.23 × 10^−09^	8.05 × 10^−08^
5	programmed cell death	16	4.83 × 10^−09^	8.05 × 10^−08^
6	regulation of apoptotic process	16	4.83 × 10^−09^	8.05 × 10^−08^
7	regulation of programmed cell death	16	5.83 × 10^−09^	8.33 × 10^−08^
8	cell proliferation	14	8.25 × 10^−09^	1.03 × 10^−07^
9	regulation of RNA metabolic process	14	9.61 × 10^−09^	1.07 × 10^−07^
10	regulation of nucleobase-containing compound metabolic process	15	1.20 × 10^−08^	1.20 × 10^−07^
**C. Gene Ontology Enrichment for Molecular Function (GO-MF) Regulated by Upregulated miR**				
**S. No.**	**Name**	**Hits**	***p*-Value**	**Adj. *p*-Value**
1	negative regulation of transcription, DNA-dependent	10	0.00000168	0.00016128
2	transcription from RNA polymerase II promoter	12	0.0000173	0.0008304
3	protein kinase binding	5	0.000325	0.0101376
4	enzyme binding	8	0.000476	0.0101376
5	kinase binding	5	0.000528	0.0101376
6	RNA polymerase II distal enhancer sequence-specific DNA binding transcription factor activity	3	0.000768	0.012288
7	sequence-specific DNA binding	6	0.001	0.01365333
8	ubiquitin-protein ligase activity	4	0.00124	0.01365333
9	transcription factor binding	5	0.00128	0.01365333
10	small conjugating protein ligase activity	4	0.00162	0.01413818
**D. KEGG Pathways Regulated by Downregulated miR**				
**S. No.**	**Name**	**Hits**	***p*-Value**	**Adj. *p*-Value**
1	Chronic myeloid leukemia	11	9.87 × 10^−12^	9.57 × 10^−10^
2	Pathways in cancer	17	1.30 × 10^−10^	6.31 × 10^−09^
3	Colorectal cancer	8	4.76 × 10^−09^	1.54 × 10^−07^
4	Glioma	8	4.83 × 10^−08^	1.17 × 10^−06^
5	Melanoma	8	6.94 × 10^−08^	0.000001261
6	Pancreatic cancer	8	7.80 × 10^−08^	0.000001261
7	Prostate cancer	8	4.88 × 10^−07^	6.22 × 10^−06^
8	Focal adhesion	11	5.13 × 10^−07^	6.22 × 10^−06^
9	Thyroid cancer	5	0.000003	3.23 × 10^−05^
10	ErbB signaling pathway	7	0.00000682	0.000066154
**E. Gene Ontology Enrichment for Biological Process (GO-BP) Regulated by Downregulated miR**				
**S. No.**	**Name**	**Hits**	***p*-Value**	**Adj. *p*-Value**
1	regulation of cell proliferation	27	5.61 × 10^−07^	0.000023725
2	positive regulation of metabolic process	39	8.03 × 10^−07^	0.000023725
3	regulation of cellular protein metabolic process	28	9.08 × 10^−07^	0.000023725
4	gland development	12	9.49 × 10^−07^	0.000023725
5	tissue morphogenesis	16	0.0000012	0.000024
6	morphogenesis of an epithelium	14	0.00000152	2.444 × 10^−05^
7	negative regulation of cell proliferation	16	0.00000185	2.444 × 10^−05^
8	negative regulation of metabolic process	30	0.00000206	2.444 × 10^−05^
9	regulation of kinase activity	18	0.0000022	2.444 × 10^−05^
10	enzyme linked receptor protein signaling pathway	23	0.00000271	2.692 × 10^−05^
**F. Gene Ontology Enrichment for Molecular Function (GO-MF) Regulated by Downregulated miR**				
**S. No.**	**Name**	**Hits**	***p*-Value**	**Adj. *p*-Value**
1	protein complex binding	10	0.0000896	0.00896
2	transcription from RNA polymerase II promoter	25	0.000725	0.02716667
3	negative regulation of transcription, DNA-dependent	16	0.000815	0.02716667
4	SMAD binding	4	0.00111	0.02775
5	kinase binding	9	0.00194	0.0388
6	protein kinase binding	8	0.00372	0.062
7	SH3/SH2 adaptor activity	3	0.00508	0.07257143
8	transcription factor binding	9	0.00705	0.085
9	phosphatase binding	4	0.00765	0.085
10	cytokine receptor binding	6	0.00892	0.0892

Pathway enrichment analysis for deregulated miR in endometrial cancer: Gene targets for miR were predicted using the miRNet web tool (https://www.mirnet.ca/) and miRTarBase v8.0. (**A**,**D**) KEGG pathway analysis enrichment, gene ontology enrichment for (**B**,**E**) biological process (GO-BP) and (**C**,**F**) molecular function (GO-MF) were analyzed for upregulated (**A**–**C**) and downregulated (**D**–**F**) miR target genes, the 10 most significant pathways based on the network generated using degree cutoff 5 are shown.

**Table 5:** Deregulated miR in Cervical Cancer.

Related Entity	Lit Str.	Lit MIM	Regulation	No. of Papers	PMID
miR-21	136.6	−1.6	Up	61	[383]
miR-205	31.8	−0.9	Up	20	[384]
miR-155	36.1	−2.5	Up	19	[383]
miR-20A	27.8	−0.6	Up	17	[356]
miR-126	30	−1.6	Up	12	[385]
miR-200B	16.5	−1.1	Up	9	[386]
miR-150	10.5	−1.9	Up	8	[363]
miR-200A	9.4	−1.4	Up	8	[387]
miR-944	13.7	0.6	Up	7	[388]
miR-130A	20.6	−1	Up	6	[389]
miR-106B	18.4	−0.7	Up	6	[390]
miR-27B	12.9	−1.3	Up	6	[391]
miR-15B	7.8	−0.9	Up	6	[392]
miR-133B	7.6	−1.6	Up	6	[393]
miR-18A	13.2	−1.6	Up	5	[394]
miR-106A	11.8	−0.9	Up	5	[357]
miR-499A	9.8	−0.7	Up	4	[395]
miR-152	9.5	−1.6	Up	4	[396]
miR-135B	8.7	−1.3	Up	4	[397]
miR-210	6.8	−2.5	Up	4	[398]
miR-940	5.5	−0.5	Up	4	[399]
miR-149	4.1	−1	Up	4	[400]
miR-130B	7.1	−1.4	Up	3	[401]
miR-1290	3.9	−0.6	Up	3	[402]
miR-373	13.8	−1.5	Up	2	[403]
miR-492	7.4	−0.3	Up	2	[236]
miR-205HG	5.8	0.9	Up	2	[404]
miR-127	4.2	−1.1	Up	2	[405]
miR-92B	3.7	−1.6	Up	2	[406]
miR-613	3.4	−1.3	Up	2	[407]
miR-301B	7.4	0	Up	1	[408]
miR-501	6.9	0	Up	1	[409]
miR-3142	5.3	1.4	Up	1	[410]
miR-519D	5.3	−1.2	Up	1	[411]
miR-629	4.5	−0.5	Up	1	[412]
miR-421	4.2	−1.2	Up	1	[413]
miR-145	64.5	−1.2	Down	29	[414]
miR-143	44	−0.6	Down	28	[150]
miR-146A	27.4	−1.7	Down	22	[415]
miR-203A	25	−0.9	Down	22	[416]
miR-214	44.8	−0.9	Down	20	[417]
miR-195	40.1	−0.9	Down	14	[418]
miR-29A	23.6	−1.7	Down	13	[419]
miR-424	18.4	−0.4	Down	12	[420]
miR-7	11	−2.1	Down	12	[421]
miR-206	19.2	−1.7	Down	10	[422]
miR-22	25.3	−1.2	Down	9	[423]
miR-23B	21.4	−1	Down	9	[424]
miR-182	18.7	−1.5	Down	9	[425]
miR-497	16.3	−0.9	Down	8	[426]
miR-183	10	−1.7	Down	7	[427]
miR-125A	10	−1.4	Down	6	[428]
miR-204	14.8	−1.9	Down	5	[429]
miR-144	13.7	−1.4	Down	5	[359]
miR-506	12.9	−0.6	Down	5	[430]
miR-187	10.8	−0.2	Down	5	[431]
miR-132	8.1	−2	Down	5	[432]
miR-223	6	−2.5	Down	5	[433]
miR-215	6.3	−1	Down	4	[434]
miR-216B	4.5	−1.8	Down	4	[435]
miR-10B	14.3	−2	Down	3	[436]
miR-26B	13	−2.1	Down	3	[437]
miR-217	12.7	−1.6	Down	3	[438]
miR-432	11.9	0.5	Down	3	[439]
miR-641	11.1	0.5	Down	3	[440]
miR-744	10	−0.1	Down	3	[441]
miR-15A	8.6	−1.5	Down	3	[442]
miR-200C	6.3	−2.6	Down	3	[443]
miR-186	5.5	−1.2	Down	3	[444]
miR-30A	5.5	−2	Down	3	[445]
miR-142	5	−1.4	Down	3	[446]
miR-196B	4.2	−1.3	Down	3	[447]
miR-4429	4.2	1.5	Down	3	[448]
miR-1284	8.2	0.4	Down	2	[449]
miR-612	6.9	−0.9	Down	2	[450]
miR-873	6.6	−0.6	Down	2	[451]
miR-504	6.1	−1.1	Down	2	[452]
miR-802	6.1	−1.4	Down	2	[453]
miR-99B	6.1	−1.3	Down	2	[454]
miR-320A	6	−1.2	Down	2	[289]
miR-374B	5.8	−0.3	Down	2	[455]
miR-1297	5	−0.4	Down	2	[456]
miR-2861	5	0.2	Down	2	[457]
miR-760	5	−0.7	Down	2	[458]
miR-136	4.2	−1	Down	2	[459]
miR-498	4.2	−0.8	Down	2	[460]
miR-758	4.2	0.3	Down	2	[461]
miR-372	3.4	−1	Down	2	[462]
miR-137	3.1	−2.4	Down	2	[463]
miR-148A	8.7	−2.1	Down	1	[464]
miR-877	7.7	−0.2	Down	1	[465]
miR-889	6.1	−0.2	Down	1	[466]
miR-1202	5.3	−0.3	Down	1	[467]
miR-1294	5.3	−0.8	Down	1	[468]
miR-3148	5.3	1.1	Down	1	[469]
miR-636	5.3	0.1	Down	1	[470]
miR-376C	4.5	−0.9	Down	1	[471]
miR-584	4.5	−0.6	Down	1	[472]
miR-LET7B	3.9	−1.8	Down	1	[473]
miR-134	3.7	−2.6	Down	1	[474]
miR-147A	3.7	0.4	Down	1	[475]
miR-197	3.7	−1.6	Down	1	[476]
miR-6893	3.7	2.4	Down	1	[477]
miR-8075	3.7	2.4	Down	1	[478]
miR-140	3.1	−1.5	Down	1	[479]
miR-630	7.4	−0.9	Down	1	[480]

Deregulated miR in cervical cancer. Lit Str. and Lit MIM represent: literature strength, and literature mutual information, respectively. The miR were regrouped based on their expression. The number of publications with the miR was retrieved from PubMed, and representative manuscripts are cited.

**Table 6:** Altered Pathways by Deregulated miR in Cervical Cancer.

A. KEGG Pathways Regulated by Upregulated miR				
S. No.	Name	Hits	*p*-Value	Adj. *p*-Value
1	p53 signaling pathway	16	1.29 × 10^−09^	1.29 × 10^−07^
2	Bladder cancer	10	3.39 × 10^−08^	1.2867 × 10^−06^
3	Pathways in cancer	32	3.86 × 10^−08^	1.2867 × 10^−06^
4	Chronic myeloid leukemia	14	2.33 × 10^−07^	5.825 × 10^−06^
5	Prostate cancer	15	3.67 × 10^−07^	0.0000064
6	Glioma	13	3.84 × 10^−07^	0.0000064
7	Pancreatic cancer	12	0.0000052	7.4286 × 10^−05^
8	Cell cycle	16	0.00000806	0.00010075
9	Small cell lung cancer	12	0.0000251	0.000266
10	Melanoma	11	0.0000266	0.000266
**B. Gene Ontology Enrichment for Biological Process (GO-BP) Regulated by Upregulated miR**				
**S. No.**	**Name**	**Hits**	***p*-Value**	**Adj. *p*-Value**
1	negative regulation of transcription from RNA polymerase II promoter	49	1.12 × 10^−08^	0.00000112
2	DNA-dependent transcription, initiation	26	0.00000331	0.000096
3	regulation of transcription from RNA polymerase II promoter	93	0.00000641	0.000096
4	transcription initiation from RNA polymerase II promoter	23	0.00000653	0.000096
5	G1 phase of mitotic cell cycle	10	0.00000658	0.000096
6	negative regulation of transcription, DNA-dependent	64	0.00000693	0.000096
7	negative regulation of cellular biosynthetic process	64	0.00000693	0.000096
8	regulation of gene expression	75	0.00000768	0.000096
9	G1 phase	212	0.00000867	9.6333 × 10^−05^
10	regulation of translation	10	0.00000977	0.0000977
**C. Gene Ontology Enrichment for Molecular Function (GO-MF) Regulated by Upregulated miR**				
**S. No.**	**Name**	**Hits**	***p*-Value**	**Adj. *p*-Value**
1	negative regulation of transcription, DNA-dependent	64	0.00000398	0.000398
2	DNA binding	140	0.00000949	0.0004745
3	transcription from RNA polymerase II promoter	102	0.0000457	0.0012075
4	transcription factor binding	37	0.0000483	0.0012075
5	phosphatase binding	14	0.000079	0.00158
6	sequence-specific DNA binding	46	0.000203	0.00338333
7	enzyme binding	67	0.000247	0.00352857
8	double-stranded DNA binding	15	0.000344	0.0043
9	structure-specific DNA binding	20	0.000544	0.00604444
10	RNA polymerase II distal enhancer sequence-specific DNA binding transcription factor activity	12	0.000643	0.00643
**D. KEGG Pathways Regulated by Downregulated miR**				
**S. No.**	**Name**	**Hits**	***p*-Value**	**Adj. *p*-Value**
1	Pathways in cancer	61	7.79 × 10^−17^	7.79 × 10^−15^
2	Prostate cancer	30	1.77 × 10^−15^	8.85 × 10^−14^
3	Cell cycle	34	5.07 × 10^−14^	1.69 × 10^−12^
4	Colorectal cancer	20	2.79 × 10^−12^	6.98 × 10^−11^
5	Chronic myeloid leukemia	23	3.41 × 10^−11^	6.82 × 10^−10^
6	Pancreatic cancer	22	7.14 × 10^−11^	1.19 × 10^−09^
7	Glioma	21	1.48 × 10^−10^	2.11 × 10^−09^
8	Neurotrophin signaling pathway	29	2.36 × 10^−10^	2.95 × 10^−09^
9	Focal adhesion	38	3.17 × 10^−10^	3.52 × 10^−09^
10	Endometrial cancer	17	3.62 × 10^−10^	3.62 × 10^−09^
**E. Gene Ontology Enrichment for Biological Process (GO-BP) Regulated by Downregulated miR**				
**S. No.**	**Name**	**Hits**	***p*-Value**	**Adj. *p*-Value**
1	regulation of cellular protein metabolic process	141	1.46 × 10^−10^	6.50 × 10^−09^
2	negative regulation of programmed cell death	78	2.45 × 10^−10^	6.50 × 10^−09^
3	negative regulation of apoptotic process	77	2.60 × 10^−10^	6.50 × 10^−09^
4	regulation of cell cycle	77	2.60 × 10^−10^	6.50 × 10^−09^
5	negative regulation of RNA metabolic process	91	1.04 × 10^−09^	1.51 × 10^−08^
6	negative regulation of cellular biosynthetic process	101	1.06 × 10^−09^	1.51 × 10^−08^
7	negative regulation of transcription, DNA-dependent	115	1.06 × 10^−09^	1.51 × 10^−08^
8	negative regulation of biosynthetic process	98	1.36 × 10^−09^	1.51 × 10^−08^
9	regulation of translation	98	1.36 × 10^−09^	1.51 × 10^−08^
10	gland development	115	2.75 × 10^−09^	2.75 × 10^−08^
**F. Gene Ontology Enrichment for Molecular Function (GO-MF) Regulated by Downregulated miR**				
**S. No.**	**Name**	**Hits**	***p*-Value**	**Adj. *p*-Value**
1	negative regulation of transcription, DNA-dependent	98	9.93 × 10^−11^	9.93 × 10^−09^
2	enzyme binding	110	7.08 × 10^−10^	3.54 × 10^−08^
3	SMAD binding	18	5.26 × 10^−09^	1.59 × 10^−07^
4	kinase binding	51	6.34 × 10^−09^	1.59 × 10^−07^
5	sequence-specific DNA binding	73	2.57 × 10^−08^	5.14 × 10^−07^
6	protein kinase binding	46	3.29 × 10^−08^	5.48 × 10^−07^
7	nucleotide binding	182	5.93 × 10^−08^	8.47 × 10^−07^
8	positive regulation of transcription, DNA-dependent	106	1.40 × 10^−07^	0.00000175
9	transcription from RNA polymerase II promoter	146	3.75 × 10^−07^	4.1667 × 10^−06^
10	transcription factor binding	53	6.16 × 10^−07^	0.00000616

Pathway enrichment analysis for deregulated miR in cervical cancer: Gene targets for miR were predicted using the miRNet web tool (https://www.mirnet.ca/) and miRTarBase v8.0. (**A**,**D**) KEGG pathway analysis enrichment, gene ontology enrichment for (**B**,**E**) biological process (GO-BP), and (**C**,**F**) molecular function (GO-MF) were analyzed for upregulated (**A**–**C**) and downregulated (**D**–**F**) miR target genes, the 10 most significant pathways based on the network generated using degree cutoff 5 are shown.

## Data Availability

Available on request.

## References

[1] Brooks R.A., Fleming G.F., Lastra R.R., Lee N.K., Moroney J.W., Son C.H., Tatebe K., Veneris J.L. (2019). Current recommendations and recent progress in endometrial cancer. CA Cancer J. Clin..

[2] Stewart C., Ralyea C., Lockwood S. (2019). Ovarian Cancer: An Integrated Review. Semin. Oncol. Nurs..

[3] Matulonis U.A., Sood A.K., Fallowfield L., Howitt B.E., Sehouli J., Karlan B.Y. (2016). Ovarian cancer. Nat. Rev. Dis. Primers.

[4] Tsikouras P., Zervoudis S., Manav B., Tomara E., Iatrakis G., Romanidis C., Bothou A., Galazios G. (2016). Cervical cancer: Screening, diagnosis and staging. J. BUON.

[5] Arbyn M., Weiderpass E., Bruni L., de Sanjose S., Saraiya M., Ferlay J., Bray F. (2020). Estimates of incidence and mortality of cervical cancer in 2018: A worldwide analysis. Lancet Glob. Health.

[6] Anttila T., Saikku P., Koskela P., Bloigu A., Dillner J., Ikaheimo I., Jellum E., Lehtinen M., Lenner P., Hakulinen T. (2001). Serotypes of Chlamydia trachomatis and risk for development of cervical squamous cell carcinoma. JAMA.

[7] Hu K., Wang W., Liu X., Meng Q., Zhang F. (2018). Comparison of treatment outcomes between squamous cell carcinoma and adenocarcinoma of cervix after definitive radiotherapy or concurrent chemoradiotherapy. Radiat. Oncol..

[8] Janicek M.F., Averette H.E. (2001). Cervical cancer: Prevention, diagnosis, and therapeutics. CA Cancer J. Clin..

[9] Han X., Wen H., Ju X., Chen X., Ke G., Zhou Y., Li J., Xia L., Tang J., Liang S. (2017). Predictive factors of para-aortic lymph nodes metastasis in cervical cancer patients: A retrospective analysis based on 723 para-aortic lymphadenectomy cases. Oncotarget.

[10] Li H., Wu X., Cheng X. (2016). Advances in diagnosis and treatment of metastatic cervical cancer. J. Gynecol. Oncol..

[11] Durst M., Gissmann L., Ikenberg H., zur Hausen H. (1983). A papillomavirus DNA from a cervical carcinoma and its prevalence in cancer biopsy samples from different geographic regions. Proc. Natl. Acad. Sci. USA.

[12] Hietanen S., Lain S., Krausz E., Blattner C., Lane D.P. (2000). Activation of p53 in cervical carcinoma cells by small molecules. Proc. Natl. Acad. Sci. USA.

[13] Varughese J., Richman S. (2010). Cancer care inequity for women in resource-poor countries. Rev. Obstet. Gynecol..

[14] Sorosky J.I. (2012). Endometrial cancer. Obstet. Gynecol..

[15] Talhouk A., McAlpine J.N. (2016). New classification of endometrial cancers: The development and potential applications of genomic-based classification in research and clinical care. Gynecol. Oncol. Res. Pract..

[16] Kajo K., Vallova M., Biro C., Bognar G., Machalekova K., Zavodna K., Galbavy S., Zubor P. (2015). Molecular pathology of endometrial carcinoma—A review. Cesk. Patol..

[17] Levine D.A. (2013). Integrated genomic characterization of endometrial carcinoma. Nature.

[18] McAlpine J., Leon-Castillo A., Bosse T. (2018). The rise of a novel classification system for endometrial carcinoma; integration of molecular subclasses. J. Pathol..

[19] Devouassoux-Shisheboran M., Genestie C. (2015). Pathobiology of ovarian carcinomas. Chin. J. Cancer.

[20] Labidi-Galy S.I., Papp E., Hallberg D., Niknafs N., Adleff V., Noe M., Bhattacharya R., Novak M., Jones S., Phallen J. (2017). High grade serous ovarian carcinomas originate in the fallopian tube. Nat. Commun..

[21] Kim O., Park E.Y., Kwon S.Y., Shin S., Emerson R.E., Shin Y.H., DeMayo F.J., Lydon J.P., Coffey D.M., Hawkins S.M. (2020). Targeting progesterone signaling prevents metastatic ovarian cancer. Proc. Natl. Acad. Sci. USA.

[22] Dochez V., Caillon H., Vaucel E., Dimet J., Winer N., Ducarme G. (2019). Biomarkers and algorithms for diagnosis of ovarian cancer: CA125, HE4, RMI and ROMA, a review. J. Ovarian Res..

[23] Hellstrom I., Raycraft J., Hayden-Ledbetter M., Ledbetter J.A., Schummer M., McIntosh M., Drescher C., Urban N., Hellstrom K.E. (2003). The HE4 (WFDC2) protein is a biomarker for ovarian carcinoma. Cancer Res..

[24] Bodnar L., Stanczak A., Cierniak S., Smoter M., Cichowicz M., Kozlowski W., Szczylik C., Wieczorek M., Lamparska-Przybysz M. (2014). Wnt/beta-catenin pathway as a potential prognostic and predictive marker in patients with advanced ovarian cancer. J. Ovarian Res..

[25] Brachova P., Mueting S.R., Carlson M.J., Goodheart M.J., Button A.M., Mott S.L., Dai D., Thiel K.W., Devor E.J., Leslie K.K. (2015). TP53 oncomorphic mutations predict resistance to platinum and taxanebased standard chemotherapy in patients diagnosed with advanced serous ovarian carcinoma. Int. J. Oncol..

[26] Steinberga I., Jansson K., Sorbe B. (2019). Quality Indicators and Survival Outcome in Stage IIIB-IVB Epithelial Ovarian Cancer Treated at a Single Institution. In Vivo.

[27] Djebali S., Davis C.A., Merkel A., Dobin A., Lassmann T., Mortazavi A., Tanzer A., Lagarde J., Lin W., Schlesinger F. (2012). Landscape of transcription in human cells. Nature.

[28] Rinn J.L., Chang H.Y. (2012). Genome regulation by long noncoding RNAs. Annu. Rev. Biochem..

[29] Nagano T., Fraser P. (2011). No-nonsense functions for long noncoding RNAs. Cell.

[30] He L., Hannon G.J. (2004). MicroRNAs: Small RNAs with a big role in gene regulation. Nat. Rev. Genet..

[31] Bartel D.P. (2004). MicroRNAs: Genomics, biogenesis, mechanism, and function. Cell.

[32] Ghildiyal M., Zamore P.D. (2009). Small silencing RNAs: An expanding universe. Nat. Rev. Genet..

[33] Ozata D.M., Gainetdinov I., Zoch A., O’Carroll D., Zamore P.D. (2019). PIWI-interacting RNAs: Small RNAs with big functions. Nat. Rev. Genet..

[34] Aravin A.A., Naumova N.M., Tulin A.V., Vagin V.V., Rozovsky Y.M., Gvozdev V.A. (2001). Double-stranded RNA-mediated silencing of genomic tandem repeats and transposable elements in the D. melanogaster germline. Curr. Biol..

[35] Weick E.M., Miska E.A. (2014). piRNAs: From biogenesis to function. Development.

[36] Lim S.L., Qu Z.P., Kortschak R.D., Lawrence D.M., Geoghegan J., Hempfling A.L., Bergmann M., Goodnow C.C., Ormandy C.J., Wong L. (2015). HENMT1 and piRNA Stability Are Required for Adult Male Germ Cell Transposon Repression and to Define the Spermatogenic Program in the Mouse. PLoS Genet..

[37] Billi A.C., Alessi A.F., Khivansara V., Han T., Freeberg M., Mitani S., Kim J.K. (2012). The Caenorhabditis elegans HEN1 ortholog, HENN-1, methylates and stabilizes select subclasses of germline small RNAs. PLoS Genet..

[38] Montgomery T.A., Rim Y.S., Zhang C., Dowen R.H., Phillips C.M., Fischer S.E., Ruvkun G. (2012). PIWI associated siRNAs and piRNAs specifically require the Caenorhabditis elegans HEN1 ortholog henn-1. PLoS Genet..

[39] Batista P.J., Ruby J.G., Claycomb J.M., Chiang R., Fahlgren N., Kasschau K.D., Chaves D.A., Gu W., Vasale J.J., Duan S. (2008). PRG-1 and 21U-RNAs interact to form the piRNA complex required for fertility in C. elegans. Mol. Cell.

[40] Das P.P., Bagijn M.P., Goldstein L.D., Woolford J.R., Lehrbach N.J., Sapetschnig A., Buhecha H.R., Gilchrist M.J., Howe K.L., Stark R. (2008). Piwi and piRNAs act upstream of an endogenous siRNA pathway to suppress Tc3 transposon mobility in the Caenorhabditis elegans germline. Mol. Cell.

[41] Saito K., Nishida K.M., Mori T., Kawamura Y., Miyoshi K., Nagami T., Siomi H., Siomi M.C. (2006). Specific association of Piwi with rasiRNAs derived from retrotransposon and heterochromatic regions in the Drosophila genome. Genes Dev..

[42] Grivna S.T., Beyret E., Wang Z., Lin H. (2006). A novel class of small RNAs in mouse spermatogenic cells. Genes Dev..

[43] Aravin A., Gaidatzis D., Pfeffer S., Lagos-Quintana M., Landgraf P., Iovino N., Morris P., Brownstein M.J., Kuramochi-Miyagawa S., Nakano T. (2006). A novel class of small RNAs bind to MILI protein in mouse testes. Nature.

[44] Juliano C., Wang J., Lin H. (2011). Uniting germline and stem cells: The function of Piwi proteins and the piRNA pathway in diverse organisms. Annu. Rev. Genet..

[45] Houwing S., Berezikov E., Ketting R.F. (2008). Zili is required for germ cell differentiation and meiosis in zebrafish. EMBO J..

[46] Carmell M.A., Girard A., van de Kant H.J., Bourc’his D., Bestor T.H., de Rooij D.G., Hannon G.J. (2007). MIWI2 is essential for spermatogenesis and repression of transposons in the mouse male germline. Dev. Cell.

[47] Kuramochi-Miyagawa S., Kimura T., Ijiri T.W., Isobe T., Asada N., Fujita Y., Ikawa M., Iwai N., Okabe M., Deng W. (2004). Mili, a mammalian member of piwi family gene, is essential for spermatogenesis. Development.

[48] Deng W., Lin H. (2002). Miwi, a murine homolog of piwi, encodes a cytoplasmic protein essential for spermatogenesis. Dev. Cell.

[49] Vagin V.V., Sigova A., Li C., Seitz H., Gvozdev V., Zamore P.D. (2006). A distinct small RNA pathway silences selfish genetic elements in the germline. Science.

[50] Girard A., Sachidanandam R., Hannon G.J., Carmell M.A. (2006). A germline-specific class of small RNAs binds mammalian Piwi proteins. Nature.

[51] Cox D.N., Chao A., Lin H. (2000). piwi encodes a nucleoplasmic factor whose activity modulates the number and division rate of germline stem cells. Development.

[52] Schupbach T., Wieschaus E. (1991). Female sterile mutations on the second chromosome of Drosophila melanogaster. II. Mutations blocking oogenesis or altering egg morphology. Genetics.

[53] Aravin A.A., Sachidanandam R., Girard A., Fejes-Toth K., Hannon G.J. (2007). Developmentally regulated piRNA clusters implicate MILI in transposon control. Science.

[54] Lau N.C., Seto A.G., Kim J., Kuramochi-Miyagawa S., Nakano T., Bartel D.P., Kingston R.E. (2006). Characterization of the piRNA complex from rat testes. Science.

[55] Zhao S., Gou L.T., Zhang M., Zu L.D., Hua M.M., Hua Y., Shi H.J., Li Y., Li J., Li D. (2013). piRNA-triggered MIWI ubiquitination and removal by APC/C in late spermatogenesis. Dev. Cell.

[56] Lin H., Spradling A.C. (1997). A novel group of pumilio mutations affects the asymmetric division of germline stem cells in the Drosophila ovary. Development.

[57] Iwasaki Y.W., Murano K., Ishizu H., Shibuya A., Iyoda Y., Siomi M.C., Siomi H., Saito K. (2016). Piwi Modulates Chromatin Accessibility by Regulating Multiple Factors Including Histone H1 to Repress Transposons. Mol. Cell.

[58] Chen C., Liu J., Xu G. (2013). Overexpression of PIWI proteins in human stage III epithelial ovarian cancer with lymph node metastasis. Cancer Biomark..

[59] Wang Q.E., Han C., Milum K., Wani A.A. (2011). Stem cell protein Piwil2 modulates chromatin modifications upon cisplatin treatment. Mutat. Res..

[60] Liu W.K., Jiang X.Y., Zhang Z.X. (2010). Expression of PSCA, PIWIL1 and TBX2 and its correlation with HPV16 infection in formalin-fixed, paraffin-embedded cervical squamous cell carcinoma specimens. Arch. Virol..

[61] Simpson A.J., Caballero O.L., Jungbluth A., Chen Y.T., Old L.J. (2005). Cancer/testis antigens, gametogenesis and cancer. Nat. Rev. Cancer.

[62] Wang C., Gu Y., Zhang K., Xie K., Zhu M., Dai N., Jiang Y., Guo X., Liu M., Dai J. (2016). Systematic identification of genes with a cancer-testis expression pattern in 19 cancer types. Nat. Commun..

[63] Reeves M.E., Firek M., Jliedi A., Amaar Y.G. (2017). Identification and characterization of RASSF1C piRNA target genes in lung cancer cells. Oncotarget.

[64] Ross R.J., Weiner M.M., Lin H. (2014). PIWI proteins and PIWI-interacting RNAs in the soma. Nature.

[65] Cheng J., Deng H., Xiao B., Zhou H., Zhou F., Shen Z., Guo J. (2012). piR-823, a novel non-coding small RNA, demonstrates in vitro and in vivo tumor suppressive activity in human gastric cancer cells. Cancer Lett..

[66] Lee J.H., Jung C., Javadian-Elyaderani P., Schweyer S., Schutte D., Shoukier M., Karimi-Busheri F., Weinfeld M., Rasouli-Nia A., Hengstler J.G. (2010). Pathways of proliferation and antiapoptosis driven in breast cancer stem cells by stem cell protein piwil2. Cancer Res..

[67] Fu A., Jacobs D.I., Hoffman A.E., Zheng T., Zhu Y. (2015). PIWI-interacting RNA 021285 is involved in breast tumorigenesis possibly by remodeling the cancer epigenome. Carcinogenesis.

[68] Fu A., Jacobs D.I., Zhu Y. (2014). Epigenome-wide analysis of piRNAs in gene-specific DNA methylation. RNA Biol..

[69] Siddiqi S., Matushansky I. (2012). Piwis and piwi-interacting RNAs in the epigenetics of cancer. J. Cell Biochem..

[70] Wilson A.S., Power B.E., Molloy P.L. (2007). DNA hypomethylation and human diseases. Biochim. Biophys. Acta.

[71] Baylin S.B. (2005). DNA methylation and gene silencing in cancer. Nat. Clin. Pract. Oncol..

[72] Baylin S.B., Jones P.A. (2011). A decade of exploring the cancer epigenome—Biological and translational implications. Nat. Rev. Cancer.

[73] Singh G., Roy J., Rout P., Mallick B. (2018). Genome-wide profiling of the PIWI-interacting RNA-mRNA regulatory networks in epithelial ovarian cancers. PLoS ONE.

[74] Ravo M., Cordella A., Rinaldi A., Bruno G., Alexandrova E., Saggese P., Nassa G., Giurato G., Tarallo R., Marchese G. (2015). Small non-coding RNA deregulation in endometrial carcinogenesis. Oncotarget.

[75] Cheng J., Guo J.M., Xiao B.X., Miao Y., Jiang Z., Zhou H., Li Q.N. (2011). piRNA, the new non-coding RNA, is aberrantly expressed in human cancer cells. Clin. Chim. Acta.

[76] Liu W., Gao Q., Chen K., Xue X., Li M., Chen Q., Zhu G., Gao Y. (2014). Hiwi facilitates chemoresistance as a cancer stem cell marker in cervical cancer. Oncol. Rep..

[77] He G., Chen L., Ye Y., Xiao Y., Hua K., Jarjoura D., Nakano T., Barsky S.H., Shen R., Gao J.X. (2010). Piwil2 expressed in various stages of cervical neoplasia is a potential complementary marker for p16. Am. J. Transl. Res..

[78] Liu W.K., Jiang X.Y., Zhang Z.X. (2010). Expression of PSCA, PIWIL1, and TBX2 in endometrial adenocarcinoma. Onkologie.

[79] Gordeeva O. (2018). Cancer-testis antigens: Unique cancer stem cell biomarkers and targets for cancer therapy. Semin. Cancer Biol..

[80] Li S., Xu Z., Sheng J. (2018). tRNA-Derived Small RNA: A Novel Regulatory Small Non-Coding RNA. Genes.

[81] Balatti V., Pekarsky Y., Croce C.M. (2017). Role of the tRNA-Derived Small RNAs in Cancer: New Potential Biomarkers and Target for Therapy. Adv. Cancer Res..

[82] Karousi P., Katsaraki K., Papageorgiou S.G., Pappa V., Scorilas A., Kontos C.K. (2019). Identification of a novel tRNA-derived RNA fragment exhibiting high prognostic potential in chronic lymphocytic leukemia. Hematol. Oncol..

[83] Cole C., Sobala A., Lu C., Thatcher S.R., Bowman A., Brown J.W., Green P.J., Barton G.J., Hutvagner G. (2009). Filtering of deep sequencing data reveals the existence of abundant Dicer-dependent small RNAs derived from tRNAs. RNA.

[84] Maute R.L., Schneider C., Sumazin P., Holmes A., Califano A., Basso K., Dalla-Favera R. (2013). tRNA-derived microRNA modulates proliferation and the DNA damage response and is down-regulated in B cell lymphoma. Proc. Natl. Acad. Sci. USA.

[85] Goodarzi H., Liu X., Nguyen H.C., Zhang S., Fish L., Tavazoie S.F. (2015). Endogenous tRNA-Derived Fragments Suppress Breast Cancer Progression via YBX1 Displacement. Cell.

[86] Telonis A.G., Loher P., Honda S., Jing Y., Palazzo J., Kirino Y., Rigoutsos I. (2015). Dissecting tRNA-derived fragment complexities using personalized transcriptomes reveals novel fragment classes and unexpected dependencies. Oncotarget.

[87] Haussecker D., Huang Y., Lau A., Parameswaran P., Fire A.Z., Kay M.A. (2010). Human tRNA-derived small RNAs in the global regulation of RNA silencing. RNA.

[88] Thompson D.M., Lu C., Green P.J., Parker R. (2008). tRNA cleavage is a conserved response to oxidative stress in eukaryotes. RNA.

[89] Fu H., Feng J., Liu Q., Sun F., Tie Y., Zhu J., Xing R., Sun Z., Zheng X. (2009). Stress induces tRNA cleavage by angiogenin in mammalian cells. FEBS Lett..

[90] Yamasaki S., Ivanov P., Hu G.F., Anderson P. (2009). Angiogenin cleaves tRNA and promotes stress-induced translational repression. J. Cell Biol..

[91] Honda S., Loher P., Shigematsu M., Palazzo J.P., Suzuki R., Imoto I., Rigoutsos I., Kirino Y. (2015). Sex hormone-dependent tRNA halves enhance cell proliferation in breast and prostate cancers. Proc. Natl. Acad. Sci. USA.

[92] Selitsky S.R., Baran-Gale J., Honda M., Yamane D., Masaki T., Fannin E.E., Guerra B., Shirasaki T., Shimakami T., Kaneko S. (2015). Small tRNA-derived RNAs are increased and more abundant than microRNAs in chronic hepatitis B and C. Sci. Rep..

[93] Ivanov P., Emara M.M., Villen J., Gygi S.P., Anderson P. (2011). Angiogenin-induced tRNA fragments inhibit translation initiation. Mol. Cell.

[94] Tuorto F., Liebers R., Musch T., Schaefer M., Hofmann S., Kellner S., Frye M., Helm M., Stoecklin G., Lyko F. (2012). RNA cytosine methylation by Dnmt2 and NSun2 promotes tRNA stability and protein synthesis. Nat. Struct. Mol. Biol..

[95] Schaefer M., Pollex T., Hanna K., Tuorto F., Meusburger M., Helm M., Lyko F. (2010). RNA methylation by Dnmt2 protects transfer RNAs against stress-induced cleavage. Genes Dev..

[96] Rounge T.B., Furu K., Skotheim R.I., Haugen T.B., Grotmol T., Enerly E. (2015). Profiling of the small RNA populations in human testicular germ cell tumors shows global loss of piRNAs. Mol. Cancer.

[97] Sobala A., Hutvagner G. (2013). Small RNAs derived from the 5′ end of tRNA can inhibit protein translation in human cells. RNA Biol..

[98] Telonis A.G., Loher P., Magee R., Pliatsika V., Londin E., Kirino Y., Rigoutsos I. (2019). tRNA Fragments Show Intertwining with mRNAs of Specific Repeat Content and Have Links to Disparities. Cancer Res..

[99] Gebetsberger J., Wyss L., Mleczko A.M., Reuther J., Polacek N. (2017). A tRNA-derived fragment competes with mRNA for ribosome binding and regulates translation during stress. RNA Biol..

[100] Schopman N.C., Heynen S., Haasnoot J., Berkhout B. (2010). A miRNA-tRNA mix-up: tRNA origin of proposed miRNA. RNA Biol..

[101] Lee Y.S., Shibata Y., Malhotra A., Dutta A. (2009). A novel class of small RNAs: tRNA-derived RNA fragments (tRFs). Genes Dev..

[102] Luo S., He F., Luo J., Dou S., Wang Y., Guo A., Lu J. (2018). Drosophila tsRNAs preferentially suppress general translation machinery via antisense pairing and participate in cellular starvation response. Nucleic Acids Res..

[103] Akiyama Y., Kharel P., Abe T., Anderson P., Ivanov P. (2020). Isolation and initial structure-functional characterization of endogenous tRNA-derived stress-induced RNAs. RNA Biol..

[104] Lyons S.M., Gudanis D., Coyne S.M., Gdaniec Z., Ivanov P. (2017). Identification of functional tetramolecular RNA G-quadruplexes derived from transfer RNAs. Nat. Commun..

[105] Zhu L., Ge J., Li T., Shen Y., Guo J. (2019). tRNA-derived fragments and tRNA halves: The new players in cancers. Cancer Lett..

[106] Olvedy M., Scaravilli M., Hoogstrate Y., Visakorpi T., Jenster G., Martens-Uzunova E.S. (2016). A comprehensive repertoire of tRNA-derived fragments in prostate cancer. Oncotarget.

[107] Vojtech L., Woo S., Hughes S., Levy C., Ballweber L., Sauteraud R.P., Strobl J., Westerberg K., Gottardo R., Tewari M. (2014). Exosomes in human semen carry a distinctive repertoire of small non-coding RNAs with potential regulatory functions. Nucleic Acids Res..

[108] Saikia M., Jobava R., Parisien M., Putnam A., Krokowski D., Gao X.H., Guan B.J., Yuan Y., Jankowsky E., Feng Z. (2014). Angiogenin-cleaved tRNA halves interact with cytochrome c, protecting cells from apoptosis during osmotic stress. Mol. Cell Biol..

[109] Zhou K., Diebel K.W., Holy J., Skildum A., Odean E., Hicks D.A., Schotl B., Abrahante J.E., Spillman M.A., Bemis L.T. (2017). A tRNA fragment, tRF5-Glu, regulates BCAR3 expression and proliferation in ovarian cancer cells. Oncotarget.

[110] Balatti V., Nigita G., Veneziano D., Drusco A., Stein G.S., Messier T.L., Farina N.H., Lian J.B., Tomasello L., Liu C.G. (2017). tsRNA signatures in cancer. Proc. Natl. Acad. Sci. USA.

[111] Peng E.Y., Shu Y., Wu Y., Zeng F., Tan S., Deng Y., Deng Y., Chen H., Zhu L., Xu H. (2018). Presence and diagnostic value of circulating tsncRNA for ovarian tumor. Mol. Cancer.

[112] Zhang M., Li F., Wang J., He W., Li Y., Li H., Wei Z., Cao Y. (2019). tRNA-derived fragment tRF-03357 promotes cell proliferation, migration and invasion in high-grade serous ovarian cancer. OncoTargets Ther..

[113] Daly N.L., Arvanitis D.A., Fairley J.A., Gomez-Roman N., Morton J.P., Graham S.V., Spandidos D.A., White R.J. (2005). Deregulation of RNA polymerase III transcription in cervical epithelium in response to high-risk human papillomavirus. Oncogene.

[114] Pekarsky Y., Balatti V., Palamarchuk A., Rizzotto L., Veneziano D., Nigita G., Rassenti L.Z., Pass H.I., Kipps T.J., Liu C.G. (2016). Dysregulation of a family of short noncoding RNAs, tsRNAs, in human cancer. Proc. Natl. Acad. Sci. USA.

[115] Keith B., Simon M.C. (2007). Hypoxia-inducible factors, stem cells, and cancer. Cell.

[116] Magee R.G., Telonis A.G., Loher P., Londin E., Rigoutsos I. (2018). Profiles of miRNA Isoforms and tRNA Fragments in Prostate Cancer. Sci. Rep..

[117] Zhao C., Tolkach Y., Schmidt D., Kristiansen G., Muller S.C., Ellinger J. (2018). 5′-tRNA Halves are Dysregulated in Clear Cell Renal Cell Carcinoma. J. Urol..

[118] Yeri A., Courtright A., Reiman R., Carlson E., Beecroft T., Janss A., Siniard A., Richholt R., Balak C., Rozowsky J. (2017). Total Extracellular Small RNA Profiles from Plasma, Saliva, and Urine of Healthy Subjects. Sci. Rep..

[119] Dhahbi J.M., Spindler S.R., Atamna H., Boffelli D., Martin D.I. (2014). Deep Sequencing of Serum Small RNAs Identifies Patterns of 5′ tRNA Half and YRNA Fragment Expression Associated with Breast Cancer. Biomark. Cancer.

[120] Balatti V., Rizzotto L., Miller C., Palamarchuk A., Fadda P., Pandolfo R., Rassenti L.Z., Hertlein E., Ruppert A.S., Lozanski A. (2015). TCL1 targeting miR-3676 is codeleted with tumor protein p53 in chronic lymphocytic leukemia. Proc. Natl. Acad. Sci. USA.

[121] Lee R.C., Feinbaum R.L., Ambros V. (1993). The C. elegans heterochronic gene lin-4 encodes small RNAs with antisense complementarity to lin-14. Cell.

[122] Bentwich I., Avniel A., Karov Y., Aharonov R., Gilad S., Barad O., Barzilai A., Einat P., Einav U., Meiri E. (2005). Identification of hundreds of conserved and nonconserved human microRNAs. Nat. Genet..

[123] Pasquinelli A.E., Reinhart B.J., Slack F., Martindale M.Q., Kuroda M.I., Maller B., Hayward D.C., Ball E.E., Degnan B., Muller P. (2000). Conservation of the sequence and temporal expression of let-7 heterochronic regulatory RNA. Nature.

[124] Liu H., Lei C., He Q., Pan Z., Xiao D., Tao Y. (2018). Nuclear functions of mammalian MicroRNAs in gene regulation, immunity and cancer. Mol. Cancer.

[125] Rottiers V., Naar A.M. (2012). MicroRNAs in metabolism and metabolic disorders. Nat. Rev. Mol. Cell Biol..

[126] Martinez N.J., Walhout A.J. (2009). The interplay between transcription factors and microRNAs in genome-scale regulatory networks. Bioessays.

[127] Hwang H.W., Wentzel E.A., Mendell J.T. (2007). A hexanucleotide element directs microRNA nuclear import. Science.

[128] O’Brien J., Hayder H., Zayed Y., Peng C. (2018). Overview of MicroRNA Biogenesis, Mechanisms of Actions, and Circulation. Front. Endocrinol..

[129] Ling H., Fabbri M., Calin G.A. (2013). MicroRNAs and other non-coding RNAs as targets for anticancer drug development. Nat. Rev. Drug Discov..

[130] Lee Y., Kim M., Han J., Yeom K.H., Lee S., Baek S.H., Kim V.N. (2004). MicroRNA genes are transcribed by RNA polymerase II. EMBO J..

[131] Han J., Lee Y., Yeom K.H., Kim Y.K., Jin H., Kim V.N. (2004). The Drosha-DGCR8 complex in primary microRNA processing. Genes Dev..

[132] Shukla G.C., Singh J., Barik S. (2011). MicroRNAs: Processing, Maturation, Target Recognition and Regulatory Functions. Mol. Cell Pharmacol..

[133] Li G., Wu X., Qian W., Cai H., Sun X., Zhang W., Tan S., Wu Z., Qian P., Ding K. (2016). CCAR1 5’ UTR as a natural miRancer of miR-1254 overrides tamoxifen resistance. Cell Res..

[134] Brummer A., Hausser J. (2014). MicroRNA binding sites in the coding region of mRNAs: Extending the repertoire of post-transcriptional gene regulation. Bioessays.

[135] Zhang Y., Fan M., Zhang X., Huang F., Wu K., Zhang J., Liu J., Huang Z., Luo H., Tao L. (2014). Cellular microRNAs up-regulate transcription via interaction with promoter TATA-box motifs. RNA.

[136] Huntzinger E., Izaurralde E. (2011). Gene silencing by microRNAs: Contributions of translational repression and mRNA decay. Nat. Rev. Genet..

[137] Peng Y., Croce C.M. (2016). The role of MicroRNAs in human cancer. Signal Transduct. Target. Ther..

[138] Tufekci K.U., Meuwissen R.L., Genc S. (2014). The role of microRNAs in biological processes. Methods Mol. Biol..

[139] Macfarlane L.A., Murphy P.R. (2010). MicroRNA: Biogenesis, Function and Role in Cancer. Curr. Genom..

[140] Volinia S., Calin G.A., Liu C.G., Ambs S., Cimmino A., Petrocca F., Visone R., Iorio M., Roldo C., Ferracin M. (2006). A microRNA expression signature of human solid tumors defines cancer gene targets. Proc. Natl. Acad. Sci. USA.

[141] Kim Y.W., Kim E.Y., Jeon D., Liu J.L., Kim H.S., Choi J.W., Ahn W.S. (2014). Differential microRNA expression signatures and cell type-specific association with Taxol resistance in ovarian cancer cells. Drug Des. Dev. Ther..

[142] Iorio M.V., Croce C.M. (2012). MicroRNA dysregulation in cancer: Diagnostics, monitoring and therapeutics. A comprehensive review. EMBO Mol. Med..

[143] Calin G.A., Sevignani C., Dumitru C.D., Hyslop T., Noch E., Yendamuri S., Shimizu M., Rattan S., Bullrich F., Negrini M. (2004). Human microRNA genes are frequently located at fragile sites and genomic regions involved in cancers. Proc. Natl. Acad. Sci. USA.

[144] Krol J., Loedige I., Filipowicz W. (2010). The widespread regulation of microRNA biogenesis, function and decay. Nat. Rev. Genet..

[145] Huang Y., Shen X.J., Zou Q., Wang S.P., Tang S.M., Zhang G.Z. (2011). Biological functions of microRNAs: A review. J. Physiol. Biochem..

[146] Perera R.J., Ray A. (2007). MicroRNAs in the search for understanding human diseases. BioDrugs.

[147] Wang Q., Xu K., Tong Y., Dai X., Xu T., He D., Ying J. (2020). Novel miRNA markers for the diagnosis and prognosis of endometrial cancer. J. Cell Mol. Med..

[148] Xu X., Liu T., Wang Y., Fu J., Yang Q., Wu J., Zhou H. (2019). miRNA-mRNA Associated with Survival in Endometrial Cancer. Front. Genet..

[149] Chen S.N., Chang R., Lin L.T., Chern C.U., Tsai H.W., Wen Z.H., Li Y.H., Li C.J., Tsui K.H. (2019). MicroRNA in Ovarian Cancer: Biology, Pathogenesis, and Therapeutic Opportunities. Int. J. Environ. Res. Public Health.

[150] Banno K., Iida M., Yanokura M., Kisu I., Iwata T., Tominaga E., Tanaka K., Aoki D. (2014). MicroRNA in cervical cancer: OncomiRs and tumor suppressor miRs in diagnosis and treatment. Sci. World J..

[151] Wren J.D., Bekeredjian R., Stewart J.A., Shohet R.V., Garner H.R. (2004). Knowledge discovery by automated identification and ranking of implicit relationships. Bioinformatics.

[152] Chang L., Zhou G., Soufan O., Xia J. (2020). miRNet 2.0: Network-based visual analytics for miRNA functional analysis and systems biology. Nucleic Acids Res..

[153] Iorio M.V., Visone R., Di Leva G., Donati V., Petrocca F., Casalini P., Taccioli C., Volinia S., Liu C.G., Alder H. (2007). MicroRNA signatures in human ovarian cancer. Cancer Res..

[154] Dwivedi S.K., Mustafi S.B., Mangala L.S., Jiang D., Pradeep S., Rodriguez-Aguayo C., Ling H., Ivan C., Mukherjee P., Calin G.A. (2016). Therapeutic evaluation of microRNA-15a and microRNA-16 in ovarian cancer. Oncotarget.

[155] Bhattacharya R., Nicoloso M., Arvizo R., Wang E., Cortez A., Rossi S., Calin G.A., Mukherjee P. (2009). MiR-15a and MiR-16 control Bmi-1 expression in ovarian cancer. Cancer Res..

[156] Rao G., Dwivedi S.K.D., Zhang Y., Dey A., Shameer K., Karthik R., Srikantan S., Hossen M.N., Wren J.D., Madesh M. (2020). MicroRNA-195 controls MICU1 expression and tumor growth in ovarian cancer. EMBO Rep..

[157] Vang S., Wu H.T., Fischer A., Miller D.H., MacLaughlan S., Douglass E., Comisar L., Steinhoff M., Collins C., Smith P.J. (2013). Identification of ovarian cancer metastatic miRNAs. PLoS ONE.

[158] Hu X., Macdonald D.M., Huettner P.C., Feng Z., El Naqa I.M., Schwarz J.K., Mutch D.G., Grigsby P.W., Powell S.N., Wang X. (2009). A miR-200 microRNA cluster as prognostic marker in advanced ovarian cancer. Gynecol. Oncol..

[159] Zavesky L., Jandakova E., Turyna R., Langmeierova L., Weinberger V., Zaveska Drabkova L., Hulkova M., Horinek A., Duskova D., Feyereisl J. (2015). Evaluation of Cell-Free Urine microRNAs Expression for the Use in Diagnosis of Ovarian and Endometrial Cancers. A Pilot Study. Pathol. Oncol. Res..

[160] Langhe R., Norris L., Saadeh F.A., Blackshields G., Varley R., Harrison A., Gleeson N., Spillane C., Martin C., O’Donnell D.M. (2015). A novel serum microRNA panel to discriminate benign from malignant ovarian disease. Cancer Lett..

[161] Shapira I., Oswald M., Lovecchio J., Khalili H., Menzin A., Whyte J., Dos Santos L., Liang S., Bhuiya T., Keogh M. (2014). Circulating biomarkers for detection of ovarian cancer and predicting cancer outcomes. Br. J. Cancer.

[162] Zheng H., Zhang L., Zhao Y., Yang D., Song F., Wen Y., Hao Q., Hu Z., Zhang W., Chen K. (2013). Plasma miRNAs as diagnostic and prognostic biomarkers for ovarian cancer. PLoS ONE.

[163] Chung Y.W., Bae H.S., Song J.Y., Lee J.K., Lee N.W., Kim T., Lee K.W. (2013). Detection of microRNA as novel biomarkers of epithelial ovarian cancer from the serum of ovarian cancer patients. Int. J. Gynecol. Cancer.

[164] Hausler S.F., Keller A., Chandran P.A., Ziegler K., Zipp K., Heuer S., Krockenberger M., Engel J.B., Honig A., Scheffler M. (2010). Whole blood-derived miRNA profiles as potential new tools for ovarian cancer screening. Br. J. Cancer.

[165] Wyman S.K., Parkin R.K., Mitchell P.S., Fritz B.R., O’Briant K., Godwin A.K., Urban N., Drescher C.W., Knudsen B.S., Tewari M. (2009). Repertoire of microRNAs in epithelial ovarian cancer as determined by next generation sequencing of small RNA cDNA libraries. PLoS ONE.

[166] Merritt W.M., Lin Y.G., Han L.Y., Kamat A.A., Spannuth W.A., Schmandt R., Urbauer D., Pennacchio L.A., Cheng J.F., Nick A.M. (2008). Dicer, Drosha, and outcomes in patients with ovarian cancer. N. Engl. J. Med..

[167] Nam E.J., Yoon H., Kim S.W., Kim H., Kim Y.T., Kim J.H., Kim J.W., Kim S. (2008). MicroRNA expression profiles in serous ovarian carcinoma. Clin. Cancer Res..

[168] Kan C.W., Hahn M.A., Gard G.B., Maidens J., Huh J.Y., Marsh D.J., Howell V.M. (2012). Elevated levels of circulating microRNA-200 family members correlate with serous epithelial ovarian cancer. BMC Cancer.

[169] Hanna J., Hossain G.S., Kocerha J. (2019). The Potential for microRNA Therapeutics and Clinical Research. Front. Genet..

[170] Bonneau E., Neveu B., Kostantin E., Tsongalis G.J., De Guire V. (2019). How close are miRNAs from clinical practice? A perspective on the diagnostic and therapeutic market. EJIFCC.

[171] Huang H.Y., Lin Y.C., Li J., Huang K.Y., Shrestha S., Hong H.C., Tang Y., Chen Y.G., Jin C.N., Yu Y. (2020). miRTarBase 2020: Updates to the experimentally validated microRNA-target interaction database. Nucleic Acids Res..

[172] Bertucci A., Kim K.H., Kang J., Zuidema J.M., Lee S.H., Kwon E.J., Kim D., Howell S.B., Ricci F., Ruoslahti E. (2019). Tumor-Targeting, MicroRNA-Silencing Porous Silicon Nanoparticles for Ovarian Cancer Therapy. ACS Appl. Mater. Interfaces.

[173] Kim S., Choi M.C., Jeong J.Y., Hwang S., Jung S.G., Joo W.D., Park H., Song S.H., Lee C., Kim T.H. (2019). Serum exosomal miRNA-145 and miRNA-200c as promising biomarkers for preoperative diagnosis of ovarian carcinomas. J. Cancer.

[174] Pan C., Stevic I., Muller V., Ni Q., Oliveira-Ferrer L., Pantel K., Schwarzenbach H. (2018). Exosomal microRNAs as tumor markers in epithelial ovarian cancer. Mol. Oncol..

[175] Ma N., Li S., Zhang Q., Wang H., Qin H., Wang S. (2018). Long non-coding RNA GAS5 inhibits ovarian cancer cell proliferation via the control of microRNA-21 and SPRY2 expression. Exp. Ther. Med..

[176] Echevarria-Vargas I.M., Valiyeva F., Vivas-Mejia P.E. (2014). Upregulation of miR-21 in cisplatin resistant ovarian cancer via JNK-1/c-Jun pathway. PLoS ONE.

[177] Wei J., Zhang L., Li J., Zhu S., Tai M., Mason C.W., Chapman J.A., Reynolds E.A., Weiner C.P., Zhou H.H. (2017). MicroRNA-205 promotes cell invasion by repressing TCF21 in human ovarian cancer. J. Ovarian Res..

[178] Taylor D.D., Gercel-Taylor C. (2008). MicroRNA signatures of tumor-derived exosomes as diagnostic biomarkers of ovarian cancer. Gynecol. Oncol..

[179] Marton E., Lukacs J., Penyige A., Janka E., Hegedus L., Soltesz B., Mehes G., Poka R., Nagy B., Szilagyi M. (2019). Circulating epithelial-mesenchymal transition-associated miRNAs are promising biomarkers in ovarian cancer. J. Biotechnol..

[180] Shi M., Mu Y., Zhang H., Liu M., Wan J., Qin X., Li C. (2018). MicroRNA-200 and microRNA-30 family as prognostic molecular signatures in ovarian cancer: A meta-analysis. Medicine.

[181] Yang H., Kong W., He L., Zhao J.J., O’Donnell J.D., Wang J., Wenham R.M., Coppola D., Kruk P.A., Nicosia S.V. (2008). MicroRNA expression profiling in human ovarian cancer: miR-214 induces cell survival and cisplatin resistance by targeting PTEN. Cancer Res..

[182] Sun N., Zhang Q., Xu C., Zhao Q., Ma Y., Lu X., Wang L., Li W. (2014). Molecular regulation of ovarian cancer cell invasion. Tumour Biol..

[183] Mak C.S., Yung M.M., Hui L.M., Leung L.L., Liang R., Chen K., Liu S.S., Qin Y., Leung T.H., Lee K.F. (2017). MicroRNA-141 enhances anoikis resistance in metastatic progression of ovarian cancer through targeting KLF12/Sp1/survivin axis. Mol. Cancer.

[184] Wang L., Zhu M.J., Ren A.M., Wu H.F., Han W.M., Tan R.Y., Tu R.Q. (2014). A ten-microRNA signature identified from a genome-wide microRNA expression profiling in human epithelial ovarian cancer. PLoS ONE.

[185] Xu X., Ayub B., Liu Z., Serna V.A., Qiang W., Liu Y., Hernando E., Zabludoff S., Kurita T., Kong B. (2014). Anti-miR182 reduces ovarian cancer burden, invasion, and metastasis: An in vivo study in orthotopic xenografts of nude mice. Mol. Cancer Ther..

[186] Wilczynski M., Zytko E., Szymanska B., Dzieniecka M., Nowak M., Danielska J., Stachowiak G., Wilczynski J.R. (2017). Expression of miR-146a in patients with ovarian cancer and its clinical significance. Oncol. Lett..

[187] Li L., Huang K., You Y., Fu X., Hu L., Song L., Meng Y. (2014). Hypoxia-induced miR-210 in epithelial ovarian cancer enhances cancer cell viability via promoting proliferation and inhibiting apoptosis. Int. J. Oncol..

[188] Yang L.Y., Wang H.J., Jia X.B., Wang X., Luo J., Zhang X.Y. (2012). Expression of miR-130a in cisplatin resistant cell lines of ovarian cancer. Sichuan Da Xue Xue Bao Yi Xue Ban.

[189] Gadducci A., Sergiampietri C., Lanfredini N., Guiggi I. (2014). Micro-RNAs and ovarian cancer: The state of art and perspectives of clinical research. Gynecol. Endocrinol..

[190] Chen L., Zhang F., Sheng X.G., Zhang S.Q., Chen Y.T., Liu B.W. (2016). MicroRNA-106a regulates phosphatase and tensin homologue expression and promotes the proliferation and invasion of ovarian cancer cells. Oncol. Rep..

[191] Chen X., Mangala L.S., Mooberry L., Bayraktar E., Dasari S.K., Ma S., Ivan C., Court K.A., Rodriguez-Aguayo C., Bayraktar R. (2019). Identifying and targeting angiogenesis-related microRNAs in ovarian cancer. Oncogene.

[192] Tian H., Hou L., Xiong Y.M., Huang J.X., Zhang W.H., Pan Y.Y., Song X.R. (2016). miR-132 targeting E2F5 suppresses cell proliferation, invasion, migration in ovarian cancer cells. Am. J. Transl. Res..

[193] Yang L., Li N., Wang H., Jia X., Wang X., Luo J. (2012). Altered microRNA expression in cisplatin-resistant ovarian cancer cells and upregulation of miR-130a associated with MDR1/P-glycoprotein-mediated drug resistance. Oncol. Rep..

[194] Liu Y., Han S., Li Y., Liu Y., Zhang D., Li Y., Zhang J. (2017). MicroRNA-20a contributes to cisplatin-resistance and migration of OVCAR3 ovarian cancer cell line. Oncol. Lett..

[195] Chen H., Zhang L., Zhang L., Du J., Wang H., Wang B. (2016). MicroRNA-183 correlates cancer prognosis, regulates cancer proliferation and bufalin sensitivity in epithelial ovarian cancer. Am. J. Transl. Res..

[196] Zhang S., Zhang J.Y., Lu L.J., Wang C.H., Wang L.H. (2017). MiR-630 promotes epithelial ovarian cancer proliferation and invasion via targeting KLF6. Eur. Rev. Med. Pharmacol. Sci..

[197] Fang G., Liu J., Wang Q., Huang X., Yang R., Pang Y., Yang M. (2017). MicroRNA-223-3p Regulates Ovarian Cancer Cell Proliferation and Invasion by Targeting SOX11 Expression. Int. J. Mol. Sci..

[198] Shi W., Wang X., Ruan L., Fu J., Liu F., Qu J. (2017). MiR-200a promotes epithelial-mesenchymal transition of endometrial cancer cells by negatively regulating FOXA2 expression. Pharmazie.

[199] Meng X., Muller V., Milde-Langosch K., Trillsch F., Pantel K., Schwarzenbach H. (2016). Circulating Cell-Free miR-373, miR-200a, miR-200b and miR-200c in Patients with Epithelial Ovarian Cancer. Adv. Exp. Med. Biol..

[200] Loginov V.I., Burdennyy A.M., Filippova E.A., Pronina I.V., Kazubskaya T.P., Kushlinsky D.N., Ermilova V.D., Rykov S.V., Khodyrev D.S., Braga E.A. (2018). Hypermethylation of miR-107, miR-130b, miR-203a, miR-1258 Genes Associated with Ovarian Cancer Development and Metastasis. Mol. Biol..

[201] Nakano H., Yamada Y., Miyazawa T., Yoshida T. (2013). Gain-of-function microRNA screens identify miR-193a regulating proliferation and apoptosis in epithelial ovarian cancer cells. Int. J. Oncol..

[202] Dong M., Yang P., Hua F. (2015). MiR-191 modulates malignant transformation of endometriosis through regulating TIMP3. Med. Sci. Monit..

[203] Zhou Y., Wang M., Wu J., Jie Z., Chang S., Shuang T. (2015). The clinicopathological significance of miR-1307 in chemotherapy resistant epithelial ovarian cancer. J. Ovarian Res..

[204] Liu W., Lv C., Zhang B., Zhou Q., Cao Z. (2017). MicroRNA-27b functions as a new inhibitor of ovarian cancer-mediated vasculogenic mimicry through suppression of VE-cadherin expression. RNA.

[205] Chao A., Lin C.Y., Lee Y.S., Tsai C.L., Wei P.C., Hsueh S., Wu T.I., Tsai C.N., Wang C.J., Chao A.S. (2012). Regulation of ovarian cancer progression by microRNA-187 through targeting Disabled homolog-2. Oncogene.

[206] Wang Z., Liu Y., Wang M., Zhao J. (2020). Effects of miR-492 on migration, invasion, EMT and prognosis in ovarian cancer by targeting SOX7. J. BUON.

[207] Liao Y., Deng Y., Liu J., Ye Z., You Z., Yao S., He S. (2016). MiR-760 overexpression promotes proliferation in ovarian cancer by downregulation of PHLPP2 expression. Gynecol. Oncol..

[208] Zhu T., Yuan J., Wang Y., Gong C., Xie Y., Li H. (2015). MiR-661 contributed to cell proliferation of human ovarian cancer cells by repressing INPP5J expression. Biomed. Pharmacother..

[209] Ruan L., Xie Y., Liu F., Chen X. (2018). Serum miR-1181 and miR-4314 associated with ovarian cancer: MiRNA microarray data analysis for a pilot study. Eur. J. Obstet. Gynecol. Reprod. Biol..

[210] Filippova E.A., Loginov V.I., Burdennyi A.M., Braga E.A., Pronina I.V., Kazubskaya T.P., Kushlinskii D.N., Utkin D.O., Fridman M.V., Khodyrev D.S. (2019). Hypermethylated Genes of MicroRNA in Ovarian Carcinoma: Metastasis Prediction Marker Systems. Bull. Exp. Biol. Med..

[211] Rashed M.H., Kanlikilicer P., Rodriguez-Aguayo C., Pichler M., Bayraktar R., Bayraktar E., Ivan C., Filant J., Silva A., Aslan B. (2017). Exosomal miR-940 maintains SRC-mediated oncogenic activity in cancer cells: A possible role for exosomal disposal of tumor suppressor miRNAs. Oncotarget.

[212] Kobayashi M., Sawada K., Nakamura K., Yoshimura A., Miyamoto M., Shimizu A., Ishida K., Nakatsuka E., Kodama M., Hashimoto K. (2018). Exosomal miR-1290 is a potential biomarker of high-grade serous ovarian carcinoma and can discriminate patients from those with malignancies of other histological types. J. Ovarian Res..

[213] Chang H., Zhou X., Wang Z.N., Song Y.X., Zhao F., Gao P., Chiang Y., Xu H.M. (2012). Increased expression of miR-148b in ovarian carcinoma and its clinical significance. Mol. Med. Rep..

[214] Ying X., Li-Ya Q., Feng Z., Yin W., Ji-Hong L. (2015). MiR-939 promotes the proliferation of human ovarian cancer cells by repressing APC2 expression. Biomed. Pharmacother..

[215] Zhang X., Liu J., Zang D., Wu S., Liu A., Zhu J., Wu G., Li J., Jiang L. (2015). Upregulation of miR-572 transcriptionally suppresses SOCS1 and p21 and contributes to human ovarian cancer progression. Oncotarget.

[216] Gu Z.W., He Y.F., Wang W.J., Tian Q., Di W. (2019). MiR-1180 from bone marrow-derived mesenchymal stem cells induces glycolysis and chemoresistance in ovarian cancer cells by upregulating the Wnt signaling pathway. J. Zhejiang Univ. Sci..

[217] Han X., Zhang Y., Wang D., Fu X., Li M., Wang A. (2017). Upregulation of microRNA-18b induces phosphatase and tensin homolog to accelerate the migration and invasion abilities of ovarian cancer. Oncol. Lett..

[218] Teng C., Zheng H. (2017). Low expression of microRNA-1908 predicts a poor prognosis for patients with ovarian cancer. Oncol. Lett..

[219] Li X., Lin S., Mo Z., Jiang J., Tang H., Wu C., Song J. (2020). CircRNA_100395 inhibits cell proliferation and metastasis in ovarian cancer via regulating miR-1228/p53/epithelial-mesenchymal transition (EMT) axis. J. Cancer.

[220] Duan Y., Dong Y., Dang R., Hu Z., Yang Y., Hu Y., Cheng J. (2018). MiR-122 inhibits epithelial mesenchymal transition by regulating P4HA1 in ovarian cancer cells. Cell Biol. Int..

[221] Chong G.O., Jeon H.S., Han H.S., Son J.W., Lee Y.H., Hong D.G., Park H.J., Lee Y.S., Cho Y.L. (2017). Overexpression of microRNA-196b Accelerates Invasiveness of Cancer Cells in Recurrent Epithelial Ovarian Cancer Through Regulation of Homeobox A9. Cancer Genom. Proteom..

[222] Zhao W., Han T., Li B., Ma Q., Yang P., Li H. (2019). miR-552 promotes ovarian cancer progression by regulating PTEN pathway. J. Ovarian Res..

[223] Wu G., Liu A., Zhu J., Lei F., Wu S., Zhang X., Ye L., Cao L., He S. (2015). MiR-1207 overexpression promotes cancer stem cell-like traits in ovarian cancer by activating the Wnt/beta-catenin signaling pathway. Oncotarget.

[224] Shao L., Shen Z., Qian H., Zhou S., Chen Y. (2017). Knockdown of miR-629 Inhibits Ovarian Cancer Malignant Behaviors by Targeting Testis-Specific Y-Like Protein 5. DNA Cell Biol..

[225] Guan R., Cai S., Sun M., Xu M. (2017). Upregulation of miR-520b promotes ovarian cancer growth. Oncol. Lett..

[226] Hu Y., Zhang Q., Cui J., Liao Z.J., Jiao M., Zhang Y.B., Guo Y.H., Gao Y.M. (2019). Oncogene miR-934 promotes ovarian cancer cell proliferation and inhibits cell apoptosis through targeting BRMS1L. Eur. Rev. Med. Pharmacol. Sci..

[227] Zhihong Z., Rubin C., Liping L., Anpeng M., Hui G., Yanting W., Zhenxiu S. (2020). MicroRNA-1179 regulates proliferation and chemosensitivity of human ovarian cancer cells by targeting the PTEN-mediated PI3K/AKT signaling pathway. Arch. Med. Sci..

[228] Liu Y., Niu Z., Lin X., Tian Y. (2017). MiR-216b increases cisplatin sensitivity in ovarian cancer cells by targeting PARP1. Cancer Gene Ther..

[229] Liu L., Ning Y., Yi J., Yuan J., Fang W., Lin Z., Zeng Z. (2020). miR-6089/MYH9/beta-catenin/c-Jun negative feedback loop inhibits ovarian cancer carcinogenesis and progression. Biomed. Pharmacother..

[230] Chen W., Huang L., Hao C., Zeng W., Luo X., Li X., Zhou L., Jiang S., Chen Z., He Y. (2016). MicroRNA-155 promotes apoptosis in SKOV3, A2780, and primary cultured ovarian cancer cells. Tumour Biol..

[231] Sun J., Cai X., Yung M.M., Zhou W., Li J., Zhang Y., Li Z., Liu S.S., Cheung A.N.Y., Ngan H.Y.S. (2019). miR-137 mediates the functional link between c-Myc and EZH2 that regulates cisplatin resistance in ovarian cancer. Oncogene.

[232] Li X., Chen W., Zeng W., Wan C., Duan S., Jiang S. (2017). microRNA-137 promotes apoptosis in ovarian cancer cells via the regulation of XIAP. Br. J. Cancer.

[233] Zhang L., Li Z., Gai F., Wang Y. (2015). MicroRNA-137 suppresses tumor growth in epithelial ovarian cancer in vitro and in vivo. Mol. Med. Rep..

[234] Wan W.N., Zhang Y.Q., Wang X.M., Liu Y.J., Zhang Y.X., Que Y.H., Zhao W.J., Li P. (2014). Down-regulated miR-22 as predictive biomarkers for prognosis of epithelial ovarian cancer. Diagn. Pathol..

[235] Yang D., Sun Y., Hu L., Zheng H., Ji P., Pecot C.V., Zhao Y., Reynolds S., Cheng H., Rupaimoole R. (2013). Integrated analyses identify a master microRNA regulatory network for the mesenchymal subtype in serous ovarian cancer. Cancer Cell.

[236] Su L., Liu M. (2018). Correlation analysis on the expression levels of microRNA-23a and microRNA-23b and the incidence and prognosis of ovarian cancer. Oncol. Lett..

[237] Wang X., Wang H.K., Li Y., Hafner M., Banerjee N.S., Tang S., Briskin D., Meyers C., Chow L.T., Xie X. (2014). microRNAs are biomarkers of oncogenic human papillomavirus infections. Proc. Natl. Acad. Sci. USA.

[238] Zhou X., Zhao F., Wang Z.N., Song Y.X., Chang H., Chiang Y., Xu H.M. (2012). Altered expression of miR-152 and miR-148a in ovarian cancer is related to cell proliferation. Oncol. Rep..

[239] Ye Z., Zhao L., Li J., Chen W., Li X. (2015). miR-30d Blocked Transforming Growth Factor beta1-Induced Epithelial-Mesenchymal Transition by Targeting Snail in Ovarian Cancer Cells. Int. J. Gynecol. Cancer.

[240] Gong L., Wang C., Gao Y., Wang J. (2016). Decreased expression of microRNA-148a predicts poor prognosis in ovarian cancer and associates with tumor growth and metastasis. Biomed. Pharmacother..

[241] Paudel D., Zhou W., Ouyang Y., Dong S., Huang Q., Giri R., Wang J., Tong X. (2016). MicroRNA-130b functions as a tumor suppressor by regulating RUNX3 in epithelial ovarian cancer. Gene.

[242] Xiang G., Cheng Y. (2018). MiR-126-3p inhibits ovarian cancer proliferation and invasion via targeting PLXNB2. Reprod. Biol..

[243] Li H., Xu Y., Qiu W., Zhao D., Zhang Y. (2015). Tissue miR-193b as a Novel Biomarker for Patients with Ovarian Cancer. Med. Sci. Monit..

[244] Liu P., Qi X., Bian C., Yang F., Lin X., Zhou S., Xie C., Zhao X., Yi T. (2017). MicroRNA-18a inhibits ovarian cancer growth via directly targeting TRIAP1 and IPMK. Oncol. Lett..

[245] Jiang H., Qu L., Wang Y., Cong J., Wang W., Yang X. (2014). miR-99a promotes proliferation targeting FGFR3 in human epithelial ovarian cancer cells. Biomed. Pharmacother..

[246] Wang W., Ren F., Wu Q., Jiang D., Li H., Peng Z., Wang J., Shi H. (2014). MicroRNA-497 inhibition of ovarian cancer cell migration and invasion through targeting of SMAD specific E3 ubiquitin protein ligase 1. Biochem. Biophys. Res. Commun..

[247] Yan J., Jiang J.Y., Meng X.N., Xiu Y.L., Zong Z.H. (2016). MiR-23b targets cyclin G1 and suppresses ovarian cancer tumorigenesis and progression. J. Exp. Clin. Cancer Res..

[248] Wang L., Wang B., Fang M., Guo F., Cui M. (2014). Identification of microRNAs and target genes involved in serous ovarian carcinoma and their influence on survival. Eur. J. Gynaecol. Oncol..

[249] Lee M., Kim E.J., Jeon M.J. (2016). MicroRNAs 125a and 125b inhibit ovarian cancer cells through post-transcriptional inactivation of EIF4EBP1. Oncotarget.

[250] Shuang T., Wang M., Shi C., Zhou Y., Wang D. (2015). Down-regulated expression of miR-134 contributes to paclitaxel resistance in human ovarian cancer cells. FEBS Lett..

[251] Li J., Zhang S., Zou Y., Wu L., Pei M., Jiang Y. (2020). miR-145 promotes miR-133b expression through c-myc and DNMT3A-mediated methylation in ovarian cancer cells. J. Cell Physiol..

[252] Sun L., Zhai R., Zhang L., Zhao S. (2018). MicroRNA-149 suppresses the proliferation and increases the sensitivity of ovarian cancer cells to cisplatin by targeting X-linked inhibitor of apoptosis. Oncol. Lett..

[253] Lu J., Zhang W., Ding Y., Li X., Song J. (2019). Expression of miR-26b in ovarian carcinoma tissues and its correlation with clinicopathology. Oncol. Lett..

[254] Kim T.H., Jeong J.Y., Park J.Y., Kim S.W., Heo J.H., Kang H., Kim G., An H.J. (2017). miR-150 enhances apoptotic and anti-tumor effects of paclitaxel in paclitaxel-resistant ovarian cancer cells by targeting Notch3. Oncotarget.

[255] Jin M., Yang Z., Ye W., Xu H., Hua X. (2014). MicroRNA-150 predicts a favorable prognosis in patients with epithelial ovarian cancer, and inhibits cell invasion and metastasis by suppressing transcriptional repressor ZEB1. PLoS ONE.

[256] Li J., Li D., Zhang W. (2016). Tumor suppressor role of miR-217 in human epithelial ovarian cancer by targeting IGF1R. Oncol. Rep..

[257] Theriault B.L., Basavarajappa H.D., Lim H., Pajovic S., Gallie B.L., Corson T.W. (2014). Transcriptional and epigenetic regulation of KIF14 overexpression in ovarian cancer. PLoS ONE.

[258] Liu X., Ma L., Rao Q., Mao Y., Xin Y., Xu H., Li C., Wang X. (2015). MiR-1271 Inhibits Ovarian Cancer Growth by Targeting Cyclin G1. Med. Sci. Monit..

[259] Fu X., Cui Y., Yang S., Xu Y., Zhang Z. (2016). MicroRNA-613 inhibited ovarian cancer cell proliferation and invasion by regulating KRAS. Tumour Biol..

[260] Liu R., Liu F., Li L., Sun M., Chen K. (2015). MiR-498 regulated FOXO3 expression and inhibited the proliferation of human ovarian cancer cells. Biomed. Pharmacother..

[261] Zhou Q.H., Zhao Y.M., Jia L.L., Zhang Y. (2017). Mir-595 is a significant indicator of poor patient prognosis in epithelial ovarian cancer. Eur. Rev. Med. Pharmacol. Sci..

[262] Li Y., Yao L., Liu F., Hong J., Chen L., Zhang B., Zhang W. (2014). Characterization of microRNA expression in serous ovarian carcinoma. Int. J. Mol. Med..

[263] Zhang S., Lu Z., Unruh A.K., Ivan C., Baggerly K.A., Calin G.A., Li Z., Bast R.C., Le X.F. (2015). Clinically relevant microRNAs in ovarian cancer. Mol. Cancer Res..

[264] Xiao F., Xiao S., Xue M. (2019). miR-139 Controls Viability of Ovarian Cancer Cells Through Apoptosis Induction And Exosome Shedding Inhibition By Targeting ATP7A. OncoTargets Ther..

[265] MacLean J.A., King M.L., Okuda H., Hayashi K. (2016). WNT7A Regulation by miR-15b in Ovarian Cancer. PLoS ONE.

[266] Yao L., Wang L., Li F., Gao X., Wei X., Liu Z. (2015). MiR181c inhibits ovarian cancer metastasis and progression by targeting PRKCD expression. Int. J. Clin. Exp. Med..

[267] Jeong J.Y., Kang H., Kim T.H., Kim G., Heo J.H., Kwon A.Y., Kim S., Jung S.G., An H.J. (2017). MicroRNA-136 inhibits cancer stem cell activity and enhances the anti-tumor effect of paclitaxel against chemoresistant ovarian cancer cells by targeting Notch3. Cancer Lett..

[268] Guan X., Zong Z.H., Chen S., Sang X.B., Wu D.D., Wang L.L., Liu Y., Zhao Y. (2017). The role of miR-372 in ovarian carcinoma cell proliferation. Gene.

[269] Hou X.S., Han C.Q., Zhang W. (2018). MiR-1182 inhibited metastasis and proliferation of ovarian cancer by targeting hTERT. Eur. Rev. Med. Pharmacol. Sci..

[270] Bai L., Wang H., Wang A.H., Zhang L.Y., Bai J. (2017). MicroRNA-532 and microRNA-3064 inhibit cell proliferation and invasion by acting as direct regulators of human telomerase reverse transcriptase in ovarian cancer. PLoS ONE.

[271] Pang Y., Mao H., Shen L., Zhao Z., Liu R., Liu P. (2014). MiR-519d represses ovarian cancer cell proliferation and enhances cisplatin-mediated cytotoxicity in vitro by targeting XIAP. OncoTargets Ther..

[272] Zhang W., Zeng Q., Ban Z., Cao J., Chu T., Lei D., Liu C., Guo W., Zeng X. (2018). Effects of let-7c on the proliferation of ovarian carcinoma cells by targeted regulation of CDC25a gene expression. Oncol. Lett..

[273] Hong L., Wang Y., Chen W., Yang S. (2018). MicroRNA-508 suppresses epithelial-mesenchymal transition, migration, and invasion of ovarian cancer cells through the MAPK1/ERK signaling pathway. J. Cell Biochem..

[274] Xing F., Wang S., Zhou J. (2019). The Expression of MicroRNA-598 Inhibits Ovarian Cancer Cell Proliferation and Metastasis by Targeting URI. Mol. Ther. Oncolytics.

[275] Kanlikilicer P., Rashed M.H., Bayraktar R., Mitra R., Ivan C., Aslan B., Zhang X., Filant J., Silva A.M., Rodriguez-Aguayo C. (2016). Ubiquitous Release of Exosomal Tumor Suppressor miR-6126 from Ovarian Cancer Cells. Cancer Res..

[276] Guo T.Y., Xu H.Y., Chen W.J., Wu M.X., Dai X. (2018). Downregulation of miR-1294 associates with prognosis and tumor progression in epithelial ovarian cancer. Eur. Rev. Med. Pharmacol. Sci..

[277] Xie W., Shui C., Fang X., Peng Y., Qin L. (2020). miR-197-3p reduces epithelial-mesenchymal transition by targeting ABCA7 in ovarian cancer cells. 3 Biotech.

[278] Pan C., Wang D., Zhang Y., Yu W. (2016). MicroRNA-1284 Inhibits Cell Viability and Induces Apoptosis of Ovarian Cancer Cell Line OVCAR3. Oncol. Res..

[279] Bi L., Yang Q., Yuan J., Miao Q., Duan L., Li F., Wang S. (2016). MicroRNA-127-3p acts as a tumor suppressor in epithelial ovarian cancer by regulating the BAG5 gene. Oncol. Rep..

[280] Zhang M., Xia B., Xu Y., Zhang Y., Xu J., Lou G. (2019). Circular RNA (hsa_circ_0051240) promotes cell proliferation, migration and invasion in ovarian cancer through miR-637/KLK4 axis. Artif. Cells Nanomed. Biotechnol..

[281] Yang C., Li B., Yu J., Yang F., Cai K., Chen Z. (2018). Ultrasound microbubbles mediated miR-let-7b delivery into CD133 (+) ovarian cancer stem cells. Biosci. Rep..

[282] Zhang L., Wang Y.H., Wang L. (2019). MiRNA-8073 targets ZnT1 to inhibit malignant progression of ovarian cancer. Eur. Rev. Med. Pharmacol. Sci..

[283] Chirshev E., Oberg K.C., Ioffe Y.J., Unternaehrer J.J. (2019). Let-7 as biomarker, prognostic indicator, and therapy for precision medicine in cancer. Clin. Transl. Med..

[284] Chen Y.N., Ren C.C., Yang L., Nai M.M., Xu Y.M., Zhang F., Liu Y. (2019). MicroRNA let7d5p rescues ovarian cancer cell apoptosis and restores chemosensitivity by regulating the p53 signaling pathway via HMGA1. Int. J. Oncol..

[285] Braga E.A., Loginov V.I., Burdennyi A.M., Filippova E.A., Pronina I.V., Kurevlev S.V., Kazubskaya T.P., Kushlinskii D.N., Utkin D.O., Ermilova V.D. (2018). Five Hypermethylated MicroRNA Genes as Potential Markers of Ovarian Cancer. Bull. Exp. Biol. Med..

[286] Lu J., Wang L., Chen W., Wang Y., Zhen S., Chen H., Cheng J., Zhou Y., Li X., Zhao L. (2019). miR-603 targeted hexokinase-2 to inhibit the malignancy of ovarian cancer cells. Arch. Biochem. Biophys..

[287] Wu D.D., Li X.S., Meng X.N., Yan J., Zong Z.H. (2016). MicroRNA-873 mediates multidrug resistance in ovarian cancer cells by targeting ABCB1. Tumour Biol..

[288] Sun X., Cui M., Zhang A., Tong L., Wang K., Li K., Wang X., Sun Z., Zhang H. (2016). MiR-548c impairs migration and invasion of endometrial and ovarian cancer cells via downregulation of Twist. J. Exp. Clin. Cancer Res..

[289] Li S., Xie Y., Zhang W., Gao J., Wang M., Zheng G., Yin X., Xia H., Tao X. (2015). Interferon alpha-inducible protein 27 promotes epithelial-mesenchymal transition and induces ovarian tumorigenicity and stemness. J. Surg. Res..

[290] Imam J.S., Plyler J.R., Bansal H., Prajapati S., Bansal S., Rebeles J., Chen H.I., Chang Y.F., Panneerdoss S., Zoghi B. (2012). Genomic loss of tumor suppressor miRNA-204 promotes cancer cell migration and invasion by activating AKT/mTOR/Rac1 signaling and actin reorganization. PLoS ONE.

[291] Tan G., Cao X., Dai Q., Zhang B., Huang J., Xiong S., Zhang Y., Chen W., Yang J., Li H. (2015). A novel role for microRNA-129-5p in inhibiting ovarian cancer cell proliferation and survival via direct suppression of transcriptional co-activators YAP and TAZ. Oncotarget.

[292] Dasari S., Pandhiri T., Grassi T., Visscher D.W., Multinu F., Agarwal K., Mariani A., Shridhar V., Mitra A.K. (2020). Signals from the Metastatic Niche Regulate Early and Advanced Ovarian Cancer Metastasis through miR-4454 Downregulation. Mol. Cancer Res..

[293] Zhang J., Quan L.N., Meng Q., Wang H.Y., Wang J., Yu P., Fu J.T., Li Y.J., Chen J., Cheng H. (2020). miR-548e Sponged by ZFAS1 Regulates Metastasis and Cisplatin Resistance of OC by Targeting CXCR4 and let-7a/BCL-XL/S Signaling Axis. Mol. Ther. Nucleic Acids.

[294] Du Z., Sha X. (2017). Demethoxycurcumin inhibited human epithelia ovarian cancer cells′ growth via up-regulating miR-551a. Tumour Biol..

[295] Zhao H., Wang A., Zhang Z. (2020). LncRNA SDHAP1 confers paclitaxel resistance of ovarian cancer by regulating EIF4G2 expression via miR-4465. J. Biochem..

[296] Wu B., Zhang L., Yu Y., Lu T., Zhang Y., Zhu W., Song Q., Lv C., Guo J., Tian Y. (2020). miR-6086 inhibits ovarian cancer angiogenesis by downregulating the OC2/VEGFA/EGFL6 axis. Cell Death Dis..

[297] Van Jaarsveld M.T., van Kuijk P.F., Boersma A.W., Helleman J., van Ijcken W.F., Mathijssen R.H., Pothof J., Berns E.M., Verweij J., Wiemer E.A. (2015). miR-634 restores drug sensitivity in resistant ovarian cancer cells by targeting the Ras-MAPK pathway. Mol. Cancer.

[298] Gao J., Wu N., Liu X., Xia Y., Chen Y., Li S., Deng Z. (2018). MicroRNA-142-3p inhibits cell proliferation and chemoresistance in ovarian cancer via targeting sirtuin 1. Exp. Ther. Med..

[299] Hiroki E., Akahira J., Suzuki F., Nagase S., Ito K., Suzuki T., Sasano H., Yaegashi N. (2010). Changes in microRNA expression levels correlate with clinicopathological features and prognoses in endometrial serous adenocarcinomas. Cancer Sci..

[300] Karaayvaz M., Zhang C., Liang S., Shroyer K.R., Ju J. (2012). Prognostic significance of miR-205 in endometrial cancer. PLoS ONE.

[301] Torres A., Torres K., Pesci A., Ceccaroni M., Paszkowski T., Cassandrini P., Zamboni G., Maciejewski R. (2013). Diagnostic and prognostic significance of miRNA signatures in tissues and plasma of endometrioid endometrial carcinoma patients. Int. J. Cancer.

[302] Wang Y., Xu M., Yang Q. (2019). A six-microRNA signature predicts survival of patients with uterine corpus endometrial carcinoma. Curr. Probl. Cancer.

[303] Zhang X., Dong Y., Ti H., Zhao J., Wang Y., Li T., Zhang B. (2013). Down-regulation of miR-145 and miR-143 might be associated with DNA methyltransferase 3B overexpression and worse prognosis in endometrioid carcinomas. Hum. Pathol..

[304] Canlorbe G., Castela M., Bendifallah S., Wang Z., Lefevre M., Chabbert-Buffet N., Aractingi S., Dara I.E., Mehats C., Ballester M. (2017). Micro-RNA signature of lymphovascular space involvement in type 1 endometrial cancer. Histol. Histopathol..

[305] Srivastava S.K., Ahmad A., Zubair H., Miree O., Singh S., Rocconi R.P., Scalici J., Singh A.P. (2017). MicroRNAs in gynecological cancers: Small molecules with big implications. Cancer Lett..

[306] Chen S., Sun K.X., Liu B.L., Zong Z.H., Zhao Y. (2016). MicroRNA-505 functions as a tumor suppressor in endometrial cancer by targeting TGF-alpha. Mol. Cancer.

[307] Zhou H., Xu X., Xun Q., Yu D., Ling J., Guo F., Yan Y., Shi J., Hu Y. (2012). microRNA-30c negatively regulates endometrial cancer cells by targeting metastasis-associated gene-1. Oncol. Rep..

[308] Canlorbe G., Wang Z., Laas E., Bendifallah S., Castela M., Lefevre M., Chabbert-Buffet N., Darai E., Aractingi S., Mehats C. (2016). Identification of microRNA expression profile related to lymph node status in women with early-stage grade 1-2 endometrial cancer. Mod. Pathol..

[309] Wu X., Han Y., Liu F., Ruan L. (2020). Downregulations of miR-449a and miR-145-5p Act as Prognostic Biomarkers for Endometrial Cancer. J. Comput. Biol..

[310] Park Y.A., Lee J.W., Choi J.J., Jeon H.K., Cho Y., Choi C., Kim T.J., Lee N.W., Kim B.G., Bae D.S. (2012). The interactions between MicroRNA-200c and BRD7 in endometrial carcinoma. Gynecol. Oncol..

[311] Wu Q., Lu R.L., Li J.X., Rong L.J. (2017). MiR-200a and miR-200b target PTEN to regulate the endometrial cancer cell growth in vitro. Asian Pac. J. Trop. Med..

[312] Qin X., Yan L., Zhao X., Li C., Fu Y. (2012). microRNA-21 overexpression contributes to cell proliferation by targeting PTEN in endometrioid endometrial cancer. Oncol. Lett..

[313] Tan Z.Q., Liu F.X., Tang H.L., Su Q. (2010). Expression and its clinical significance of hsa-miR-155 in serum of endometrial cancer. Zhonghua Fu Chan Ke Za Zhi.

[314] Kottaridi C., Spathis A., Margari N., Koureas N., Terzakis E., Chrelias C., Pappas A., Bilirakis E., Pouliakis A., Panayiotides I.J. (2017). Evaluation Analysis of miRNAs Overexpression in Liquid-Based Cytology Endometrial Samples. J. Cancer.

[315] Lee H., Choi H.J., Kang C.S., Lee H.J., Lee W.S., Park C.S. (2012). Expression of miRNAs and PTEN in endometrial specimens ranging from histologically normal to hyperplasia and endometrial adenocarcinoma. Mod. Pathol..

[316] Li B.L., Lu C., Lu W., Yang T.T., Qu J., Hong X., Wan X.P. (2013). miR-130b is an EMT-related microRNA that targets DICER1 for aggression in endometrial cancer. Med. Oncol..

[317] Ruan H., Liang X., Zhao W., Ma L., Zhao Y. (2017). The effects of microRNA-183 promots cell proliferation and invasion by targeting MMP-9 in endometrial cancer. Biomed. Pharmacother..

[318] Huang C., Hu G. (2018). Shikonin suppresses proliferation and induces apoptosis in endometrioid endometrial cancer cells via modulating miR-106b/PTEN/AKT/mTOR signaling pathway. Biosci. Rep..

[319] Chen H., Fan Y., Xu W., Chen J., Xu C., Wei X., Fang D., Feng Y. (2016). miR-10b Inhibits Apoptosis and Promotes Proliferation and Invasion of Endometrial Cancer Cells via Targeting HOXB3. Cancer Biother. Radiopharm..

[320] Yang L., Yang Z., Yao R., Li Y., Liu Z., Chen X., Zhang G. (2018). miR-210 promotes progression of endometrial carcinoma by regulating the expression of NFIX. Int. J. Clin. Exp. Pathol..

[321] Yang C., Ota-Kurogi N., Ikeda K., Okumura T., Horie-Inoue K., Takeda S., Inoue S. (2020). MicroRNA-191 regulates endometrial cancer cell growth via TET1-mediated epigenetic modulation of APC. J. Biochem..

[322] Montagnana M., Benati M., Danese E., Giudici S., Perfranceschi M., Ruzzenenete O., Salvagno G.L., Bassi A., Gelati M., Paviati E. (2017). Aberrant MicroRNA Expression in Patients with Endometrial Cancer. Int. J. Gynecol. Cancer.

[323] Zhang H.C., Han Y.Y., Zhang X.M., Xiao N., Jiang T., Zhu S., Wang E.P., Chen C.B. (2019). miR-522 facilitates the prosperities of endometrial carcinoma cells by directly binding to monoamine oxidase B. Kaohsiung J. Med. Sci..

[324] Zhou Z., Xu Y.P., Wang L.J., Kong Y. (2019). miR-940 potentially promotes proliferation and metastasis of endometrial carcinoma through regulation of MRVI1. Biosci. Rep..

[325] He Z., Xu H., Meng Y., Kuang Y. (2017). miR-944 acts as a prognostic marker and promotes the tumor progression in endometrial cancer. Biomed. Pharmacother..

[326] Xie D., Liang Y., Su Y., An Y., Qu P. (2018). miR-152 inhibits proliferation of human endometrial cancer cells via inducing G2/M phase arrest by suppressing CDC25B expression. Biomed. Pharmacother..

[327] Yuan D.Z., Lei Y., Zhao D., Pan J.L., Zhao Y.B., Nie L., Liu M., Long Y., Zhang J.H., Yue L.M. (2019). Progesterone-Induced miR-145/miR-143 Inhibits the Proliferation of Endometrial Epithelial Cells. Reprod. Sci..

[328] Guo S., Yang J., Wu M., Xiao G. (2019). Clinical value screening, prognostic significance and key pathway identification of miR-204-5p in endometrial carcinoma: A study based on the Cancer Genome Atlas (TCGA), and bioinformatics analysis. Pathol. Res. Pract..

[329] Chang L., Zhang D., Shi H., Bian Y., Guo R. (2017). MiR-143 inhibits endometrial cancer cell proliferation and metastasis by targeting MAPK1. Oncotarget.

[330] Huang Y.W., Kuo C.T., Chen J.H., Goodfellow P.J., Huang T.H., Rader J.S., Uyar D.S. (2014). Hypermethylation of miR-203 in endometrial carcinomas. Gynecol. Oncol..

[331] Dong P., Xiong Y., Yue J., Hanley S.J.B., Watari H. (2018). miR-34a, miR-424 and miR-513 inhibit MMSET expression to repress endometrial cancer cell invasion and sphere formation. Oncotarget.

[332] Sun J., Gao S., Lu C. (2019). Knockdown of differentiation antagonizing non-protein coding RNA exerts anti-tumor effect by up-regulating miR-214 in endometrial carcinoma. Mol. Cell Biochem..

[333] Jayaraman M., Radhakrishnan R., Mathews C.A., Yan M., Husain S., Moxley K.M., Song Y.S., Dhanasekaran D.N. (2017). Identification of novel diagnostic and prognostic miRNA signatures in endometrial cancer. Genes Cancer.

[334] Dong P., Karaayvaz M., Jia N., Kaneuchi M., Hamada J., Watari H., Sudo S., Ju J., Sakuragi N. (2013). Mutant p53 gain-of-function induces epithelial-mesenchymal transition through modulation of the miR-130b-ZEB1 axis. Oncogene.

[335] Zhao X., Zhu D., Lu C., Yan D., Li L., Chen Z. (2016). MicroRNA-126 inhibits the migration and invasion of endometrial cancer cells by targeting insulin receptor substrate 1. Oncol. Lett..

[336] Zhao X., Dai L., Yue Q., Wang H., Wang X.U., Li Y., Chen R. (2019). MiR-195 inhibits migration, invasion and epithelial-mesenchymal transition (EMT) of endometrial carcinoma cells by targeting SOX4. J. Biosci..

[337] Zhang W., Chen J.H., Shan T., Aguilera-Barrantes I., Wang L.S., Huang T.H., Rader J.S., Sheng X., Huang Y.W. (2018). miR-137 is a tumor suppressor in endometrial cancer and is repressed by DNA hypermethylation. Lab. Investig..

[338] Wu Y.S., Lin H., Chen D., Yi Z., Zeng B., Jiang Y., Ren G. (2019). A four-miRNA signature as a novel biomarker for predicting survival in endometrial cancer. Gene.

[339] Su Y., Wang J., Ma Z., Gong W., Yu L. (2019). miR-142 Suppresses Endometrial Cancer Proliferation In Vitro and In Vivo by Targeting Cyclin D1. DNA Cell Biol..

[340] Devor E.J., Miecznikowski J., Schickling B.M., Gonzalez-Bosquet J., Lankes H.A., Thaker P., Argenta P.A., Pearl M.L., Zweizig S.L., Mannel R.S. (2017). Dysregulation of miR-181c expression influences recurrence of endometrial endometrioid adenocarcinoma by modulating NOTCH2 expression: An NRG Oncology/Gynecologic Oncology Group study. Gynecol. Oncol..

[341] Li H.L., Sun J.J., Ma H., Liu S.J., Li N., Guo S.J., Shi Y., Xu Y.Y., Qi Z.Y., Wang Y.Q. (2019). MicroRNA-23a inhibits endometrial cancer cell development by targeting SIX1. Oncol. Lett..

[342] Van Sinderen M., Griffiths M., Menkhorst E., Niven K., Dimitriadis E. (2019). Restoration of microRNA-29c in type I endometrioid cancer reduced endometrial cancer cell growth. Oncol. Lett..

[343] Shu S., Liu X., Xu M., Gao X., Chen S., Zhang L., Li R. (2019). MicroRNA-320a acts as a tumor suppressor in endometrial carcinoma by targeting IGF-1R. Int. J. Mol. Med..

[344] Giglio S., Annibali V., Cirombella R., Faruq O., Volinia S., De Vitis C., Pesce M., Caserta D., Pettinato A., Fraggetta F. (2019). miRNAs as Candidate Biomarker for the Accurate Detection of Atypical Endometrial Hyperplasia/Endometrial Intraepithelial Neoplasia. Front. Oncol..

[345] Tian Y., Chen Y.Y., Han A.L. (2019). MiR-1271 inhibits cell proliferation and metastasis by targeting LDHA in endometrial cancer. Eur. Rev. Med. Pharmacol. Sci..

[346] Wu J., Qian J., Li C., Kwok L., Cheng F., Liu P., Perdomo C., Kotton D., Vaziri C., Anderlind C. (2010). miR-129 regulates cell proliferation by downregulating Cdk6 expression. Cell Cycle.

[347] Qu J., Zhang L., Li L., Su Y. (2018). miR-148b Functions as a Tumor Suppressor by Targeting Endoplasmic Reticulum Metallo Protease 1 in Human Endometrial Cancer Cells. Oncol. Res..

[348] Chen P., Xing T., Wang Q., Liu A., Liu H., Hu Y., Ji Y., Song Y., Wang D. (2019). MicroRNA-202 inhibits cell migration and invasion through targeting FGF2 and inactivating Wnt/beta-catenin signaling in endometrial carcinoma. Biosci. Rep..

[349] Zheng Y., Yang X., Wang C., Zhang S., Wang Z., Li M., Wang Y., Wang X., Yang X. (2020). HDAC6, modulated by miR-206, promotes endometrial cancer progression through the PTEN/AKT/mTOR pathway. Sci. Rep..

[350] Zhou Y.X., Wang C., Mao L.W., Wang Y.L., Xia L.Q., Zhao W., Shen J., Chen J. (2018). Long noncoding RNA HOTAIR mediates the estrogen-induced metastasis of endometrial cancer cells via the miR-646/NPM1 axis. Am. J. Physiol. Cell Physiol..

[351] Shi F., Wang T., Liu Z., Zhang Y., Wang J., Zhang K., Su J. (2019). LncRNA miR143HG Up-Regulates p53 In Endometrial Carcinoma by Sponging miR-125a. Cancer Manag. Res..

[352] Lu M., Ding N., Zhuang S., Li Y. (2020). LINC01410/miR-23c/CHD7 functions as a ceRNA network to affect the prognosis of patients with endometrial cancer and strengthen the malignant properties of endometrial cancer cells. Mol. Cell Biochem..

[353] Wang Q., Zhu W. (2019). MicroRNA-873 inhibits the proliferation and invasion of endometrial cancer cells by directly targeting hepatoma-derived growth factor. Exp. Ther. Med..

[354] Saraiya M., Unger E.R., Thompson T.D., Lynch C.F., Hernandez B.Y., Lyu C.W., Steinau M., Watson M., Wilkinson E.J., Hopenhayn C. (2015). US assessment of HPV types in cancers: Implications for current and 9-valent HPV vaccines. J. Natl. Cancer Inst..

[355] Yao Q., Xu H., Zhang Q.Q., Zhou H., Qu L.H. (2009). MicroRNA-21 promotes cell proliferation and down-regulates the expression of programmed cell death 4 (PDCD4) in HeLa cervical carcinoma cells. Biochem. Biophys. Res. Commun..

[356] Zhao S., Yao D., Chen J., Ding N., Ren F. (2015). MiR-20a promotes cervical cancer proliferation and metastasis in vitro and in vivo. PLoS ONE.

[357] Li X., Zhou Q., Tao L., Yu C. (2017). MicroRNA-106a promotes cell migration and invasion by targeting tissue inhibitor of matrix metalloproteinase 2 in cervical cancer. Oncol. Rep..

[358] Zhao S., Yao D., Chen J., Ding N. (2013). Circulating miRNA-20a and miRNA-203 for screening lymph node metastasis in early stage cervical cancer. Genet. Test. Mol. Biomark..

[359] Tao P., Wen H., Yang B., Zhang A., Wu X., Li Q. (2018). miR-144 inhibits growth and metastasis of cervical cancer cells by targeting VEGFA and VEGFC. Exp. Ther. Med..

[360] Zhou Q., Han L.R., Zhou Y.X., Li Y. (2016). MiR-195 Suppresses Cervical Cancer Migration and Invasion Through Targeting Smad3. Int. J. Gynecol. Cancer.

[361] Yang Y.K., Xi W.Y., Xi R.X., Li J.Y., Li Q., Gao Y.E. (2015). MicroRNA-494 promotes cervical cancer proliferation through the regulation of PTEN. Oncol. Rep..

[362] Zhang Z., Wang J., Wang X., Song W., Shi Y., Zhang L. (2018). MicroRNA-21 promotes proliferation, migration, and invasion of cervical cancer through targeting TIMP3. Arch. Gynecol. Obstet..

[363] Zhang Z., Wang J., Li J., Wang X., Song W. (2018). MicroRNA-150 promotes cell proliferation, migration, and invasion of cervical cancer through targeting PDCD4. Biomed. Pharmacother..

[364] Zhou X., Yue Y., Wang R., Gong B., Duan Z. (2017). MicroRNA-145 inhibits tumorigenesis and invasion of cervical cancer stem cells. Int. J. Oncol..

[365] Liu S., Song L., Zeng S., Zhang L. (2016). MALAT1-miR-124-RBG2 axis is involved in growth and invasion of HR-HPV-positive cervical cancer cells. Tumour Biol..

[366] Zhou C., Shen L., Mao L., Wang B., Li Y., Yu H. (2015). miR-92a is upregulated in cervical cancer and promotes cell proliferation and invasion by targeting FBXW7. Biochem. Biophys. Res. Commun..

[367] Wang L., Chang L., Li Z., Gao Q., Cai D., Tian Y., Zeng L., Li M. (2014). miR-99a and -99b inhibit cervical cancer cell proliferation and invasion by targeting mTOR signaling pathway. Med. Oncol..

[368] Wang F., Li Y., Zhou J., Xu J., Peng C., Ye F., Shen Y., Lu W., Wan X., Xie X. (2011). miR-375 is down-regulated in squamous cervical cancer and inhibits cell migration and invasion via targeting transcription factor SP1. Am. J. Pathol..

[369] Tang B.B., Liu S.Y., Zhan Y.U., Wei L.Q., Mao X.L., Wang J., Li L.I., Lu Z.X. (2015). microRNA-218 expression and its association with the clinicopathological characteristics of patients with cervical cancer. Exp. Ther. Med..

[370] Jia W., Wu Y., Zhang Q., Gao G.E., Zhang C., Xiang Y. (2015). Expression profile of circulating microRNAs as a promising fingerprint for cervical cancer diagnosis and monitoring. Mol. Clin. Oncol..

[371] Wang W.T., Zhao Y.N., Yan J.X., Weng M.Y., Wang Y., Chen Y.Q., Hong S.J. (2014). Differentially expressed microRNAs in the serum of cervical squamous cell carcinoma patients before and after surgery. J. Hematol. Oncol..

[372] Zhang R., Su J., Xue S.L., Yang H., Ju L.L., Ji Y., Wu K.H., Zhang Y.W., Zhang Y.X., Hu J.F. (2016). HPV E6/p53 mediated down-regulation of miR-34a inhibits Warburg effect through targeting LDHA in cervical cancer. Am. J. Cancer Res..

[373] Geng D., Song X., Ning F., Song Q., Yin H. (2015). MiR-34a Inhibits Viability and Invasion of Human Papillomavirus-Positive Cervical Cancer Cells by Targeting E2F3 and Regulating Survivin. Int. J. Gynecol. Cancer.

[374] Wang X., Meyers C., Guo M., Zheng Z.M. (2011). Upregulation of p18Ink4c expression by oncogenic HPV E6 via p53-miR-34a pathway. Int. J. Cancer.

[375] Pang R.T., Leung C.O., Ye T.M., Liu W., Chiu P.C., Lam K.K., Lee K.F., Yeung W.S. (2010). MicroRNA-34a suppresses invasion through downregulation of Notch1 and Jagged1 in cervical carcinoma and choriocarcinoma cells. Carcinogenesis.

[376] Wang X., Wang H.K., McCoy J.P., Banerjee N.S., Rader J.S., Broker T.R., Meyers C., Chow L.T., Zheng Z.M. (2009). Oncogenic HPV infection interrupts the expression of tumor-suppressive miR-34a through viral oncoprotein E6. RNA.

[377] Hayes J., Peruzzi P.P., Lawler S. (2014). MicroRNAs in cancer: Biomarkers, functions and therapy. Trends Mol. Med..

[378] Yu L., Xiong J., Guo L., Miao L., Liu S., Guo F. (2015). The effects of lanthanum chloride on proliferation and apoptosis of cervical cancer cells: Involvement of let-7a and miR-34a microRNAs. Biometals.

[379] Liu W., Gao G., Hu X., Wang Y., Schwarz J.K., Chen J.J., Grigsby P.W., Wang X. (2014). Activation of miR-9 by human papillomavirus in cervical cancer. Oncotarget.

[380] Liu J., Sun H., Wang X., Yu Q., Li S., Yu X., Gong W. (2014). Increased exosomal microRNA-21 and microRNA-146a levels in the cervicovaginal lavage specimens of patients with cervical cancer. Int. J. Mol. Sci..

[381] Harden M.E., Munger K. (2017). Human papillomavirus 16 E6 and E7 oncoprotein expression alters microRNA expression in extracellular vesicles. Virology.

[382] Greco D., Kivi N., Qian K., Leivonen S.K., Auvinen P., Auvinen E. (2011). Human papillomavirus 16 E5 modulates the expression of host microRNAs. PLoS ONE.

[383] Park S., Eom K., Kim J., Bang H., Wang H.Y., Ahn S., Kim G., Jang H., Kim S., Lee D. (2017). MiR-9, miR-21, and miR-155 as potential biomarkers for HPV positive and negative cervical cancer. BMC Cancer.

[384] Xie H., Zhao Y., Caramuta S., Larsson C., Lui W.O. (2012). miR-205 expression promotes cell proliferation and migration of human cervical cancer cells. PLoS ONE.

[385] Ma Q., Wan G., Wang S., Yang W., Zhang J., Yao X. (2014). Serum microRNA-205 as a novel biomarker for cervical cancer patients. Cancer Cell Int..

[386] Zeng F., Xue M., Xiao T., Li Y., Xiao S., Jiang B., Ren C. (2016). MiR-200b promotes the cell proliferation and metastasis of cervical cancer by inhibiting FOXG1. Biomed. Pharmacother..

[387] Wang L., Wang Q., Li H.L., Han L.Y. (2013). Expression of MiR200a, miR93, metastasis-related gene RECK and MMP2/MMP9 in human cervical carcinoma--relationship with prognosis. Asian Pac. J. Cancer Prev..

[388] Park S., Kim J., Eom K., Oh S., Kim S., Kim G., Ahn S., Park K.H., Chung D., Lee H. (2019). microRNA-944 overexpression is a biomarker for poor prognosis of advanced cervical cancer. BMC Cancer.

[389] Yin S., Zhang Q., Wang Y., Li S., Hu R. (2019). MicroRNA-130a regulated by HPV18 E6 promotes proliferation and invasion of cervical cancer cells by targeting TIMP2. Exp. Ther. Med..

[390] Cheng Y., Guo Y., Zhang Y., You K., Li Z., Geng L. (2016). MicroRNA-106b is involved in transforming growth factor beta1-induced cell migration by targeting disabled homolog 2 in cervical carcinoma. J. Exp. Clin. Cancer Res..

[391] Yao J., Deng B., Zheng L., Dou L., Guo Y., Guo K. (2016). miR-27b is upregulated in cervical carcinogenesis and promotes cell growth and invasion by regulating CDH11 and epithelial-mesenchymal transition. Oncol. Rep..

[392] Wang X., Tang S., Le S.Y., Lu R., Rader J.S., Meyers C., Zheng Z.M. (2008). Aberrant expression of oncogenic and tumor-suppressive microRNAs in cervical cancer is required for cancer cell growth. PLoS ONE.

[393] Qin W., Dong P., Ma C., Mitchelson K., Deng T., Zhang L., Sun Y., Feng X., Ding Y., Lu X. (2012). MicroRNA-133b is a key promoter of cervical carcinoma development through the activation of the ERK and AKT1 pathways. Oncogene.

[394] Morgan E.L., Patterson M.R., Ryder E.L., Lee S.Y., Wasson C.W., Harper K.L., Li Y., Griffin S., Blair G.E., Whitehouse A. (2020). MicroRNA-18a targeting of the STK4/MST1 tumour suppressor is necessary for transformation in HPV positive cervical cancer. PLoS Pathog..

[395] Chen Y., Song Y., Mi Y., Jin H., Cao J., Li H., Han L., Huang T., Zhang X., Ren S. (2020). microRNA-499a promotes the progression and chemoresistance of cervical cancer cells by targeting SOX6. Apoptosis.

[396] Yang D., Zhang Q. (2019). miR-152 may function as an early diagnostic and prognostic biomarker in patients with cervical intraepithelial neoplasia and patients with cervical cancer. Oncol. Lett..

[397] Xu Y., Zhao S., Cui M., Wang Q. (2015). Down-regulation of microRNA-135b inhibited growth of cervical cancer cells by targeting FOXO1. Int. J. Clin. Exp. Pathol..

[398] Liu S.S., Chan K.K.L., Chu D.K.H., Wei T.N., Lau L.S.K., Ngu S.F., Chu M.M.Y., Tse K.Y., Ip P.P.C., Ng E.K.O. (2018). Oncogenic microRNA signature for early diagnosis of cervical intraepithelial neoplasia and cancer. Mol. Oncol..

[399] Su K., Wang C.F., Zhang Y., Cai Y.J., Zhang Y.Y., Zhao Q. (2017). miR-940 upregulation contributes to human cervical cancer progression through p27 and PTEN inhibition. Int. J. Oncol..

[400] Hasanzadeh M., Movahedi M., Rejali M., Maleki F., Moetamani-Ahmadi M., Seifi S., Hosseini Z., Khazaei M., Amerizadeh F., Ferns G.A. (2019). The potential prognostic and therapeutic application of tissue and circulating microRNAs in cervical cancer. J. Cell Physiol..

[401] Yang L., Wang Y., Shi S., Xie L., Liu T., Wang Y., Mu H. (2018). The TNF-alpha-induced expression of miR-130b protects cervical cancer cells from the cytotoxicity of TNF-alpha. FEBS Open Bio..

[402] Nagamitsu Y., Nishi H., Sasaki T., Takaesu Y., Terauchi F., Isaka K. (2016). Profiling analysis of circulating microRNA expression in cervical cancer. Mol. Clin. Oncol..

[403] Wang L.Q., Zhang Y., Yan H., Liu K.J., Zhang S. (2015). MicroRNA-373 functions as an oncogene and targets YOD1 gene in cervical cancer. Biochem. Biophys. Res. Commun..

[404] Li Y., Wang H., Huang H. (2019). Long non-coding RNA MIR205HG function as a ceRNA to accelerate tumor growth and progression via sponging miR-122-5p in cervical cancer. Biochem. Biophys. Res. Commun..

[405] You W., Wang Y., Zheng J. (2015). Plasma miR-127 and miR-218 Might Serve as Potential Biomarkers for Cervical Cancer. Reprod. Sci..

[406] Pardini B., De Maria D., Francavilla A., Di Gaetano C., Ronco G., Naccarati A. (2018). MicroRNAs as markers of progression in cervical cancer: A systematic review. BMC Cancer.

[407] Li W.T., Wang B.L., Yang C.S., Lang B.C., Lin Y.Z. (2018). MiR-613 promotes cell proliferation and invasion in cervical cancer via targeting PTPN9. Eur. Rev. Med. Pharmacol. Sci..

[408] Pereira P.M., Marques J.P., Soares A.R., Carreto L., Santos M.A. (2010). MicroRNA expression variability in human cervical tissues. PLoS ONE.

[409] Sanches J.G.P., Xu Y., Yabasin I.B., Li M., Lu Y., Xiu X., Wang L., Mao L., Shen J., Wang B. (2018). miR-501 is upregulated in cervical cancer and promotes cell proliferation, migration and invasion by targeting CYLD. Chem. Biol. Interact..

[410] Luo Q., Wang H., Li J. (2019). Serum miR-3142 could be Used as a Potential Biomarker to Screen Cervical Cancer Patients from Healthy Controls. Clin. Lab..

[411] Zhou J.Y., Zheng S.R., Liu J., Shi R., Yu H.L., Wei M. (2016). MiR-519d facilitates the progression and metastasis of cervical cancer through direct targeting Smad7. Cancer Cell Int..

[412] Phuah N.H., Azmi M.N., Awang K., Nagoor N.H. (2017). Suppression of microRNA-629 enhances sensitivity of cervical cancer cells to 1′S-1′-acetoxychavicol acetate via regulating RSU1. OncoTargets Ther..

[413] Zhu J., Han S. (2019). Lidocaine inhibits cervical cancer cell proliferation and induces cell apoptosis by modulating the lncRNA-MEG3/miR-421/BTG1 pathway. Am. J. Transl. Res..

[414] Wei H., Wen-Ming C., Jun-Bo J. (2017). Plasma miR-145 as a novel biomarker for the diagnosis and radiosensitivity prediction of human cervical cancer. J. Int. Med. Res..

[415] Dong Z., Yu C., Rezhiya K., Gulijiahan A., Wang X. (2019). Downregulation of miR-146a promotes tumorigenesis of cervical cancer stem cells via VEGF/CDC42/PAK1 signaling pathway. Artif. Cells Nanomed. Biotechnol..

[416] Zhao S., Yao D.S., Chen J.Y., Ding N. (2013). Aberrant expression of miR-20a and miR-203 in cervical cancer. Asian Pac. J. Cancer Prev..

[417] Peng R., Cheng X., Zhang Y., Lu X., Hu Z. (2020). miR-214 down-regulates MKK3 and suppresses malignant phenotypes of cervical cancer cells. Gene.

[418] Du X., Lin L.I., Zhang L., Jiang J. (2015). microRNA-195 inhibits the proliferation, migration and invasion of cervical cancer cells via the inhibition of CCND2 and MYB expression. Oncol. Lett..

[419] Nan P., Niu Y., Wang X., Li Q. (2019). MiR-29a function as tumor suppressor in cervical cancer by targeting SIRT1 and predict patient prognosis. OncoTargets Ther..

[420] Zhou Y., An Q., Guo R.X., Qiao Y.H., Li L.X., Zhang X.Y., Zhao X.L. (2017). miR424-5p functions as an anti-oncogene in cervical cancer cell growth by targeting KDM5B via the Notch signaling pathway. Life Sci..

[421] Hao Z., Yang J., Wang C., Li Y., Zhang Y., Dong X., Zhou L., Liu J., Zhang Y., Qian J. (2015). MicroRNA-7 inhibits metastasis and invasion through targeting focal adhesion kinase in cervical cancer. Int. J. Clin. Exp. Med..

[422] Wang Y., Tian Y. (2018). miR-206 Inhibits Cell Proliferation, Migration, and Invasion by Targeting BAG3 in Human Cervical Cancer. Oncol. Res..

[423] Wongjampa W., Ekalaksananan T., Chopjitt P., Chuerduangphui J., Kleebkaow P., Patarapadungkit N., Pientong C. (2018). Suppression of miR-22, a tumor suppressor in cervical cancer, by human papillomavirus 16 E6 via a p53/miR-22/HDAC6 pathway. PLoS ONE.

[424] Wang W., Li Y., Liu N., Gao Y., Li L. (2017). MiR-23b controls ALDH1A1 expression in cervical cancer stem cells. BMC Cancer.

[425] Sun J., Ji J., Huo G., Song Q., Zhang X. (2015). miR-182 induces cervical cancer cell apoptosis through inhibiting the expression of DNMT3a. Int. J. Clin. Exp. Pathol..

[426] Luo M., Shen D., Zhou X., Chen X., Wang W. (2013). MicroRNA-497 is a potential prognostic marker in human cervical cancer and functions as a tumor suppressor by targeting the insulin-like growth factor 1 receptor. Surgery.

[427] Fan D., Wang Y., Qi P., Chen Y., Xu P., Yang X., Jin X., Tian X. (2016). MicroRNA-183 functions as the tumor suppressor via inhibiting cellular invasion and metastasis by targeting MMP-9 in cervical cancer. Gynecol. Oncol..

[428] Fan Z., Cui H., Xu X., Lin Z., Zhang X., Kang L., Han B., Meng J., Yan Z., Yan X. (2015). MiR-125a suppresses tumor growth, invasion and metastasis in cervical cancer by targeting STAT3. Oncotarget.

[429] Shu L., Zhang Z., Cai Y. (2018). MicroRNA-204 inhibits cell migration and invasion in human cervical cancer by regulating transcription factor 12. Oncol. Lett..

[430] Wen S.Y., Lin Y., Yu Y.Q., Cao S.J., Zhang R., Yang X.M., Li J., Zhang Y.L., Wang Y.H., Ma M.Z. (2015). miR-506 acts as a tumor suppressor by directly targeting the hedgehog pathway transcription factor Gli3 in human cervical cancer. Oncogene.

[431] Liang H., Luo R., Chen X., Zhao Y., Tan A. (2017). miR-187 inhibits the growth of cervical cancer cells by targeting FGF9. Oncol. Rep..

[432] Ou R., Zhu L., Zhao L., Li W., Tao F., Lu Y., He Q., Li J., Ren Y., Xu Y. (2019). HPV16 E7-induced upregulation of KDM2A promotes cervical cancer progression by regulating miR-132-radixin pathway. J. Cell Physiol..

[433] Tang Y., Wang Y., Chen Q., Qiu N., Zhao Y., You X. (2015). MiR-223 inhibited cell metastasis of human cervical cancer by modulating epithelial-mesenchymal transition. Int. J. Clin. Exp. Pathol..

[434] Zhang J., Wang Q., Quan Z. (2019). Long non-coding RNA CASC9 enhances breast cancer progression by promoting metastasis through the meditation of miR-215/TWIST2 signaling associated with TGF-beta expression. Biochem. Biophys. Res. Commun..

[435] He S., Liao B., Deng Y., Su C., Tuo J., Liu J., Yao S., Xu L. (2017). MiR-216b inhibits cell proliferation by targeting FOXM1 in cervical cancer cells and is associated with better prognosis. BMC Cancer.

[436] Zou D., Zhou Q., Wang D., Guan L., Yuan L., Li S. (2016). The Downregulation of MicroRNA-10b and its Role in Cervical Cancer. Oncol. Res..

[437] Wang L., Wang W., Wu Y. (2019). MicroRNA-26b acts as an antioncogene and prognostic factor in cervical cancer. Oncol. Lett..

[438] Dong J., Wang M., Ni D., Zhang L., Wang W., Cui X., Fu S., Yao S. (2018). MicroRNA-217 functions as a tumor suppressor in cervical cancer cells through targeting Rho-associated protein kinase 1. Oncol. Lett..

[439] Wang S., Gao B., Yang H., Liu X., Wu X., Wang W. (2019). MicroRNA-432 is downregulated in cervical cancer and directly targets FN1 to inhibit cell proliferation and invasion. Oncol. Lett..

[440] Yao R., Zheng H., Wu L., Cai P. (2018). miRNA-641 inhibits the proliferation, migration, and invasion and induces apoptosis of cervical cancer cells by directly targeting ZEB1. OncoTargets Ther..

[441] Chen X.F., Liu Y. (2016). MicroRNA-744 inhibited cervical cancer growth and progression through apoptosis induction by regulating Bcl-2. Biomed. Pharmacother..

[442] Liu Z., Wu M., Shi H., Huang C., Luo S., Song X. (2019). DDN-AS1-miR-15a/16-TCF3 feedback loop regulates tumor progression in cervical cancer. J. Cell Biochem..

[443] Mei J., Wang D.H., Wang L.L., Chen Q., Pan L.L., Xia L. (2018). MicroRNA-200c suppressed cervical cancer cell metastasis and growth via targeting MAP4K4. Eur. Rev. Med. Pharmacol. Sci..

[444] Liu C., Wang J., Hu Y., Xie H., Liu M., Tang H. (2017). Upregulation of kazrin F by miR-186 suppresses apoptosis but promotes epithelial-mesenchymal transition to contribute to malignancy in human cervical cancer cells. Chin. J. Cancer Res..

[445] Zhao J., Li B., Shu C., Ma Y., Gong Y. (2017). Downregulation of miR-30a is associated with proliferation and invasion via targeting MEF2D in cervical cancer. Oncol. Lett..

[446] Jiang D., Wang H., Li Z., Li Z., Chen X., Cai H. (2017). MiR-142 inhibits the development of cervical cancer by targeting HMGB1. Oncotarget.

[447] How C., Hui A.B., Alajez N.M., Shi W., Boutros P.C., Clarke B.A., Yan R., Pintilie M., Fyles A., Hedley D.W. (2013). MicroRNA-196b regulates the homeobox B7-vascular endothelial growth factor axis in cervical cancer. PLoS ONE.

[448] Liang L., Zheng Y.W., Wang Y.L. (2020). miR-4429 Regulates the Proliferation, Migration, Invasion, and Epithelial-Mesenchymal Transition of Cervical Cancer by Targeting FOXM1. Cancer Manag. Res..

[449] Chen J., Li G. (2018). MiR-1284 enhances sensitivity of cervical cancer cells to cisplatin via downregulating HMGB1. Biomed. Pharmacother..

[450] Jin Y., Zhou X., Yao X., Zhang Z., Cui M., Lin Y. (2020). MicroRNA-612 inhibits cervical cancer progression by targeting NOB1. J. Cell Mol. Med..

[451] Liang H.X., Li Y.H. (2020). MiR-873, as a suppressor in cervical cancer, inhibits cells proliferation, invasion and migration via negatively regulating ULBP2. Genes Genom..

[452] Li D., Liu S.H., Liu Q.Y., Zou Q.Q., Lv L., Liu G.L., Wu Y. (2020). Analysis of the Role and Regulatory Mechanism of hsa-miR-504 in Cervical Cancer Based on The Cancer Genome Atlas Database. Cancer Biother. Radiopharm..

[453] Zhang Q., Lv R., Guo W., Li X. (2019). microRNA-802 inhibits cell proliferation and induces apoptosis in human cervical cancer by targeting serine/arginine-rich splicing factor 9. J. Cell Biochem..

[454] Li Y.J., Wang Y., Wang Y.Y. (2019). MicroRNA-99b suppresses human cervical cancer cell activity by inhibiting the PI3K/AKT/mTOR signaling pathway. J. Cell Physiol..

[455] Xia N., Tan W.F., Peng Q.Z., Cai H.N. (2019). MiR-374b reduces cell proliferation and cell invasion of cervical cancer through regulating FOXM1. Eur. Rev. Med. Pharmacol. Sci..

[456] Wang Z., He S., Guo P., Guo X., Zheng J. (2017). MicroRNA-1297 inhibits metastasis and epithelial-mesenchymal transition by targeting AEG-1 in cervical cancer. Oncol. Rep..

[457] Xu J., Wan X., Chen X., Fang Y., Cheng X., Xie X., Lu W. (2016). miR-2861 acts as a tumor suppressor via targeting EGFR/AKT2/CCND1 pathway in cervical cancer induced by human papillomavirus virus 16 E6. Sci Rep..

[458] Dou X., Zhou Q., Wen M., Xu J., Zhu Y., Zhang S., Xu X. (2019). Long Noncoding RNA FOXD2-AS1 Promotes the Malignancy of Cervical Cancer by Sponging MicroRNA-760 and Upregulating Hepatoma-Derived Growth Factor. Front. Pharmacol..

[459] Lu H.J., Jin P.Y., Tang Y., Fan S.H., Zhang Z.F., Wang F., Wu D.M., Lu J., Zheng Y.L. (2018). microRNA-136 inhibits proliferation and promotes apoptosis and radiosensitivity of cervical carcinoma through the NF-kappaB pathway by targeting E2F1. Life Sci..

[460] Rong X., Gao W., Yang X., Guo J. (2019). Downregulation of hsa_circ_0007534 restricts the proliferation and invasion of cervical cancer through regulating miR-498/BMI-1 signaling. Life Sci..

[461] Meng X., Zhao Y., Wang J., Gao Z., Geng Q., Liu X. (2017). Regulatory roles of miRNA-758 and matrix extracellular phosphoglycoprotein in cervical cancer. Exp. Ther. Med..

[462] Tian R.Q., Wang X.H., Hou L.J., Jia W.H., Yang Q., Li Y.X., Liu M., Li X., Tang H. (2011). MicroRNA-372 is down-regulated and targets cyclin-dependent kinase 2 (CDK2) and cyclin A1 in human cervical cancer, which may contribute to tumorigenesis. J. Biol. Chem..

[463] Miao H., Wang N., Shi L.X., Wang Z., Song W.B. (2019). Overexpression of mircoRNA-137 inhibits cervical cancer cell invasion, migration and epithelial-mesenchymal transition by suppressing the TGF-beta/smad pathway via binding to GREM1. Cancer Cell Int..

[464] Sun J., Chu H., Ji J., Huo G., Song Q., Zhang X. (2016). Long non-coding RNA HOTAIR modulates HLA-G expression by absorbing miR-148a in human cervical cancer. Int. J. Oncol..

[465] Meng F., Ou J., Liu J., Li X., Meng Y., Yan L., Deng P., Sun B. (2019). MicroRNA-877 is downregulated in cervical cancer and directly targets MACC1 to inhibit cell proliferation and invasion. Exp. Ther. Med..

[466] Sun Y., Cheng Y., Zhang Y., Han K. (2019). MicroRNA-889-3p targets FGFR2 to inhibit cervical cancer cell viability and invasion. Exp. Ther. Med..

[467] Yang X., Yan Z., Yang H., Ni H., Zhang L., Wang Y. (2019). Clinical value of combined detection of miR-1202 and miR-195 in early diagnosis of cervical cancer. Oncol. Lett..

[468] Kan X.Q., Li Y.B., He B., Cheng S., Wei Y., Sun J. (2020). MiR-1294 acts as a tumor inhibitor in cervical cancer by regulating FLOT1 expression. J. Biol. Regul. Homeost. Agents.

[469] Wang H., Xie Y. (2020). BRD7-Mediated miR-3148 Inhibits Progression of Cervical Cancer by Targeting Wnt3a/beta-Catenin Pathway. Reprod. Sci..

[470] Hu Q.L., Xu Z.P., Lan Y.F., Li B. (2020). miR-636 represses cell survival by targeting CDK6/Bcl-2 in cervical cancer. Kaohsiung J. Med. Sci..

[471] Deng Y., Xiong Y., Liu Y. (2016). miR-376c inhibits cervical cancer cell proliferation and invasion by targeting BMI1. Int. J. Exp. Pathol..

[472] Wang T., Feng J., Zhang A. (2020). miR-584 inhibits cell proliferation, migration and invasion in vitro and enhances the sensitivity to cisplatin in human cervical cancer by negatively targeting GLI1. Exp. Ther. Med..

[473] Okoye J.O., Ngokere A.A., Onyenekwe C.C., Erinle C.A. (2019). Comparable expression of miR-let-7b, miR-21, miR-182, miR-145, and p53 in serum and cervical cells: Diagnostic implications for early detection of cervical lesions. Int. J. Health Sci..

[474] Huang L., Gan X., He L., Wang L., Yu J. (2019). Silencing of long non-coding RNA NCK1-AS1 inhibits cell proliferation and migration via inhibition of microRNA-134 in cervical cancer. Exp. Ther. Med..

[475] Ma Z., Cai Y., Zhang L., Tian C., Lyu L. (2020). LINC00319 Promotes Cervical Cancer Progression Via Targeting miR-147a/IGF1R Pathway. Cancer Biother. Radiopharm..

[476] Hu Q., Du K., Mao X., Ning S. (2018). miR-197 is downregulated in cervical carcinogenesis and suppresses cell proliferation and invasion through targeting forkhead box M1. Oncol. Lett..

[477] Chen M., Ai G., Zhou J., Mao W., Li H., Guo J. (2019). circMTO1 promotes tumorigenesis and chemoresistance of cervical cancer via regulating miR-6893. Biomed. Pharmacother..

[478] Song T., Xu A., Zhang Z., Gao F., Zhao L., Chen X., Gao J., Kong X. (2019). CircRNA hsa_circRNA_101996 increases cervical cancer proliferation and invasion through activating TPX2 expression by restraining miR-8075. J. Cell Physiol..

[479] Guo H., Yang S., Li S., Yan M., Li L., Zhang H. (2018). LncRNA SNHG20 promotes cell proliferation and invasion via miR-140-5p-ADAM10 axis in cervical cancer. Biomed. Pharmacother..

[480] Wang Q., Ding J., Nan G., Lyu Y., Ni G. (2019). LncRNA NOC2L-4.1 functions as a tumor oncogene in cervical cancer progression by regulating the miR-630/YAP1 pathway. J. Cell Biochem..

[481] Shen W., Song M., Liu J., Qiu G., Li T., Hu Y., Liu H. (2014). MiR-26a promotes ovarian cancer proliferation and tumorigenesis. PLoS ONE.

[482] Sun T.Y., Xie H.J., He H., Li Z., Kong L.F. (2016). miR-26a inhibits the proliferation of ovarian cancer cells via regulating CDC6 expression. Am. J. Transl. Res..

[483] Dong J., Sui L., Wang Q., Chen M., Sun H. (2014). MicroRNA-26a inhibits cell proliferation and invasion of cervical cancer cells by targeting protein tyrosine phosphatase type IVA 1. Mol. Med. Rep..

[484] Ibrahim F.F., Jamal R., Syafruddin S.E., Ab Mutalib N.S., Saidin S., Mdzin R.R., Hossain Mollah M.M., Mokhtar N.M. (2015). MicroRNA-200c and microRNA-31 regulate proliferation, colony formation, migration and invasion in serous ovarian cancer. J. Ovarian Res..

[485] Mitamura T., Watari H., Wang L., Kanno H., Kitagawa M., Hassan M.K., Kimura T., Tanino M., Nishihara H., Tanaka S. (2014). microRNA 31 functions as an endometrial cancer oncogene by suppressing Hippo tumor suppressor pathway. Mol. Cancer.

[486] Creighton C.J., Fountain M.D., Yu Z., Nagaraja A.K., Zhu H., Khan M., Olokpa E., Zariff A., Gunaratne P.H., Matzuk M.M. (2010). Molecular profiling uncovers a p53-associated role for microRNA-31 in inhibiting the proliferation of serous ovarian carcinomas and other cancers. Cancer Res..

[487] Peng R.Q., Wan H.Y., Li H.F., Liu M., Li X., Tang H. (2012). MicroRNA-214 suppresses growth and invasiveness of cervical cancer cells by targeting UDP-N-acetyl-alpha-D-galactosamine:polypeptide N-acetylgalactosaminyltransferase 7. J. Biol. Chem.

[488] Zhao X., Zhou Y., Chen Y.U., Yu F. (2016). miR-494 inhibits ovarian cancer cell proliferation and promotes apoptosis by targeting FGFR2. Oncol. Lett..

[489] Yuan J., Wang K., Xi M. (2016). MiR-494 Inhibits Epithelial Ovarian Cancer Growth by Targeting c-Myc. Med. Sci. Monit..

[490] Sun C., Li N., Zhou B., Yang Z., Ding D., Weng D., Meng L., Wang S., Zhou J., Ma D. (2013). miR-222 is upregulated in epithelial ovarian cancer and promotes cell proliferation by downregulating P27(kip1.). Oncol. Lett..

[491] Fu X., Li Y., Alvero A., Li J., Wu Q., Xiao Q., Peng Y., Hu Y., Li X., Yan W. (2016). MicroRNA-222-3p/GNAI2/AKT axis inhibits epithelial ovarian cancer cell growth and associates with good overall survival. Oncotarget.

